# One versus two biological parents with mental disorders: Relationship to educational attainment in the next generation

**DOI:** 10.1017/S0033291722003506

**Published:** 2023-11

**Authors:** Anna Sidorchuk, Gustaf Brander, Ana Pérez-Vigil, James J. Crowley, Henrik Larsson, Paul Lichtenstein, David Mataix-Cols, Ashley E. Nordsletten

**Affiliations:** 1 Department of Clinical Neuroscience, Karolinska Institutet, Stockholm, Sweden; 2 Stockholm Health Care Services, Region Stockholm, Stockholm, Sweden; 3 Department of Medical Biochemistry and Microbiology, Uppsala Universitet, Uppsala, Sweden; 4 Department of Child and Adolescent Psychiatry and Psychology, Institute of Neuroscience, Hospital Clínic de Barcelona, Barcelona, Spain; 5 Departments of Genetics and Psychiatry, University of North Carolina at Chapel Hill, Chapel Hill, NC, USA; 6 Department of Medical Epidemiology and Biostatistics, Karolinska Institutet, Stockholm, Sweden; 7 School of Medical Sciences, Örebro Universitet, Örebro, Sweden; 8 Department of Psychiatry, University of Michigan, Ann Arbor, MI, USA

**Keywords:** Anxiety, assortative mating, Autism, depression, educational attainment, intergenerational, OCD, psychopathology, schizophrenia, substance use, non-random mating

## Abstract

**Background:**

Both maternal and, separately, paternal mental illness are associated with diminished academic attainment among children. However, the differential impacts of diagnostic type and degree of parental burden (e.g. one v. both parents affected) on these functional outcomes are unknown.

**Methods:**

Using the Swedish national patient (NPR) and multi-generation (MGR) registers, 2 226 451 children (1 290 157 parental pairs), born 1 January 1973–31 December 1997, were followed through 31 December 2013. Diagnostic status of all cohort members was defined for eleven psychiatric disorders, and families classed by exposure: (1) parents affected with any disorder, (2) parents affected with a disorder group (e.g. neuropsychiatric disorders), and (3) parents affected with a specific disorder (e.g. ADHD). Pairs were further defined as ‘unaffected,’ ‘single-affected,’, or ‘dual-affected.’ Among offspring, the study evaluated fulfillment of four academic milestones, from compulsory (primary) school through University (college). Sensitivity analyses considered the impact of child's own mental health, as well as parental education, on main effects.

**Results:**

Marked reductions in the odds of achievement were observed, emerging at the earliest levels of schooling for both single-affected [adjusted odds ratio (aOR), 0.50; 95% CI 0.49–0.51] and dual-affected (aOR 0.29, 95% CI 0.28–0.30) pairs and persisting thereafter [aOR range (single), 0.52–0.65; aOR range (dual), 0.30–0.40]. This pattern was repeated for analyses within diagnosis/diagnostic group. Main results were robust to adjustment for offspring mental health and parent education level.

**Conclusions:**

Parental mental illness is associated with profound reductions in educational attainment in the subsequent generation, with children from dual-affected families at uniquely high risk.

## Introduction

Education is one of the strongest predictors of future life quality, linked directly to not only an individual's economic success, but also their social engagement, employment stability, satisfaction, physical health, and life expectancy (Autor, 2014; Baum & Payea, 2005; Marmot & Bell, 2009; Masters, Link, & Phelan, 2015; Patton et al., 2016). From an economic perspective, a more educated workforce is more productive (Hoenack, 1993), with investments in education associated with global improvements to living standards, market standings, and civic investment (Leslie & Brinkman, 1988; Patton et al., 2016; Weisbrod, 1962). However, despite these evident benefits – and attendant global efforts to improve educational attainment – high rates of school drop-out persist across much of the developed world (Eurostat, 2020; McFarland et al., 2019; Organisation for Economic Co-operation and Development, 2016).

The role of family in setting expectations for academic engagement, as well as providing the environment conducive to their fulfillment, is well-documented; skills that will form the basis for scholastic and occupational success, including cognitive and noncognitive features [e.g. motivation; (Heckman, 2006)], begin to take shape early in life and have been shown to impact directly on the preparedness of children as they enter school (Denton & West, 2002; Morrison, Bachman, & Connor, 2005). Familial risk factors such as low level of parental income, education, or health have the potential to limit such skill development (Burchinal, Roberts, Zeisel, Hennon, & Hooper, 2006; Gutman, Sameroff, & Cole, 2003; Luster & McAdoo, 1994; Raver, 2004) with research showing that children facing either more, or simply more long-term, risk factors have a higher rate of later school failure (Huffman, Mehlinger, & Kerivan, 2000; Laucht, Esser, & Schmidt, 1997; Rutter, 1979; Wachs, 2000). Beyond reflecting mere environment, the genetic contribution established for many such features – including socio-economic status (Hill et al., 2016; Trzaskowski et al., 2014), educational attainment (Ayorech, Krapohl, Plomin, & von Stumm, 2017; Krapohl & Plomin, 2016; Lee et al., 2020), and mental/physical health (Hagenaars et al., 2016; Martin, Taylor, & Lichtenstein, 2018) – suggest that the evolution of such familial risk factors, as well as a child's propensity to surmount them, may be genetically influenced.

As a set of heritable conditions which often present or persist with chronic symptomatology in early adulthood (thus intersecting the prime child-bearing/rearing years) and are, themselves, associated with profound impacts on life quality and opportunity [e.g. (Breslau, Lane, Sampson, & Kessler, 2008; Evans, Banerjee, Leese, & Huxley, 2007)], psychiatric disorders have long been considered a meaningful, long-term risk factor in the context of educational achievement (Perez-Vigil et al., 2018a, 2018b; Vilaplana-Perez et al., 2020a, 2021). Where parents are affected, the bearing of symptoms on children's cognitive and emotional development has, in particular, been a topic of investigation over the past century, with questions raised about transgenerational impacts on key features including educational attainment (Ranning et al., 2018).

Due to complex causes, including societal conventions regarding mothers as primary care-givers, much of the research in this area has focused on maternal mental health (Ramchandani & Psychogiou, 2009). This is particularly true of work in early-childhood development, where investigations have established a link, for instance, between maternal depression in the pre-natal/post-natal periods and children's cognitive delays (Murray et al., 2011; Murray & Cooper, 2003), teacher-rated social and behavioral difficulties (Goodman, Brogan, Lynch, & Fielding, 1993; Goodman, Lamping, & Ploubidis, 2010) and later mental health issues. Work considering the relationship of paternal mental health to such features has been slower to emerge. However, given the equal genetic contribution of fathers to their children, and growing acknowledgement of the influence all parents exert on normal child development, the past two decades have seen some investment in paternally-focused investigations. As with mothers, work in fathers has focused primarily on depression, with findings suggesting comparable effects on early childhood emotional and behavioral adjustment (Beardslee, Versage, & Gladstone, 1998; Kane & Garber, 2004; LeFrancois, 2012; Rominov, Giallo, & Whelan, 2016). Select differences, including greater behavioral problems among the male children of depressed fathers, have also been noted (Ramchandani & Psychogiou, 2009; Ramchandani, Stein, Evans, O'Connor, & Alspac study team, 2005), with similar differences observed in the offspring of fathers with substance use disorders (SUD) (Harter, 2000).

The above studies represent a small body of literature, with a focus on depression that neglects the breadth of mental health problems increasingly burdening, and being balanced by, adults with dependents. Though depression is one of the most common disorders worldwide, the global incidence of all major mental health conditions [e.g. anxiety disorders, bipolar disorder, autism spectrum disorder (ASD), among others] continues to grow, directly affecting at least one in four persons over the life course (one in two in the United States) and estimated to account for more than a third of all years lived with disability (Reeves et al., 2011; Vigo, Thornicroft, & Atun, 2016; World Health Organization, 2001). Work considering a broader range of parental mental disorders is therefore critical to capturing the true relationship of these conditions to offspring education: clarifying not only the magnitude, but potentially the differential effects of these diverse disorders. In addition, the focus of extant work on the distinct investigation of mothers *or* fathers – as opposed to mothers *and* fathers *–* fails to reflect findings showing that individuals with psychiatric conditions often partner together (Nordsletten et al., 2016). The offspring of families where both parents have a psychiatric disorder have remarkably high rates of psychiatric disorders themselves (Gottesman, Laursen, Bertelsen, & Mortensen, 2010; Nordsletten et al., 2020), which in turn may have additionally negative impacts on educational attainment, presumably through a combination of both genetic and environmental adversities. Given findings suggesting unique impacts of maternal and paternal psychopathology on offspring development and related outcomes – including academic achievement (Harter, 2000) – understanding whether, and to what degree, the differences manifest additively in dual-affected families presents an important question, as it may help identify a particularly vulnerable group of individuals in need of early support and intervention.

**Fig. 1.**
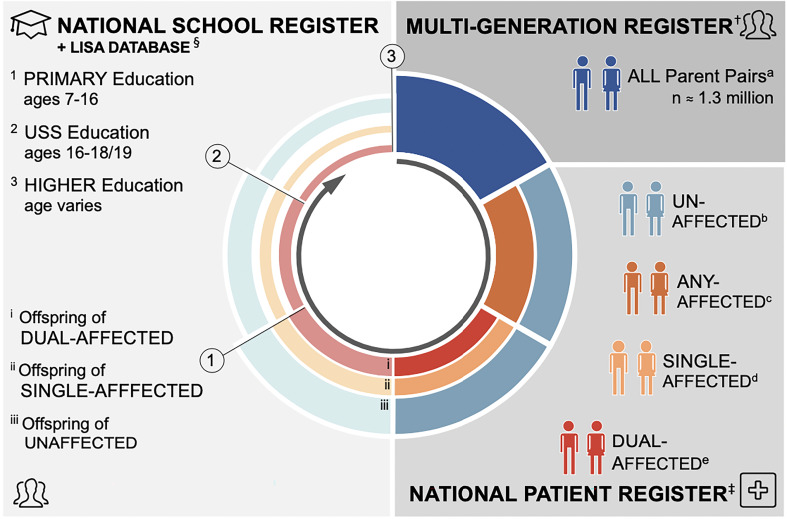
Diagram of study design. ^†^The Multi-Generation Register (MGR) contains detail on the biological parents of all individuals born (since 1932) or registered (since 1961) in Sweden. Our study cohort includes all parents of singleton births occurring between 1 January 1973 and 31 December 1997 – this latter cut-off chosen to allow all children adequate follow-up time for education outcomes (*n* = 1290.157 unique parental pairs; 2 226 451 unique offspring). ^‡^The national patient register (NPR) contains discharge diagnostic information for all inpatient services received in Sweden (since 1969) and all specialist outpatient services (since 2001). Each visit is coded in accordance with the International Classification of Disease [ICD-8 (1969–1986), ICD- 9 (1987–1996), and ICD-10 (1997 + )]. ^§^The National School Register (1988 + ) was used to define compulsory education outcome through capture all study participants graduating municipal and independent schools in Sweden in 1998-2013. The Longitudinal Integration Database for Health Insurance and Labour Market Studies (LISA) records educational attainment for all Swedish residents aged 16 + since 1990, and was used to retrieve information on post-compulsory education outcomes. ^a^All unique parent pairs identified in the MGR. ^b^Exposure group 1: Parent pairs in which neither is affected with a psychiatric disorder of interest. ^c^Parent pairs in which at least one member is affected with a psychiatric disorder of interest. ^d^Exposure group 2: Parent pairs in which only one member is affected with a psychiatric disorder of interest. ^e^Exposure group 3: Parent pairs in which both members are affected with a psychiatric disorder of interest. *^1^*Outcome 1: Complete Primary Education = Completion of Compulsory Education = Eligible to Enter Upper Secondary School (USS). *^2^*Outcome 2a: Finish Upper Secondary School (USS). *^3^*Outcome 3a: Starting University; Outcome 3b: Finish University.

Here, we present results of a study examining the relationship of parental psychopathology to children's educational outcomes. Using data drawn from the Swedish National Registers (see Fig. 1), we have been able, for the first time, to examine these outcomes objectively over the life course (from primary school through to university), both by parental diagnosis (e.g. schizophrenia *v.* major depression) and the diagnostic structure of parental pairs (e.g. single *v.* dual-affected; within *v.* across disorder). Leveraging these unique resources, we tested the hypotheses that (1) educational achievement among the offspring of parents with mental illness would be impaired relative to offspring of unaffected parents, and, (2) the impairment would be most pronounced among the offspring of dual-affected parents, regardless of whether these parents shared or differed in their respective diagnoses.

## Materials and methods

### Ethics

Ethical approval was obtained from the Regional Ethics Review Board in Stockholm, Sweden (Protocol Number: 2013/862–31/5). The requirement for informed consent was waived because the study was register-based and all included individuals deidentified.

### Swedish national registers

We assembled a population-based birth cohort (Fig. 1) by linking data, via unique national identification numbers (Ludvigsson, Otterblad-Olausson, Pettersson, & Ekbom, 2009), across the following Swedish registers:

The Multi-Generation Register (MGR) (Ekbom, 2011); The National Patient Register (NPR) (Ludvigsson et al., 2011); The Cause of Death Register (Brooke et al., 2017); The Prescribed Drug Register (PDR) (Wettermark et al., 2007); The Total Population Register (TPR) (Ludvigsson et al., 2016); the Longitudinal Integration Database for Health Insurance and Labour Market Studies (LISA) (Ludvigsson, Svedberg, Olen, Bruze, & Neovius, 2019); and The National School Register (‘The Swedish National Agency for Education. National School Register. Published 2018. Available from: https://www.skolverket.se/.’). Data extracted from each register are detailed in the online Supplementary (Note 1).

### Study population and sub-cohorts

The study cohort consisted of all singletons (e.g. excluding twins) born in Sweden (1 January 1973 – 31 December 1997), for whom two biological parents were registered in MGR. In line with prior studies (Niederkrotenthaler et al., 2014; Perez-Vigil et al., 2018a, 2018b; Vilaplana-Perez et al., 2020a, 2021), exclusion criteria included: death/emigration from Sweden before age 15 (i.e. conclusion of compulsory school); presence of an organic brain disorder [the International Classification of Disease, Tenth edition (ICD-10): F00–F09] or global learning disability (ICD-10: F70–F79); birthplace of both parents foreign/unidentifiable; and adoption. Following these exclusions (see online SupplementaryFig. S1a), the final cohort yielded 2 226 451 index offspring (48.7% women) from 1 290 157 unique parental pairs. These index offspring were followed until 31 December 2013.

For each educational outcome, separate sub-cohorts of index offspring were created, capturing individuals with (1) the minimum person-time necessary to achieve the corresponding outcome (online SupplementaryFig. S1a & b) and (2) comparable definitions of the target outcomes. All age cut-offs were drawn from reporting on the Swedish educational system (Deen, 2007; Halldén, 2008), with resulting sub-cohorts as follows: eligibility to access upper secondary school (USS) (age 15–16; *n* = 1 416 867), finishing USS (age 19 + , *n* = 1 985 519), starting University (age 21 + , *n* = 1 781 145), and finishing University (age 25, *n* = 1 344 192).

### Exposures: parental psychopathology

We retrieved data from the NPR for select, parental lifetime psychiatric diagnoses. Diagnoses of interest were chosen to capture a broad range of diagnostic categories, while also remaining confined to those with well-validated protocols for NPR identification (Dalman, Broms, Cullberg, & Allebeck, 2002; Ekholm et al., 2005; Idring et al., 2012; Ludvigsson et al., 2011; Rück et al., 2015; Sellgren, Landen, Lichtenstein, Hultman, & Langstrom, 2011; Vilaplana-Perez et al., 2020b), and aduate power (e.g. population prevalence; sex-distribution) for planned analyses. Ultimately, we selected ICD-8/-9/-10 codes identifying the following 11 conditions (see online Supplementary Table S1a): attention-deficit/hyperactivity disorder (ADHD) (Larsson et al., 2013), ASD (Idring et al., 2012; Power et al., 2013), schizophrenia (Lichtenstein et al., 2006; Power et al., 2013), bipolar disorder (Power et al., 2013; Sellgren et al., 2011), major depressive disorder (MDD) (Power et al., 2013), generalized anxiety disorder (GAD), agoraphobia (Li, Sundquist, & Sundquist, 2011), social phobia (Vilaplana-Perez et al., 2020b), obsessive-compulsive disorder (OCD) (Rück et al., 2015), SUD (Power et al., 2013), and Tourette's disorder and chronic tic disorders (TD/CTD) (Rück et al., 2015). For ADHD, a validated algorithm capturing lifetime dispensation of ADHD medication (via the PDR) was used to complement the ICD-codes, improving case capture (Larsson et al., 2013). To avoid diagnostic misclassification, we applied a minimum age of diagnosis to each of the selected disorders.

To explore all diagnostic combinations, three groups of exposure variables were constructed (see online Supplementary Figs S2, S3 for visualization):

1. *Parent*(*s*) *Affected with Any Disorder of Interest*: Pairs in which ⩾ 1 member has a disorders of interest. These pairings included (i) dual-affected pairs (i.e. both parents affected; pairs permitted to share or differ in their respective diagnoses), and (ii) single-affected pairs (i.e. only one parent affected) (online Supplementary Fig. S2a).
2. *Parent*(*s*) *Affected with a Disorder-Group of Interest*: To maximize power, we clustered the disorders of interest into five operational groups: (1) neuropsychiatric disorders (ADHD, ASD, and TD/CTD), (2) SUD, (3) GAD and MDD, (4) agoraphobia, social phobia, and OCD, and (5) schizophrenia and bipolar disorder. Within each disorder group, exposure variables were generated– as above – to distinguish dual-affected and single-affected pairs [e.g. ‘dual-affected’ with neuropsychiatric disorders would indicate both members have ADHD, ASD, or TD/CTD (in any combination)] (online Supplementary Fig. S2b).
3. *Parent*(*s*) *Affected with a Specific Disorder of Interest*: Exposure groups were also generated for each of the eleven disorders of interest. For each, we identified all (i) within-disorder dual-affected pairs (e.g. mother and father with OCD), (ii) cross-disorder dual-affected pairs (e.g. mother with OCD, father with MDD), and (iii) single-affected pairs (e.g. mother with OCD, father without OCD) (online Supplementary Fig. S3).

Comparison (General Population) pairs were selected using an ‘uncleaned’ technique (Gottesman et al., 2010). This approach imposes no restriction on parental diagnoses and represents, for each of the above groupings, the remainder of the full study population (see online Supplementary Note 3 for details). This method retains and reflects the high rates of comorbidity inherent to psychiatric populations and ensures real-world, conservative measures of associations.

As parents may be diagnosed with multiple disorders, parental pairs were permitted to appear across exposure groups. The same individual may, further, appear as both offspring and parent if he/she was both born and had a child during the study period.

### Outcomes: offspring educational attainment

An overview of the Swedish school system (including compulsory and post-compulsory education) is provided in the online Supplementary (see Note 2). Key outcomes in the current study (Fig. 1) included: (1) finishing compulsory school/eligibility to access USS, (2) finishing USS, (3) starting university studies, and (4) finishing university. Eligibility to access USS was defined for all index offspring who graduated from compulsory school 1998–2013, with the graduation range defined due to the equivalence of eligibility criteria used in this period. Eligibility was defined based on passing grades, obtained from the National School Register, which are required for accessing a vocational program (i.e. the lowest requirement to access USS), and dichotomized as eligible *v.* non-eligible. Information on post-compulsory education was derived from the LISA database for all index offspring and included records on: finishing USS, starting a university education, and finishing a university education (each variable dichotomized as achieved *v.* not achieved).

### Covariates

Data on the psychiatric diagnoses of each offspring were derived from the NPR, mirroring procedures used in the parents. Gender and year of birth, for all cohort members, were drawn from the TPR. Family identification numbers were created via the MGR, to link all offspring with shared parents. For analyses utilizing parental education information, information regarding maternal and paternal education was collected from LISA and categorised as finishing compulsory school (reference), incomplete USS, finishing USS, starting unspecified postsecondary education (not at university), starting university education, finishing unspecified postsecondary education (not at university), finishing university education, postgraduate education (licentiate or doctoral degree), unclear, or missing. Within each family, the highest educational level among parents was then defined and used to define parental eduction. If one parent was missing such information, educational level of the other parent represented the educational level for the pair.

### Statistical analysis

Separately for each education outcome, logistic regression models were fitted within each corresponding sub-cohort of the index offspring to compute odds ratio (OR) and 95% confidence interval (95% CI) for each of the following exposure groups, relative to the offspring on the general population pairs: offspring of parents dual-affected by any of the eleven major psychiatric disorders, offspring of parents single-affected by any of the eleven disorders, offspring of parents dual-affected by each of the five group of disorders (one group at the time), offspring of parents single-affected by each of the five group of disorders (one group at the time), offspring of parents within-disorder dual-affected by each specific disorder (one disorder at the time), offspring of parents cross-disorder dual-affected by specific disorders (separately for each cross-disorder combination), and offspring of parents single-affected by specific disorder (one disorder at the time). Crude and adjusted models were applied with the latter being controlled for offspring gender and year of birth (5-year categories from 1973), and maternal and paternal year of birth (categorized as decades from birth: <1940, 1950s, 1960s, 1970s, ⩾1970).

To explore the role of offspring own psychopathology on associations of interest, we repeated all analyses after excluding offspring diagnosed with any of the eleven psychiatric disorders. In addition, we also conducted analyses to assess the role of parental education level on the association under study. Two analyses were performed: (1) an additional covariate was introduced to the fully-adjusted model, reflecting the highest level of education of the parental pair, and (2) a mediation analysis was executed to estimate the proportion of the total effect explained by differences in parental education.

All analyses were clustered by family identification number and employed a robust sandwich estimator of standard errors to account for non-independence between repeated observations within families (StataCorp, 2017; Williams, 2000).

### Sensitivity analyses

Main analyses were repeated using a more stringent comparison group, i.e., a ‘cleaned population’ (Gottesman et al., 2010) in which neither parent had a lifetime diagnosis for any of the eleven disorders of interest. In addition, we conducted an analysis which employed a more conservative definition of parental schizophrenia and bipolar disorder (Ripke et al., 2013), requiring ⩾2 discharge diagnoses. Next, we restricted each sub-cohort to individuals whose parents were residents of Sweden in 1997 or later (i.e. if parents neither died, nor emigrated, prior 1997) to ensure the completeness of data on parental diagnoses specific to ICD-10 (implemented in 1997). All abovementioned sensitivity analyses were performed within sub-cohorts inclusive of all index offspring.

Given the multiple prespecified analyses, all tests employed two-tailed significance set at a corrected level of *p* < 0.001. Data management was performed using SAS, version 9.4 (SAS Institute, Cary, NC, USA) and analyses were performed using STATA, version 17.1 (StataCorp LLC, College Station, TX, USA). Mediation was conducted using *ldecomp* command in Stata, to allow decomposing of total effects in logistic regression into direct and indirect effects. As the study protocol was not preregistered, all analyses and their attendant results should be considered exploratory. The study results were reported according to the RECORD guideline and checklist (online Supplementary Table S1b) (Benchimol et al., 2015).

## Results

### Impacts on compulsory (primary) education

**Fig. 2.**
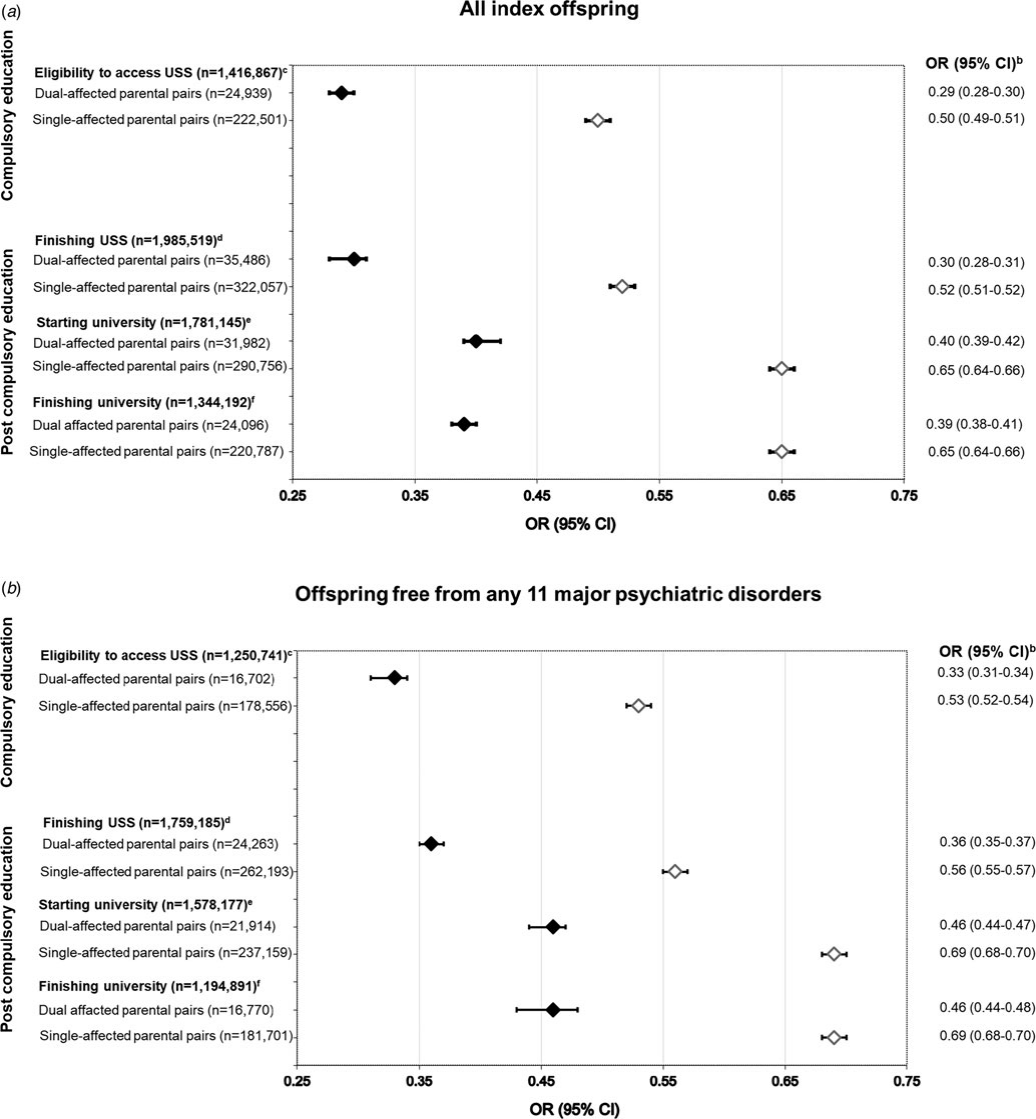
Adjusted odds ratios (OR) and corresponding 95% confidence intervals (CI) for achieving all educational outcomes in all index offspring (panel A) and offspring free from the eleven psychiatric disorders (panel B) with parents dual- and single-affected by any of the eleven psychiatric disorders, compared with individuals with parents from the general population (uncleaned population^a^). Abbreviation: USS, upper secondary school. ^a^‘Uncleaned population’ imposes no restriction on parental diagnoses in the comparison group and represents, in each relevant analysis, the rest of study population. This approach reflects the high rates of psychiatric comorbidity and ensures obtaining real-world, conservative measures of associations. ^b^Adjusted for offspring year of birth (5-year categories starting from 1973), offspring sex, and maternal and paternal year of birth in decades (<1940, 1950s, 1960s, 1970s, ⩾1970). ^c^Individuals who graduated from compulsory school in 1998–2013. The graduation years were chosen due to the different eligibility criteria used prior to 1998. ^d^Individuals born in 1973–1994 (i.e. aged 19 + by the end of follow-up) and who was alive and living in Sweden at age 19 years. ^e^Individuals born in 1973–1992 (i.e. aged 21 + by the end of follow-up) and who was alive and living in Sweden at age 21 years. ^f^Individuals born in 1973–1988 (i.e. aged 25 + by the end of follow-up) and who was alive and living in Sweden at age 25 years. *Note*: All models are clustered by family identification number with robust standard error estimation (sandwich estimator). For all OR (95% CI), *p* values are <0.001. ‘*Dual-affected pairs*’ imply that both parents have a record of at least one of the eleven disorders (being diagnosed with either the same disorder or with different ones), and ‘*single-affected pairs*’ imply that one parent has a record of any such disorders, the other is free from any of 11 disorders.

Overall, compared to the offspring of unaffected pairs, individuals who had one parent affected with any of the eleven psychiatric conditions considered were found to be half as likely to access USS [adjusted OR (aOR) 0.50; 95% CI 0.49–0.51; *p* < 0.001]. This relationship was substantially increased among offspring with two affected parents (aOR 0.29; 95% CI 0.28–0.30; *p* < 0.001) (Fig. 2a). The pattern was repeated in a sub-analysis by diagnostic group (e.g. Neuropsychiatric Disorders), which indicated markedly lower odds of accessing USS in the offspring of single-affected (aORs range: 0.39–0.61; all *p* values < 0.001) and, again to a higher degree, dual-affected (aORs range: 0.24–0.40; all *p* values < 0.001) pairs (Table 1). The substantial variation in achievement by group - emergent in single-affected offspring (e.g. odds of accessing USS were more significantly reduced when a parent had a neuropsychiatric condition; see Table 1) – was narrowed for the offspring of dual-affected pairs.

**Table 1.** Odds ratio (OR) and corresponding 95% confidence intervals (CI) for achieving compulsory and post compulsory educational outcomes in individuals with parents dual- or single- affected by five groups of psychiatric disorders, compared with individuals with parents from the general population (uncleaned population^a^)

	Dual-affected parental pairs				Single-affected parental pairs			
	Offspring of affected pairs	Offspring of unaffected pairs	Crude model	Fully-adjusted^b^	Offspring of affected pairs	Offspring of unaffected pairs	Crude model	Fully-adjusted^b^
	*n* (%)	*n* (%)	OR (95% CI)	OR (95% CI)	*n* (%)	*n* (%)	OR (95% CI)	OR (95% CI)
*Compulsory School*^c^								
Neuropsychiatric disorders^d^	*n* = 607	*n* = 1 416 260			*n* = 21 727	*n* = 1 395 140		
Achieved	439 (72.32)	1 292 946 (91.29)	0.25 (0.20–0.30)*	0.30 (0.25–0.37)*	17 086 (78.64)	1 276 299 (91.48)	0.34 (0.33–0.35)*	0.39 (0.37–0.40)*
SUD	*n* = 10 518	*n* = 1 406 349			*n* = 121 273	*n* = 1 295 594		
Achieved	7523 (71.53)	1 285 862 (91.43)	0.23 (0.22–0.25)*	0.24 (0.23–0.25)*	99 839 (82.33)	1 193 546 (92.12)	0.40 (0.39–0.41)*	0.41 (0.40–0.42)*
GAD and MDD	*n* = 6291	*n* = 1 410 576			*n* = 135 587	*n* = 1 281 280		
Achieved	4973 (79.05)	1 288 412 (91.34)	0.36 (0.33–0.38)*	0.38 (0.36–0.41)*	116 416 (85.86)	1 176 969 (91.86)	0.54 (0.53–0.55)*	0.56 (0.55–0.57)*
AGO, SOC, and OCD	*n* = 189	*n* = 1 416 678			*n* = 19 446	*n* = 1 397 421		
Achieved	128 (67.72)	1 293 257 (91.29)	0.20 (0.14–0.28)*	0.24 (0.17–0.34)*	16 029 (82.43)	1 277 356 (91.41)	0.44 (0.42–0.46)*	0.48 (0.46–0.50)*
SCZ and BIP	*n* = 475	*n* = 1 416 392			*n* = 32 416	*n* = 1 384 451		
Achieved	384 (80.84)	1 293 001 (91.29)	0.40 (0.31–0.52)*	0.40 (0.31–0.52)*	27 978 (86.31)	1 265 407 (91.40)	0.59 (0.57–0.61)*	0.61 (0.59–0.63)*
*Finishing USS*^e^								
Neuropsychiatric disorders^d^	*n* = 498	*n* = 1 985 021			*n* = 21 146	*n* = 1 964 373		
Achieved	185 (37.15)	1 599 725 (80.59)	0.14 (0.12–0.17)*	0.19 (0.16–0.24)*	11 828 (55.87)	1 588 082 (80.84)	0.30 (0.29–0.31)*	0.36 (0.35–0.37)*
SUD	*n* = 16 344	*n* = 1 969 739			*n* = 188 577	*n* = 1 796 942		
Achieved	8171 (49.99)	1 591 739 (80.83)	0.24 (0.23–0.24)*	0.24 (0.23–0.25)*	124 865 (66.21)	1 475 045 (82.09)	0.43 (0.42–0.43)*	0.42 (0.42–0.43)*
GAD and MDD	*n* = 7723	*n* = 1 977 796			*n* = 181 143	*n* = 1 804 143		
Achieved	4713 (61.03)	1 595 197 (80.66)	0.37 (0.36–0.39)*	0.41 (0.39–0.43)*	129 082 (71.17)	1 470 828 (81.53)	0.56 (0.55–0.57)*	0.58 (0.57–0.59)*
AGO, SOC, and OCD	*n* = 194	*n* = 1 985 325			*n* = 22 334	*n* = 1 963 185		
Achieved	92 (47.42)	1 599 818 (80.58)	0.22 (0.16–0.30)*	0.29 (0.21–0.39)*	14 487 (64.87)	1 585 423 (80.76)	0.44 (0.43–0.45)*	0.49 (0.48–0.51)*
SCZ and BIP	*n* = 674	*n* = 1 984 845			*n* = 46 253	*n* = 1 939 266		
Achieved	417 (61.87)	1 599 493 (80.59)	0.39 (0.33–0.46)*	0.40 (0.34–0.47)*	32 766 (70.84)	1 567 144 (80.81)	0.58 (0.56–0.59)*	0.58 (0.57–0.60)*
*Starting university*^f^								
Neuropsychiatric disorders^d^	*n* = 370	*n* = 1 780 775			*n* = 17 235	*n* = 1 763 910		
Achieved	42 (11.35)	684 248 (38.42)	0.20 (0.15–0.29)*	0.34 (0.24–0.49)*	3747 (21.74)	680 543 (38.58)	0.44 (0.42–0.46)*	0.58 (0.56–0.61)*
SUD	*n* = 14 998	*n* = 1 766 147			*n* = 172 571	*n* = 1 608 574		
Achieved	2254 (15.03)	682 036 (38.62)	0.28 (0.27–0.29)*	0.28 (0.26–0.29)*	43 800 (25.38)	640 490 (39.82)	0.51 (0.50–0.52)*	0.51 (0.50–0.52)*
GAD and MDD	*n* = 6720	*n* = 1 774 425			*n* = 161 369	*n* = 1 619 776		
Achieved	1763 (26.24)	682 527 (38.46)	0.57 (0.53–0.60)*	0.62 (0.58–0.66)*	51 234 (31.75)	633 056 (39.08)	0.72 (0.71–0.73)*	0.76 (0.75–0.77)*
AGO, SOC, and OCD	*n* = 161	*n* = 1 780 984			*n* = 19 305	*n* = 1 761 840		
Achieved	32 (19.88)	684 258 (38.42)	0.40 (0.26–0.61)*	0.58 (0.37–0.91)^#^	5077 (26.30)	679 213 (38.55)	0.57 (0.55–0.59)*	0.66 (0.64–0.69)*
SCZ and BIP	*n* = 604	*n* = 1 780 541			*n* = 41 353	*n* = 1 739 488		
Achieved	188 (31.13)	684 102 (38.42)	0.72 (0.60–0.87)^§^	0.73 (0.61–0.88)^§^	14 353 (34.46)	669 937 (38.51)	0.84 (0.82–0.86)*	0.85 (0.83–0.87)*
*Finishing University*^g^								
Neuropsychiatric disorders^d^	*n* = 165	*n* = 1 344 027			*n* = 10 103	*n* = 1 334 089		
Achieved	10 (6.06)	348 993 (25.97)	0.18 (0.10–0.35)*	0.32 (0.17–0.62)^§^	1353 (13.39)	347 650 (26.06)	0.44 (0.41–0.47)*	0.59 (0.55–0.63)*
SUD	*n* = 11 704	*n* = 1 332 488			*n* = 134 529	*n* = 1 209 663		
Achieved	969 (8.28)	348 034 (26.12)	0.25 (0.24–0.27)*	0.26 (0.24–0.28)*	21 324 (15.85)	327 679 (27.09)	0.51 (0.50–0.52)*	0.51 (0.50–0.52)*
GAD and MDD	*n* = 4709	*n* = 1 339 483			*n* = 118 284	*n* = 1 225 908		
Achieved	808 (17.16)	348 195 (25.99)	0.59 (0.54–0.64)*	0.64 (0.59–0.70)*	24 927 (21.07)	324 076 (26.44)	0.74 (0.73–0.75)*	0.78 (0.76–0.79)*
AGO, SOC, and OCD	*n* = 98	*n* = 1 344 094			*n* = 12 992	*n* = 1 331 200		
Achieved	10 (10.20)	348 993 (25.96)	0.32 (0.17–0.61)*	0.46 (0.23–0.90)^#^	2216 (17.06)	346 787 (26.05)	0.58 (0.55–0.61)*	0.68 (0.64–0.71)*
SCZ and BIP	*n* = 440	*n* = 1 343 752			*n* = 31 360	*n* = 1 312 832		
Achieved	77 (17.50)	348 926 (25.97)	0.60 (0.47–0.78)*	0.63 (0.48–0.81)*	7177 (22.89)	341 826 (26.04)	0.84 (0.82–0.87)*	0.85 (0.83–0.88)*

*Abbreviation*: AGO, agoraphobia; BIP, bipolar disorder; GAD, generalized anxiety disorder; MDD, major depressive disorder; OCD, obsessive-compulsive disorder; SCZ, schizophrenia; SOC, social phobia; SUD, substance use disorders; USS, upper secondary school.

a ‘Uncleaned population’ imposes no restriction on parental diagnoses in the comparison group and represents, in each relevant analysis, the rest of study population. This approach reflects the high rates of psychiatric comorbidity and ensures obtaining real-world, conservative measures of associations.

b Adjusted for offspring year of birth (5-year categories starting from 1973), offspring sex, and maternal and paternal year of birth in decades (<1940, 1950s, 1960s, 1970s, ⩾1970).

c Individuals who graduated from compulsory school in 1998–2013 (*n* = 1 416 867). The graduation years were chosen due to the different eligibility criteria used prior to 1998.

d Neuropsychiatric disorder group includes attention-deficit hyperactivity disorder, ASD, and TD/CTD.

e Individuals born in 1973-1994 (i.e. aged ⩾19 by the end of follow-up) and who was alive and living in Sweden at age 19 years (*n* = 1 985 519).

f Individuals born in 1973–1992 (i.e. aged ⩾21 by the end of follow-up) and who was alive and living in Sweden at age 21 years (*n* = 1 781 145).

g Individuals born in 1973–1988 (i.e. aged ⩾25 by the end of follow-up) and who was alive and living in Sweden at age 25 years (*n* = 1 344 192).

^#^*p* value < 0.05; ^§^ *p* value < 0.01; * *p* value < 0.001.

*Note*: All models are clustered by family identification number with robust standard error estimation (sandwich estimator). Within each group, *‘dual-affected parental pairs’* imply that both parents have records of the disorders from the group (both parents diagnosed with either the same or different disorders within the group; for example, attention-deficit hyperactivity disorder, or ASD, or TD/CTD in the analysis of neuropsychiatric disorders). Within each group, *‘single-affected parental pairs’* imply that one parent has at least one disorder from the group and the other parent is free from any disorders from the corresponding group. For each educational outcome, separate subcohorts of index offspring were created, comprising the individuals who had the time necessary to achieve the corresponding outcome.

**Fig. 3.**
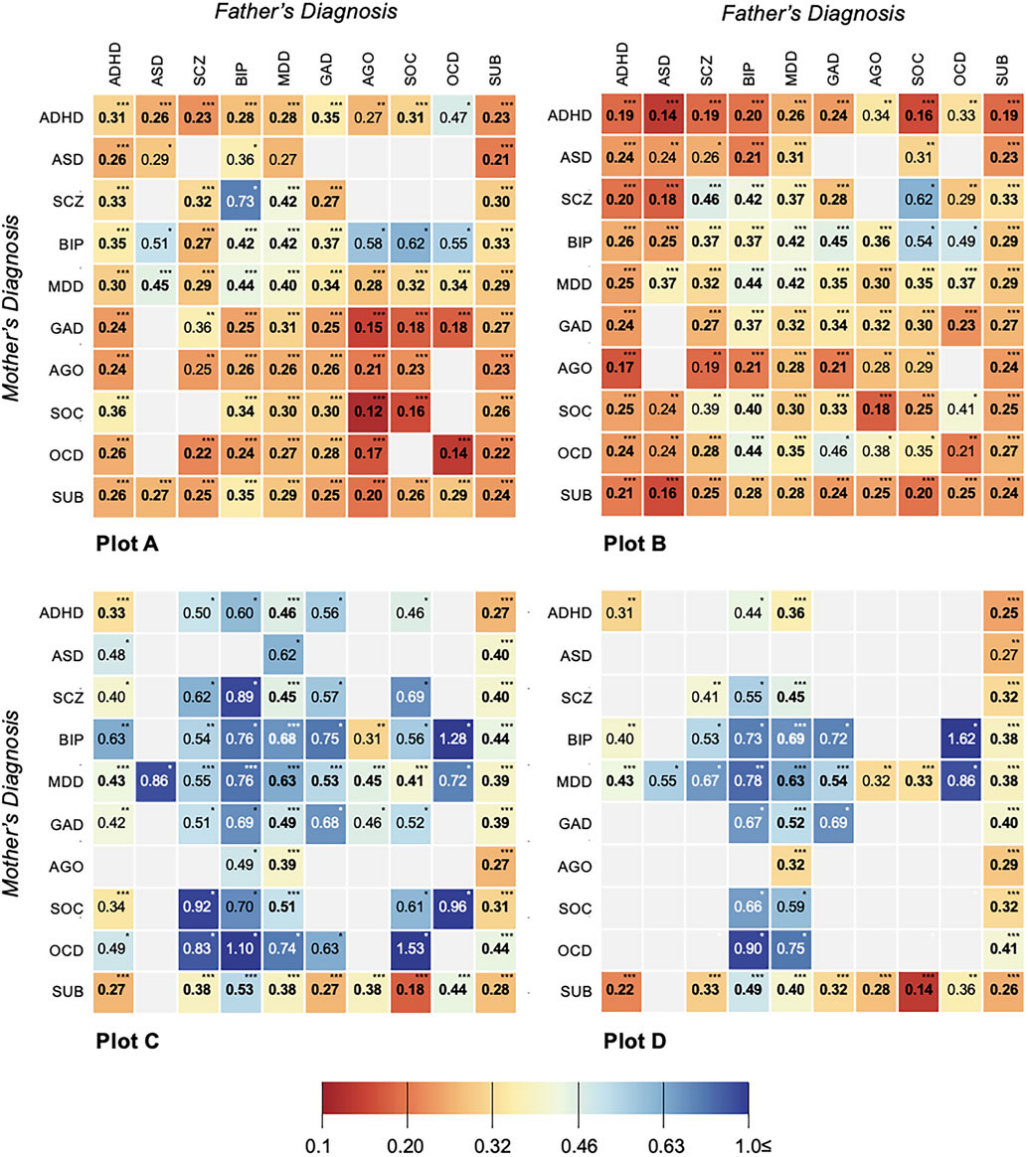
Adjusted odds ratios (OR) and *p* values (***p value < 0.001; **p value < 0.01; *p value < 0.05) for achieving compulsory educational outcome (Plot A) and post-compulsory educational outcomes [Plot B (post-secondary)-D (finishing university)] in individuals with parents affected within-disorder and cross-disorder, compared with individuals with parents from the general population (uncleaned population^a^). *Abbreviations:* ADHD, attention-deficit hyperactivity disorder; AGO, agoraphobia; ASD, autism spectrum disorder; BIP, bipolar disorder; GAD, generalized anxiety disorder; MDD, major depressive disorder; OCD, obsessive-compulsive disorder; SCZ, schizophrenia; SOC, social phobia; SUD, substance use disorders. ^a^‘Uncleaned population’ imposes no restriction on parental diagnoses in the comparison group and represents, in each relevant analysis, the rest of study population. This approach reflects the high rates of psychiatric comorbidity and ensures obtaining real-world, conservative measures of associations. *Note*: All models are adjusted for offspring year of birth (5-year categories starting from 1973), offspring sex, and maternal and paternal year of birth in decades (<1940, 1950s, 1960s, 1970s, ⩾1970) and clustered by family identification number with robust standard error estimation (sandwich estimator). ‘*Within-disorder affected parental pairs’* imply that both parents have records of the same disorder. ‘*Cross-disorder affected parental pairs’* imply that parents have records of different disorders out of 11 psychiatric disorders in question. For each educational outcome, separate subcohorts of index offspring were created, comprising the individuals who had the time necessary to achieve the corresponding outcome. Empty cells indicate the results of analyses with no exposed individuals or if ⩽5 exposed cases or exposed non-cases were available.

Analyses by individual parental diagnosis (e.g. ADHD) showed more nuanced variation in outcomes for dual-affected offspring (see Fig. 3a), though the majority of significant findings fell in a range similar to grouped analyses (e.g. aOR range: 0.22–0.43). Exceptions included having a mother with SUD and father with agoraphobia (aOR 0.20; 95% CI 0.15–0.28; *p* < 0.001), mother with social phobia and father with agoraphobia (aOR 0.12; 95% CI 0.05–0.30; *p* < 0.001), or both parents with OCD (aOR = 0.14; 95% CI 0.05–0.34; *p* value < 0.001). Fluctuation was sustained among single-affected samples (online Supplementary Table S2, ‘all offspring’), with the offspring of an ADHD-affected parent, for example, significantly less likely to achieve USS eligibility (aOR 0.38; 95% CI 0.37–0.40; *p* < 0.001) than a child whose parent had bipolar disorder (aOR 0.64; 95% CI 0.61–0.66; *p* < 0.001). For all disorder-specific comparisons, children of dual-affected pairs showed lower odds of USS eligibility relative unaffected counterparts, exceeding differences observed among single-affected families.

### Impacts on post-compulsory education: completing USS, starting/completing university

Across post-compulsory outcomes, offspring with one or more affected parents experienced significant reductions in their odds of achievement (Fig. 2a). The ORs for finishing USS, starting university, and finishing university were lower across analyses considering each diagnostic group (Table 1), each individual parental disorder (online Supplementary Table S2; see ‘all offspring’), as well as an analyses capturing within- and cross-disorder pairings (Fig. 3b–d). Consistent with compulsory outcomes, the reductions in likelihood of post-compulsory achievement were doubled where both parents were affected, regardless of parental diagnosis.

### The role of offspring mental health

To isolate the impact of offspring's own mental health, all analyses were repeated in a sub-cohort restricted to offspring free from any of the examined disorders. The resulting ORs, while slightly attenuated, remained significant across all outcomes [Fig. 2b, online Supplementary Fig. S4, Table 2, online Supplementary Table S2 (see offspring free from 11 psychiatric disorders)], replicating the patterns observed in the main analyses.

**Table 2.** Odds ratio (OR) and corresponding 95% confidence intervals (CI) for achieving compulsory and post compulsory educational outcomes *in individuals free from any 11 psychiatric disorders* with parents dual- or single- affected by five groups of psychiatric disorders (one group at the time), compared to individuals with parents from the general population (uncleaned population^a^)

	Dual-affected parental pairs				Single-affected parental pairs				
	Offspring of affected pairs	Offspring of unaffected pairs	Crude model	Fully-adjusted^b^	Offspring of affected pairs	Offspring of unaffected pairs	Crude model	Fully-adjusted^b^
	*n* (%)	*n* (%)	OR (95% CI)	OR (95% CI)	*n* (%)	*n* (%)	OR (95% CI)		OR (95% CI)
*Compulsory School*^c^									
Neuropsychiatric disorders	*n* = 242	*n* = 1 250 499			*n* = 13 737	*n* = 1 237 004			
Achieved	206 (85.12)	1 162 827 (92.99)	0.43 (0.30–0.62)*	0.52 (0.36–0.75)*	11 731 (85.37)	1 151 302 (93.07)	0.43 (0.41–0.46)*		0.49 (0.46–0.51)*
SUD	*n* = 6925	*n* = 1 243 816			*n* = 95 934	*n* = 1 154 807			
Achieved	5393 (77.88)	1 157 640 (93.07)	0.26 (0.25–0.28)*	0.27 (0.25–0.29)*	82 293 (85.78)	1 080 740 (93.59)	0.41 (0.40–0.42)*		0.43 (0.42–0.44)*
GAD and MDD	*n* = 4206	*n* = 1 246 535			*n* = 106 948	*n* = 1 143 793			
Achieved	3562 (84.69)	1 159 471 (93.02)	0.41 (0.38–0.45)*	0.44 (0.40–0.48)*	95 392 (89.19)	1 067 641 (93.34)	0.59 (0.58–0.60)*		0.61 (0.59–0.62)*
AGO, SOC, and OCD	*n* = 119	*n* = 1 250 622			*n* = 14 823	*n* = 1 235 918			
Achieved	89 (74.79)	1 162 944 (92.99)	0.22 (0.13–0.37)*	0.27 (0.16–0.44)*	12 860 (86.76)	1 150 173 (93.06)	0.49 (0.46–0.51)*		0.53 (0.50–0.55)*
SCZ and BIP	*n* = 292	*n* = 1 250 449			*n* = 24 882	*n* = 1 225 859			
Achieved	243 (83.22)	1 162 790 (92.99)	0.37 (0.27–0.51)*	0.37 (0.27–0.52)*	22 296 (89.61)	1 140 737 (93.06)	0.64 (0.61–0.67)*		0.66 (0.63–0.69)*
*Finishing USS*^d^									
Neuropsychiatric disorders	*n* = 192	*n* = 1 758 993			*n* = 13 596	*n* = 1 745 589			
Achieved	107 (55.73)	1 475 607 (83.89)	0.24 (0.18–0.32)*	0.33 (0.25–0.44)*	9254 (68.06)	1 466 460 (84.01)	0.41 (0.39–0.42)*		0.48 (0.46–0.50)*
SUD	*n* = 10 959	*n* = 1 748 226			*n* = 151 366	*n* = 1 607 819			
Achieved	6580 (60.04)	1 469 134 (84.04)	0.28 (0.27–0.30)*	0.28 (0.27–0.30)*	109 430 (72.29)	1 366 348 (84.98)	0.46 (0.45–0.47)*		0.46 (0.45–0.46)*
GAD and MDD	*n* = 5287	*n* = 1 753 898			*n* = 144 967	*n* = 1 614 218			
Achieved	3744 (70.82)	1 471 970 (83.93)	0.46 (0.44–0.50)*	0.50 (0.47–0.53)*	112 169 (77.38)	1 363 545 (84.47)	0.63 (0.62–0.64)*		0.65 (0.64–0.66)*
AGO, SOC, and OCD	*n* = 119	*n* = 1 759 066			*n* = 17 054	*n* = 1 742 131			
Achieved	73 (61.34)	1 475 641 (83.89)	0.30 (0.20–0.47)*	0.41 (0.27–0.63)*	12 318 (72.23)	1 463 396 (84.00)	0.49 (0.48–0.51)*		0.55 (0.53–0.57)*
SCZ and BIP	*n* = 416	*n* = 1 758 769			*n* = 35 939	*n* = 1 723 246			
Achieved	295 (70.91)	1 475 419 (83.89)	0.47 (0.38–0.58)*	0.47 (0.38–0.59)*	27 867 (77.54)	1 447 847 (84.02)	0.66 (0.64–0.67)*		0.66 (0.65–0.68)*
*Starting University*^e^									
Neuropsychiatric disorders	*n* = 142	*n* = 1 578 035			*n* = 11 248	*n* = 1 566 929			
Achieved	24 (16.90)	633 385 (40.14)	0.30 (0.19–0.48)*	0.49 (0.30–0.80)*	3007 (26.73)	630 402 (40.23)	0.54 (0.52–0.57)*		0.70 (0.66–0.73)*
SUD	*n* = 10 061	*n* = 1 568 116			*n* = 138 689	*n* = 1 439 488			
Achieved	1866 (18.55)	631 543 (40.27)	0.34 (0.32–0.36)*	0.33 (0.31–0.35)*	38 668 (27.88)	594 741 (41.32)	0.55 (0.54–0.56)*		0.54 (0.53–0.55)*
GAD and MDD	*n* = 4613	*n* = 1 573 564			*n* = 129 208	*n* = 1 448 969			
Achieved	1389 (30.11)	632 020 (40.16)	0.64 (0.60–0.69)*	0.68 (0.63–0.73)*	44 776 (34.65)	588 633 (40.62)	0.77 (0.76–0.79)*		0.80 (0.79–0.81)*
AGO, SOC, and OCD	*n* = 99	*n* = 1 578 078			*n* = 14 768	*n* = 1 563 409			
Achieved	22 (22.22)	633 387 (40.14)	0.43 (0.26–0.69)^§^	0.65 (0.39–1.08)	4346 (29.43)	629 063 (40.24)	0.62 (0.59–0.64)*		0.71 (0.69–0.74)*
SCZ and BIP	*n* = 371	*n* = 1 577 806			*n* = 32 395	*n* = 1 545 782			
Achieved	134 (36.12)	633 275 (40.14)	0.84 (0.68–1.04)	0.85 (0.69–1.05)	12 225 (37.74)	621 184 (40.19)	0.90 (0.88–0.92)*		0.91 (0.88–0.93)*
*Finishing University*^f^									
Neuropsychiatric disorders	*n* = 72	*n* = 1 194 819			*n* = 6784	*n* = 1 188 107			
Achieved	7 (9.72)	327 933 (27.45)	0.28 (0.13–0.62)^§^	0.49 (0.22–1.10)	1143 (16.85)	326 797 (27.51)		0.53 (0.50–0.57)*	0.70 (0.65–0.75)*
SUD	*n* = 7932	*n* = 1 186 959			*n* = 109 001	*n* = 1 085 890			
Achieved	854 (10.77)	327 086 (27.56)	0.32 (0.29–0.34)*	0.32 (0.29–0.34)*	19 366 (17.77)	308 574 (28.42)		0.54 (0.53–0.55)*	0.54 (0.53–0.55)*
GAD and MDD	*n* = 3312	*n* = 1 191 579			*n* = 95 760	*n* = 1 099 131			
Achieved	672 (20.29)	327 268 (27.47)	0.67 (0.61–0.74)*	0.72 (0.65–0.79)*	22 426 (23.42)	305 514 (27.80)		0.79 (0.78–0.81)*	0.82 (0.81–0.84)*
AGO, SOC, and OCD	*n* = 59	*n* = 1 194 832			*n* = 9985	*n* = 1 184 906			
Achieved	7 (11.86)	327 933 (27.45)	0.36 (0.16–0.77)^§^	0.52 (0.23–1.19)	1963 (19.66)	325 977 (27.51)		0.64 (0.61–0.68)*	0.74 (0.70–0.78)*
SCZ and BIP	*n* = 267	*n* = 1 194 624			*n* = 24 721	*n* = 1 170 170			
Achieved	53 (19.85)	327 887 (27.45)	0.65 (0.49–0.88)^§^	0.66 (0.49–0.90)^#^	6314 (25.54)	321 626 (27.49)		0.90 (0.88–0.93)*	0.92 (0.89–0.95)*
									

*Abbreviation*: AGO, agoraphobia; BIP, bipolar disorder; GAD, generalized anxiety disorder; MDD, major depressive disorder; OCD, obsessive-compulsive disorder; SCZ, schizophrenia; SOC, social phobia; SUD, substance use disorders; USS, upper secondary school.

a ‘Uncleaned population’ imposes no restriction on parental diagnoses in the comparison group and represents, in each relevant analysis, the rest of study population. This approach reflects the high rates of psychiatric comorbidity and ensures obtaining real-world, conservative measures of associations.

b Adjusted for offspring year of birth (5-year categories starting from 1973), offspring sex, and maternal and paternal year of birth in decades (<1940, 1950s, 1960s, 1970s, ⩾1970).

c Individuals who graduated from compulsory school in 1998–2013 (*n* = 1 250 741). The graduation years were chosen due to the different eligibility criteria used prior to 1998.

d Individuals born in 1973–1994 (i.e. aged ⩾19 by the end of follow-up) and who was alive and living in Sweden at age 19 years (*n* = 1 759 185).

e Individuals born in 1973–1992 (i.e. aged ⩾21 by the end of follow-up) and who was alive and living in Sweden at age 21 years (*n* = 1 578 177).

f Individuals born in 1973–1988 (i.e. aged ⩾25 by the end of follow-up) and who was alive and living in Sweden at age 25 years (*n* = 1 194 891).

^#^*p* value < 0.05; ^§^ *p* value < 0.01; * *p* value < 0.001.

*Note*: All models are clustered by family identification number with robust standard error estimation (sandwich estimator). Neuropsychiatric disorder group includes attention-deficit hyperactivity disorder, ASD, and TD/CTD. Within each group, *‘dual-affected parental pairs’* imply that both parents have records of the disorders from the group (both parents diagnosed with either the same or different disorders within the group; for example, ADHD, or ASD, or TD/CTD in the analysis of neuropsychiatric disorders). Within each group, *‘single-affected parental pairs’* imply that one parent has at least one disorder from the group and the other parent is free from any disorders from the corresponding group. For each educational outcome, separate sub- cohorts of index offspring were created, comprising the individuals who had the time necessary to achieve the corresponding outcome.

### The role of parental education

Analyses considering the mediation relationship of parental education level to main effects indicated significant variation, both by parental diagnostic structure and the level of offspring education considered as outcome (see Table 3; online Supplementary Tables S3–S6). Overall, the main exposure-outcome associations remaining significant across these multi-variate models, with parent education level attenuating, but in the majority not negating, the association between parental mental illness and educational outcomes. Parent education level, for example, explained 13.8–35.8% of a child's likelihood to finish compulsory school; however, only among parents dual-affected with neuropsychiatric disorders did controlling for parental education attenuate the main effect to non-significance (e.g. 44.1% effect mediated; see Table 3 & online Supplementary Table S3). Parental education explained, overall, a greater proportion of effects at the level of initiating University (17.3–49.8% of the main effect); the impact was particularly strong for pairs dual-affected by agoraphobia, social phobia, and/or OCD (e.g. 87.7% mediated) or schizophrenia/bipolar disorders (e.g. 43.6% mediated; see online Supplementary Table S5). In contrast, for completion of USS, while parental education explained a variable proportion (4.1–28.8% for USS), no alteration to the significance of main effects was observed (see online Supplementary Table S4).

**Table 3.** Impact of adjustment and mediation for parental education level on main effects, among offspring with parents dual- or single-affected by five groups of psychiatric disorders (one group at the time), compared to individuals with parents from the general population (uncleaned population^a^)

	Parental affected status	Main fully-adjusted model^b^	Additional adjustment for highest parent education level	Mediation: % Main effect represented by indirect effect
		OR (95% CI), *p* value	OR (95% CI), *p* value	%
Compulsory school^c^				
Any disorder	Dual	0.29 (0.28–0.30), *p* < 0.001	0.39 (0.38–0.40), *p* < 0.001	24.2
	Single	0.50 (0.49–0.51), *p* < 0.001	0.57 (0.56–0.58), *p* < 0.001	19.4
Neuropsychiatric disorders^d^	Dual	0.30 (0.25–0.37), *p* < 0.001	0.40 (0.32–0.49), *p* < 0.001	24.3
	Single	0.39 (0.37–0.40), *p* < 0.001	0.45 (0.43–0.47), *p* < 0.001	18.7
SUD	Dual	0.24 (0.23–0.25), *p* < 0.001	0.36 (0.34–0.38), *p* < 0.001	27.9
	Single	0.41 (0.40–0.42), *p* < 0.001	0.50 (0.49–0.51), *p* < 0.001	23.8
GAD and MDD	Dual	0.38 (0.36–0.41), *p* < 0.001	0.45 (0.42–0.48), *p* < 0.001	19.2
	Single	0.61 (0.59–0.62), *p* < 0.001	0.67 (0.65–0.68), *p* < 0.001	18.9
AGO, SOC, and OCD	Dual	0.24 (0.17–0.34), *p* < 0.001	0.36 (0.25–0.53), *p* < 0.001	31.3
	Single	0.53 (0.50–0.55), *p* < 0.001	0.63 (0.59–0.66), *p* < 0.001	28.5
SCZ and BIP	Dual	0.40 (0.31–0.52), *p* < 0.001	0.48 (0.37–0.62), *p* < 0.001	19.5
	Single	0.66 (0.63–0.69), *p* < 0.001	0.70 (0.67–0.74), *p* < 0.001	15.5
Finishing USS^e^				
Any Disorder	Dual	0.30 (0.28–0.31), *p* < 0.001	0.35 (0.34–0.36), *p* < 0.001	13.6
	Single	0.52 (0.51–0.52), *p* < 0.001	0.55 (0.54–0.56), *p* < 0.001	10.9
Neuropsychiatric disorders	Dual	0.19 (0.16–0.24), *p* < 0.001	0.22 (0.18–0.27), *p* < 0.001	9.1
	Single	0.36 (0.35–0.37), *p* < 0.001	0.39 (0.37–0.40), *p* < 0.001	7.0
SUD	Dual	0.24 (0.23–0.25), *p* < 0.001	0.30 (0.28–0.31), *p* < 0.001	16.0
	Single	0.42 (0.42–0.43), *p* < 0.001	0.48 (0.47–0.48), *p* < 0.001	14.0
GAD and MDD	Dual	0.41 (0.39–0.43), *p* < 0.001	0.44 (0.41–0.46), *p* < 0.001	9.0
	Single	0.58 (0.57–0.59), *p* < 0.001	0.60 (0.59–0.61), *p* < 0.001	7.4
AGO, SOC, and OCD	Dual	0.29 (0.21–0.39), *p* < 0.001	0.38 (0.27–0.52), *p* < 0.001	20.8
	Single	0.49 (0.48–0.51), *p* < 0.001	0.54 (0.53–0.56), *p* < 0.001	14.3
SCZ and BIP	Dual	0.40 (0.34–0.47), *p* < 0.001	0.43 (0.37–0.51), *p* < 0.001	8.3
	Single	0.58 (0.57–0.60), *p* < 0.001	0.60 (0.58–0.61), *p* < 0.001	4.6
Starting University^f^				
Any Disorder	Dual	0.40 (0.39–0.42), *p* < 0.001	0.51 (0.49–0.53), *p* < 0.001	33.0
	Single	0.65 (0.64–0.66), *p* < 0.001	0.71 (0.70–0.72), *p* < 0.001	29.7
Neuropsychiatric disorders	Dual	0.34 (0.24–0.49), *p* < 0.001	0.38 (0.27–0.55), *p* < 0.001	22.1
	Single	0.58 (0.56–0.61), *p* < 0.001	0.60 (0.58–0.63), *p* < 0.001	19.7
SUD	Dual	0.28 (0.26–0.29), *p* < 0.001	0.40 (0.38–0.42), *p* < 0.001	34.0
	Single	0.51 (0.50–0.52), *p* < 0.001	0.60 (0.59–0.61), *p* < 0.001	32.4
GAD and MDD	Dual	0.62 (0.58–0.66), *p* < 0.001	0.67 (0.63–0.71), *p* < 0.001	27.4
	Single	0.76 (0.75–0.77), *p* < 0.001	0.78 (0.77–0.79), *p* < 0.001	23.4
AGO, SOC, and OCD	*Dual*	*0.58* (*0.37–0.91*), *p* < *0.05*	*0.99* (*0.63–1.55*), *p* *=* *0.960*	*87*.*7*
	Single	0.66 (0.64–0.69), *p* < 0.001	0.77 (0.74–0.80), *p* < 0.001	43.6
SCZ and BIP	*Dual*	*0.73* (*0.61–0.88*), *p* < *0.01*	*0.84* (*0.61–1.02*), *p* *=* *0.085*	*43*.*6*
	Single	0.85 (0.83–0.87), *p* < 0.001	0.86 (0.84–0.88), *p* < 0.001	20.2

*Abbreviation:* AGO, agoraphobia; BIP, bipolar disorder; GAD, generalized anxiety disorder; MDD, major depressive disorder; OCD, obsessive-compulsive disorder; SCZ, schizophrenia; SOC, social phobia; SUD, substance use disorders; USS, upper secondary school.

a ‘Uncleaned population’ imposes no restriction on parental diagnoses in the comparison group and represents, in each relevant analysis, the rest of study population. This approach reflects the high rates of psychiatric comorbidity and ensures obtaining real-world, conservative measures of associations.

b Adjusted for offspring year of birth (5-year categories starting from 1973), offspring sex, and maternal and paternal year of birth in decades (<1940, 1950s, 1960s, 1970s, ⩾1970).

c Individuals who graduated from compulsory school in 1998–2013 (*n* = 1 416 867). The graduation years were chosen due to the different eligibility criteria used prior to 1998.

d Neuropsychiatric disorder group includes attention-deficit hyperactivity disorder, ASD, and TD/CTD.

e Individuals born in 1973–1994 (i.e. aged ⩾19 by the end of follow-up) and who was alive and living in Sweden at age 19 years (*n* = 1 985 519).

f Individuals born in 1973–1992 (i.e. aged ⩾21 by the end of follow-up) and who was alive and living in Sweden at age 21 years (*n* = 1 781 145).

Note: Italicized rows indicate main effect moved from significant to non-significant on adjustment for parental education level.

### Sensitivity analyses

Main results were not altered in analyses which (1) employed a ‘cleaned population’ as the comparison group (online Supplementary Fig. S5, Tables S7–S9), (2) applied more restrictive definitions for schizophrenia and bipolar disorder (online Supplementary Fig. S6, Tables S10–S12), or (3) restricted the cohort to pairs resident in Sweden 1997 or later (online Supplementary Fig. S7, Table S13–S15).

## Discussion

Using objective measures of educational performance, this national cohort study showed a consistent, negative association between parental mental illness and educational attainment in the subsequent generation. The primary finding is one of pervasive academic underachievement among the offspring of these dually affected pairs, with variation in the magnitude of these affects by both parental disorder and education level. Crucially, these effects were shown to persist when controlling for both offspring mental health and parental education level.

Particularly striking in our study was the early age at which main effects (between parental mental health and offspring achievement) were found to emerge. The odds of completing compulsory education were reduced by half in the offspring of single-affected pairs, with a 70% reduction among families with two affected parents; in other words, while 92% of children from unaffected families completed compulsory education, only 85% of offspring from single-affected families, and 75% of children from dual-affected families, did the same. Furthermore, a higher proportion of those eligible to continue their education – from these affected families – failed to do so, a pattern that persisted from upper-secondary school through to university. The finding that these outcomes, already striking in the offspring of single-affected pairs, were doubled in the offspring of dual-affected pairings is critical, particularly in the context of recent epidemiological work (Nordsletten et al., 2016) highlighting the frequency with which affected individuals pair together. Crucially, these differences were retained across parental conditions.

While stable in showing a difference between dual and single-affected families, our results also suggested variation in the magnitude of main effects both by parental diagnosis and level of education. At the earliest levels of schooling, our models consistently indicated lower odds of success in the offspring of pairs dual-affected with SUD and anxiety-related disorders (e.g. generalized anxiety, agoraphobia, social phobia, OCD; aOR range: 0.12–0.21), with the lowest adjusted odds of completing compulsory school found among pairs in which the father was diagnosed with agoraphobia and the mother social phobia (aOR 0.12). These differences were reflected at the level of disorder-group, where adjusted models indicated the lowest odds of success in pairs dual-affected with SUD (aOR 0.24) and agoraphobia, social phobia, and OCD (aOR 0.24). In contrast, for completion of USS, offspring of pairs with neuropsychiatric disorders showed the largest reductions in attainment (aOR 0.19); among the offspring of fathers with these conditions, outcomes were particularly, and consistently, reduced regardless of mother's particular diagnosis (e.g. for ADHD, aOR range across mother's diagnoses: 0.17–0.25 *v.* aOR range across father's diagnoses: 0.14–0.34). Across education level, the most stable impacts emerged for the offspring of dual-affected pairs in which at least one parent had SUD; among pairs in which both parents had such a diagnosis, the odds of their offspring reaching discrete education milestones was substantially reduced from the earliest (aOR 0.27), to the latest (initiating and finishing University; aOR 0.33 and 0.32, respectively). These figures varied only slightly when pairs were mixed (e.g. SUD in one partner, the other diagnosed with any of the remaining ten conditions) and did not fluctuate meaningfully depending on which parent was affected.

Of note, this finding of diminished attainment, independent of the diagnostic structure of the parental pairs (e.g. which parent has a given diagnosis), was sustained across the majority of diagnostic combinations we examined. While select differences emerged – at the level of USS, for instance, lower odds of success were seen among offspring from families in which fathers had an ASD diagnosis and mothers ADHD (OR 0.14), *v.* the opposite structure (OR 0.24) – our global finding is one of substantial and, for dual-affected pairs, additive, links between parental psychopathology and offspring educational attainment, regardless of parent sex. In the context of the substantial literature emphasizing the role of maternal mental health in offspring outcomes, such findings signal the need for broadened recognition and scientific consideration of the independent and shared influence the mental health of *both* parents holds for offspring opportunity and achievement. That the associations under study here emerged at the earliest levels of school, and remained regardless of accounting for parents own education or children's own mental health, signal the particular importance of extending work inclusive of fathers beyond adolescence and adulthood, and into early childhood development. We echo calls, from others in this arena (Ramchandani et al., 2005; Ramchandani & Psychogiou, 2009), for furthered investment in such study.

There is a direct, positive relationship between years of education and occupational, behavioral and health outcomes – extending not only to quality, but also duration of life - with departure from school at an early age associated with marked disadvantage in these crucial domains (Freudenberg & Ruglis, 2007). In Sweden, for instance, where our data is derived, a longitudinal study focused on early-leavers of compulsory school showed these individuals to be at high risk for persistent unemployment, with correspondent increases in poor health behaviors (e.g. smoking), mental health difficulties (including higher depressive symptoms, restlessness, lack of concentration, and anxiety), as well as negative somatic symptoms (particularly among men) (Hammarström & Janlert, 2002). Among early-leavers finding work, low pay, job instability, and heightened barriers to job re-entry have, further, been found as unique challenges to those at the lowest education levels (Wolbers, 2000). The cumulative burden of these disadvantages has been shown to dramatically increase years lived with activity limitations, with reductions in life expectancy exceeding 7 years in males [among females, mortality was lower (4 years), though activity restriction was higher]. Such findings have been replicated in works across the globe. Our results indicate that the children of partners with mental illness face a dramatically increased likelihood for such outcomes, with the children of dual-affected parents representing a particularly high-risk group. Future work will be needed to quantify any such effects (e.g. on labor-market and life-quality outcomes) in this uniquely high-risk group.

Regarding mechanisms of these effects, previous work has demonstrated uniquely high risks for psychiatric difficulties in the offspring of parents who are both affected with major psychiatric illness. Children of parents dual-affected with schizophrenia or bipolar disorder have, for example, been shown in both Danish and Swedish national samples (Gottesman et al., 2010; Nordsletten et al., 2020) to have substantially increased lifetime risks for these conditions (18–27% for schizophrenia; 19–25% for bipolar disorder), rising to a minimum of 43% (up to a maximum 67%) for *any* major psychiatric disorder. Similar to the patterns seen here for education, these risks appear largely retained regardless of which parent is affected with a given disorder and, indeed, whether parents differ in their respective diagnosis (Nordsletten et al., 2020). Our present findings, which controlled for offspring's own diagnostic status, indicate that the relationship between parental mental health and offspring educational outcomes extends meaningfully beyond the clinic, impacting core, functional domains regardless of the presence of a psychiatric condition.

The factors directly influencing these outcomes are likely to be complex. Just as reduced education is associated, indelibly, with reductions in opportunity and life quality, so too are mental disorders linked with unstable employment, poverty, marital instability, and other environmental risks and stressors linked to poor child development (e.g. chaotic home, neighborhood deprivation) (Sariaslan et al., 2016) – the burdens of which may be doubled in families with two affected parents, impacting offspring outcomes in the manner observed here. Genetics, including pleiotropic genes which contribute to both psychiatric risk and cognitive ability (Hagenaars et al., 2016; Ohi et al., 2018) – or which mediate the relationship between cognitive ability and school performance (Rimfeld, Kovas, Dale, & Plomin, 2016) – may, likewise, be clustered within dual-affected pairs with consequent impacts on the variance of their offspring with respect to risk for poor academic outcomes. Our own analyses suggest the dynamics of such inter-relationships may vary, not only by parental disorder (e.g. greater mediation effects among the offspring of parents with select disorders/disorder groups, such as anxiety disorders, neuropsychiatric conditions, or SUD) but also time (e.g. stronger mediation was observed for compulsory schooling and University *v.* secondary-school achievement). Further work will be needed to disentangle the role – potentially, additive – of these, and other, related, factors in the etiology of the outcomes seen in our samples.

### Strengths and limitations

In addition to being based on a uniquely large nationwide cohort, our findings were strengthened by the use of validated diagnoses of psychiatric disorders and availability of prospectively and uniformly collected clinical and sociodemographic data from independent nationwide Swedish registers, that altogether minimized the risk of selection, recall, and report biases and decreased diagnostic misclassification.

Several limitations should also be acknowledged. First, registered psychiatric diagnoses are likely to underrepresent less severe cases, since the NPR does not include data from primary care. Information from outpatient specialized care, better suited to detecting these cases, has been available since 2001 and was used to augment our diagnostic detection procedures. Second, USS and university education are voluntary forms of schooling in Sweden and, therefore, an individual's attendance and achievements may be driven by reasons including, but not limited to, exposure to parental psychopathology. However, with the choice to engage in schooling equal between groups, our findings strongly support a relationship between parental diagnostic status and these outcomes. Third, despite the completeness of the MGR (Ekbom, 2011; Statistics Sweden, 2017), data on biological paternity are self-reported by the mother. According to an international review, the likelihood of paternal discrepancies remains low (median 3.7%) (Bellis, Hughes, Hughes, & Ashton, 2005). On a related point, the MGR's focus on biological (e.g. sex-based) parentage (e.g. mothers and fathers) has limited our ability to explore dynamics by family social structure, including variation in associations by gender identity of both parents. Fourth, our exclusion criteria – established to mirror extant register-based studies on education or related outcomes (Niederkrotenthaler et al., 2014; Perez-Vigil et al., 2018a, 2018b; Vilaplana-Perez et al., 2020a, 2020b, 2021) – resulted in loss of a proportion of the potential study population (ranging from 0.26% for offspring excluded due to adoption status, to 6.25% for offspring excluded due to having both parents with foreign-born background or with missing information on origin). Though we cannot fully rule out the risk of selection bias due to these omissions, given the scale of the data we consider the risk low and, crucially, the exclusions necessary for consistency with prior work and to avert to confounding relationships between select factors (e.g. foreign birth) and our core outcomes. Finally, despite a large study size, our analyses were underpowered for analyses parsing causal factors (e.g. the role of environment *v.* genetics, via analysis of adoptees) and, further, for capturing associations using select disorder combinations, particularly at the post-compulsory education level. These latter values have been omitted and marked for clarity.

## Conclusions

The offspring of parents with major psychiatric disorders, particularly those for whom both parents are affected, represent a group at ultra-high-risk for early school departure. These findings make a strong case for investment in preventive strategies, aimed at parenting and family support to mediate the relationship between parental mental health and children's academic attainment.

## Supporting information

Sidorchuk et al. supplementary material Sidorchuk et al. supplementary material

## Supplementary material

The supplementary material for this article can be found at https://doi.org/10.1017/S0033291722003506
