# Supplementary material for: One versus two biological parents with mental disorders: Relationship to educational attainment in the next generation
Source: Psychol Med. Author manuscript; Available in PMC 2024 May 1. (PMC10719631; doi:10.1017/S0033291722003506)
Supplement: 2 [file NIHMS1915975-supplement-2.pdf]

Supplementary Materials for

One versus two biological parents with mental disorders: Relationship to educational attainment in the next generation

Anna Sidorchuk, Gustaf Brander, Ana Pérez-Vigil, James J. Crowley, Henrik Larsson, Paul Lichtenstein, David Mataix-Cols, Ashley E. Nordsletten*

Correspondence to: nordslae@med.umich.edu

This PDF file includes:

Supplementary Text (Notes 1, 2, & 3)
Figs. S1A to S7
Tables S1A to S15
References

Supplementary Text

Note 1. Overview of the Swedish National Registers

For the present investigation, a population-based birth cohort was assembled by linking data across the following nationwide Swedish registers via the unique personal identification number assigned to all Swedish residents:

The Multi-Generation Register, which contains information on biological and adopted parents of all individuals born (since 1932) or registered (since 1961) in Sweden *(Ekbom, 2011)*.

The National Patient Register, which captures diagnostic information from inpatient care (since 1969 and 1973 for somatic and psychiatric disorders, respectively, with complete nationwide coverage from 1987) and specialist outpatient care (since 2001); discharge diagnoses are recorded and coded utilizing the Swedish version of the International Classification of Diseases, Eighth Revision (ICD-8) (1969-1986), ICD-9 (1987-1996), and ICD-10 (1997-onwards) *(Ludvigsson et al., 2011)*.

The Cause of Death Register covers all dates and causes of deaths of Swedish residents since 1952, and was used to determine follow-up time in the current study *(Brooke et al., 2017)*.

The Prescribed Drug Register records all prescribed medications dispensed across pharmacies in Sweden since July 2005, using Anatomical Therapeutic Chemical (ATC) Classification System codes, with a selection of drug codes used to define clinical features (e.g., ADHD) in our study sample *(Wettermark et al., 2007)*.

The Total Population Register holds nationwide demographic and migration data since 1968, and was used to identify our base samples *(Ludvigsson et al., 2016)*.

Educational outcomes were defined, in part, via *the Longitudinal Integration Database for Health Insurance and Labour Market Studies (LISA)*, which records educational attainments and other socioeconomic data for all residents (age 16+) since 1990 *(Ludvigsson, Svedberg, Olen, Bruze, & Neovius, 2019)*. *The National School Register* was also utilized, with this register holding individual-level data on school performance for all students graduating from municipal and independent schools in Sweden since 1988 *(The Swedish National Agency for Education, 2018a)*.

Citations for all registers are provided in the main text and at the end of the Supplementary Materials, and offer detail on formation and utilization of each in register-based research. Conventions of usage have been followed in execution of the current investigation.

Note 2. Overview of the Swedish education system

Education in Sweden is governed by the state on the principle of equal access for all, and generally publicly financed *(The Swedish National Agency for Education, 2000)*. Overall, the system includes the following sectors: 1) Pre-school (attended at ages 1 to 5); 2) Pre-school class (age 6); 3) Primary and lower secondary education (ages 7 to 16); 4) Upper secondary education (ages 16 to 18-19); 5) Special education (ages vary); 6) Further education for adults (ages vary); 7) Higher education (ages vary) *(Deen, 2007)*. The current study focuses on

measuring outcomes which are defined by the sectors ##3, 4, and 7; see description in details below.

Compulsory schooling and eligibility to access upper secondary school

The primary and lower-secondary education is 9 years in length and compulsory by law *(The Swedish National Agency for Education, 2000; Deen, 2007)*. During the education process, each student takes 16 compulsory subjects: Swedish language, mathematics, physical education and health, English language, handicrafts (textile, wood, and metalwork), music, visual arts, technology, physics, chemistry, biology, history, social studies, religious knowledge, geography, and home economics; and from the year six, the students can take a non-compulsory subject on second foreign language *(The Swedish National Agency for Education, 2018b)*.

Starting from the year six, students are awarded grades, in accordance with an achievement-related model *(The Swedish National Agency for Education, 2018b, 2022)*. Upon the graduation in the ninth year, students are awarded the final grades for each subject which are registered in school-leaving certificates and recorded in the National School Register (held by Statistics Sweden; https://www.scb.se/) *(The Swedish National Agency for Education, 2018a)*. Since 1988, the Register includes nationwide individual-level information on the final grades for each subject from all public and private compulsory schools in Sweden. The final grades qualify students to apply for upper secondary school and determine their eligibility to access either vocational program or higher education preparatory program (also called 'academic program'). Eligibility to access vocational program requires attaining a passing grade in 3 'core subjects' (i.e., Swedish language, mathematics, and English language) and, since 2011, also requires passing additional 5 subjects (totally, 8 subjects). Eligibility to access academic program requires passing 3 core subjects and 9 additional subjects (totally, 12 subjects).

If students are ineligible for accessing a certain program at upper secondary school, they can attend one of the introductory programs which helps to acquire knowledge and skills to enter the labor market or to gain competence in subject(s) in which the student lacks a passing grade *(Upper secondary education, 2020)*. Once the competence in required subjects is achieved and eligibility criteria for accessing a certain program at upper secondary school are met, the student can be accepted on a program at upper secondary school given places are available.

In the current study, data from the National School Register was used to define the outcome status of each study participant on their eligibility to access upper secondary school. The eligibility was assessed in individuals who graduated from compulsory school in 1998-2013. The graduation years were chosen due to differing eligibility criteria used prior to 1998 (since the new compulsory school curriculum had been implemented in Sweden during 1997/1998 school year). The outcome called 'eligibility to access upper secondary school' was defined based on individual-level data on passing grades received upon graduation in the subjects, which are required for eligibility to access a vocational program (i.e., the lowest requirement to access upper secondary school).

Upper secondary school

This is a voluntary form of schooling that the students can attend after completing the compulsory education. Upper secondary school is 3 years in length and tuition-free. Prior to 2011, upper secondary school education included 17 national programs, of which 14 programs were vocationally oriented with a primary aim of preparing the students for working life, and the reminding 3 programs focused on preparing students for university studies *(Deen, 2007)*. Since 2011, upper secondary school offers 18 national educational programs, of which 12 programs refer to vocational program (e.g., building and construction program; electricity and

energy program; vehicle and transport program; business and administration program, etc.) and the other 6 programs refer to academic program (e.g., business management and economics; arts; humanities; natural science; social science; technology) *(The Swedish National Agency for Education, 2013)*.

Each program consists of several courses which provide a certain number of points. All programs include 8 core subjects, such as Swedish/Swedish as a second language, English language, mathematics, civics, religion, science studies, physical education and health, and artistic activities (and history since 2011), as well as program-specific subjects. Students are graded at the end of each course, in accordance with the national knowledge requirements laid down for each course. In addition, students are graded after completion of a diploma project. A final grade, which is awarded upon completion of the whole program, is registered in a school-leaving certificate. The certificate denotes the status of having completed upper secondary education, which is recorded in the Educational Register and the Longitudinal Integration Database for Health Insurance and Labour Market Studies (LISA; Swedish acronym for Longitudinell Integrationsdatabas för Sjukförsäkrings- och Arbetsmarknadsstudier; held by Statistics Sweden; https://www.scb.se/) *(Ludvigsson, Svedberg, Olen, Bruze, & Neovius, 2019)*. Since 1990, LISA annually collects educational data on each individual aged 16 years and above who is alive and registered in Sweden as of December 31st each year along with information on completeness/incompleteness and length of education (in years) at each educational level *(Ludvigsson, Svedberg, Olen, Bruze, & Neovius, 2019)*. The prospective and uniform collection of data on education in LISA allows to follow each individual throughout his/her lifetime educational development.

In the current study, LISA was used as a source of data on post-compulsory education. The outcome called 'finishing upper secondary school' was defined by individual-level data from LISA on completeness/incompleteness of either vocational or academic program and the length of upper secondary education. For this outcome, all analyses were based on a sub-cohort of individuals born in 1973-1994 (i.e., who were aged 19 years and above by the end of follow-up on December 31st, 2013) and who did not die or emigrated from Sweden before age 19.

Higher education

Higher education is a voluntary form of post-secondary education, which is offered by universities and university colleges (also called tertiary education). Higher education in Sweden is provided at the undergraduate level, including Bachelor's degree and Master's degree education as well as at the postgraduate level, i.e. Doctoral degree education *(Deen, 2007)*. Postgraduate level education is beyond the focus of the current study. For citizens of Sweden (or citizens of European Union or European Economic Area country, or Switzerland), a higher education in Sweden is tuition-free *(Willemse &De Beer, 2012)*. Through the Swedish Board of Student Finance (https://www.csn.se/), students with Swedish citizenship are eligible for the state financial support for their university studies in a form of student loans or study grants that minimizes the influence of financial aspect on the decision-making process of attaining higher education.

Each educational level within higher education is built on achieving a prior level. Thus, to study at bachelor level, students must have successfully completed upper secondary education or its equivalent. To enter a program at the master's level, students have a Bachelor's degree from Sweden or a foreign country, or have a corresponding qualification. Higher education institutions can vary in educational process, but it is overall organized through offering courses and programs; with specific entry requirements for each course and program. The duration and extent of programs and courses is measured by the higher education credits and one full-time academic year is equal to 60 credits. Bachelor's degree requires of at least 180 higher education credits (3

years in length) and Master's degree requires additional 60 or 120 credits (additional 1-year education results in a Magister degree [in Swedish: Magisterexamen] and 2-year education leads to a Master degree [in Swedish: Masterexamen]; both degrees are translated as a 'Degree of Master' in English). At each level, the students can earn a general qualification, an artistic qualification, or a professional qualification. The latter qualification leads to a specific profession and implies that students must have completed an occupation-specific program in order to be licensed to work in such profession (for example: a Degree of Bachelor of Science in Social Work, a Degree of Master of Science in Medicine, etc.). As indicated above, annually updated information on educational status of each Swedish resident (aged 16+) is registered in LISA *(Ludvigsson, Svedberg, Olen, Bruze, & Neovius, 2019)*. For education at post-secondary level, LISA records the current length of studies, the number of obtained higher education credits, presence or absence of a degree, type of the program taught, and whether education is undertaken at universities/university colleges or not.

In the current study, the outcome called 'starting university' was defined by information from LISA on having undertaken post-secondary education for up to 3 years in length without being awarded a degree. The outcome called 'finishing university' was denoted by LISA records of having studied at universities/university colleges for 3 years or more, i.e., having fulfilled the requirements for a degree. For the former outcome, the analyses were based on a sub-cohort of individuals born in 1973-1992 (i.e., who were aged 21 years and above by the end of follow-up on December 31st, 2013) and who did not die or emigrated from Sweden before age 21. For the latter outcome, the sub-cohort of individuals born in 1973-1988 was used, i.e., the analyses were conducted among those who were aged 25 years and above by the end of follow-up and who did not die or emigrated from Sweden before age 25.

Summary of Education Outcome Definitions

For each educational outcome in our analyses, separate sub-cohorts of index offspring were created, capturing individuals with 1) the minimum person-time necessary to achieve the corresponding outcome (Fig. S4) and 2) similar definitions of the target outcomes. Thus, eligibility to access USS was assessed in offspring who graduated from compulsory school in 1998-2013 (n=1,416,867), with the graduation range defined due to the equivalence of eligibility criteria used in this period. For finishing USS, the sub-cohort included individuals born in 1973-1994 (i.e., aged 19+ by the end of follow-up) and who were alive and living in Sweden at age 19 (n=1,985,519). For starting university education, the sub-cohort consisted of those born 1973-1992, who were alive/in Sweden at age 21 (n=1,781,145). Finally, the sub-cohort for finishing university included those born 1973-1988, who were alive/in Sweden at age 25 years (n=1,344,192). All age cut-offs were drawn from categories defined in reporting on the Swedish educational system.

Note 3. Description of the 'uncleaned population' used for comparison

For the main analyses, the comparison pairs were selected from the general population using an ***'uncleaned' technique*** (Gottesman et al., 2010). This applies to all analyses for exposure type 1 (see below Supplemental Fig S2A for details), exposure type 2 (see Supplemental Fig 2B), and exposure type 3 (see Supplemental Fig S3). This approach imposes no restriction on parental diagnoses and represents, for each of the exposure types, the remainder of the full study population. This method retains and reflects the high rates of comorbidity inherent to psychiatric populations and ensures real-world, conservative measures of associations. Below we present the examples for different exposure types. The parental psychiatric disorders that compose the

exposure variables include 11 disorders as: attention-deficit/hyperactivity disorder (ADHD), autism spectrum disorder (ASD), schizophrenia, bipolar disorder, major depressive disorder (MDD), generalized anxiety disorder (GAD), agoraphobia, social phobia, obsessive-compulsive disorder (OCD), substance use disorders (SUD), and Tourette's disorder and chronic tic disorders (TD/CTD) (see below Supplemental Table S1A for the ICD-codes).

The *exposure type 1* is defined as: parent(s) affected with any of 11 disorders of interest (i.e., pairs in which ≥ 1 member has a disorders of interest). These pairings included: (i) dual-affected pairs (i.e., both parents affected; pairs permitted to share or differ in their respective diagnoses), and (ii) single-affected pairs (i.e., only one parent affected) (Supplemental Fig S2A). The *uncleaned comparison population for offspring exposed to having dual-affected parents* includes the rest of the offspring from the sub-cohort (please note that for each specific educational outcome a separate sub-cohort is used as reported in Supplemental Fig 1A and 1B), i.e., offspring of pairs where either none of parents are affected by any of 11 disorders or only one parent is affected. The *uncleaned comparison population for offspring exposed to having single-affected parent* consists of offspring of parents not affected by any of 11 disorders.

The *exposure type 2* is defined as: the disorders of interest are clustered into five operational groups: 1) neuropsychiatric disorders (ADHD, ASD, and TD/CTD), 2) SUD, 3) GAD and MDD, 4) agoraphobia, social phobia, and OCD, and 5) schizophrenia and bipolar disorder, and within each disorder group, exposure variables are generated– same as in exposure type 1 - to distinguish dual-affected and single-affected pairs (e.g., 'dual-affected' with neuropsychiatric disorders would indicate both members have ADHD, ASD, or TD/CTD [in any combination], while in single-affected pair only one parent has neuropsychiatric disorders [in any combination] but not the other; same for all other disorder groups) (Supplemental Fig S2B). Using the group of neuropsychiatric disorders as an example, the *uncleaned comparison population for offspring exposed to having dual-affected parents* includes offspring of pairs where either none of parents are affected by neuropsychiatric disorders (but parents might be dual- or single-affected by any of the remaining disorder groups), or only one parent is affected by neuropsychiatric disorders. The *uncleaned comparison population for offspring exposed to having single-affected parent* includes offspring from families where none of parents are affected by neuropsychiatric disorders (but parents might be dual- or single-affected by any of the remaining disorder groups). Same principals apply for each other disorder groups.

The *exposure type 3* is defined as: parent(s) affected with a specific disorder of interest: exposure groups are also generated for each of 11 disorders of interest. For each specific disorder, we identified all (i) within-disorder dual-affected pairs (i.e., mother and father with the same specific disorder, e.g., with OCD), (ii) cross-disorder dual-affected pairs (mother and father with different specific disorders, e.g., mother with OCD, father with MDD), and (iii) single-affected pairs (only one parent has a specific disorder of interest, e.g., OCD, but the other parent id free from this particular disorder) (Supplemental Fig S3). Using pairs dual-affected with OCD as an example, the *uncleaned comparison population for offspring exposed to having within-disorder dual-affected parents* consists of offspring of pairs where either none of parents are affected by OCD (but they might be dual- or single-affected by any other disorders), or only one parent is affected by OCD. Using pairs where mother is affected with OCD and father is affected with MDD as an example, *the uncleaned comparison population for offspring exposed to having cross-disorder dual-affected parents* consists of offspring from any other families where such combination of maternal and paternal disorders was not identified (but mother and/or father might be affected by other disorders). For pairs where only one, but not the other parent is affected with a specific disorder (e.g., OCD), *uncleaned comparison population* consists of offspring where none of parents have OCD, but they might be affected (one or both) by any other disorders.

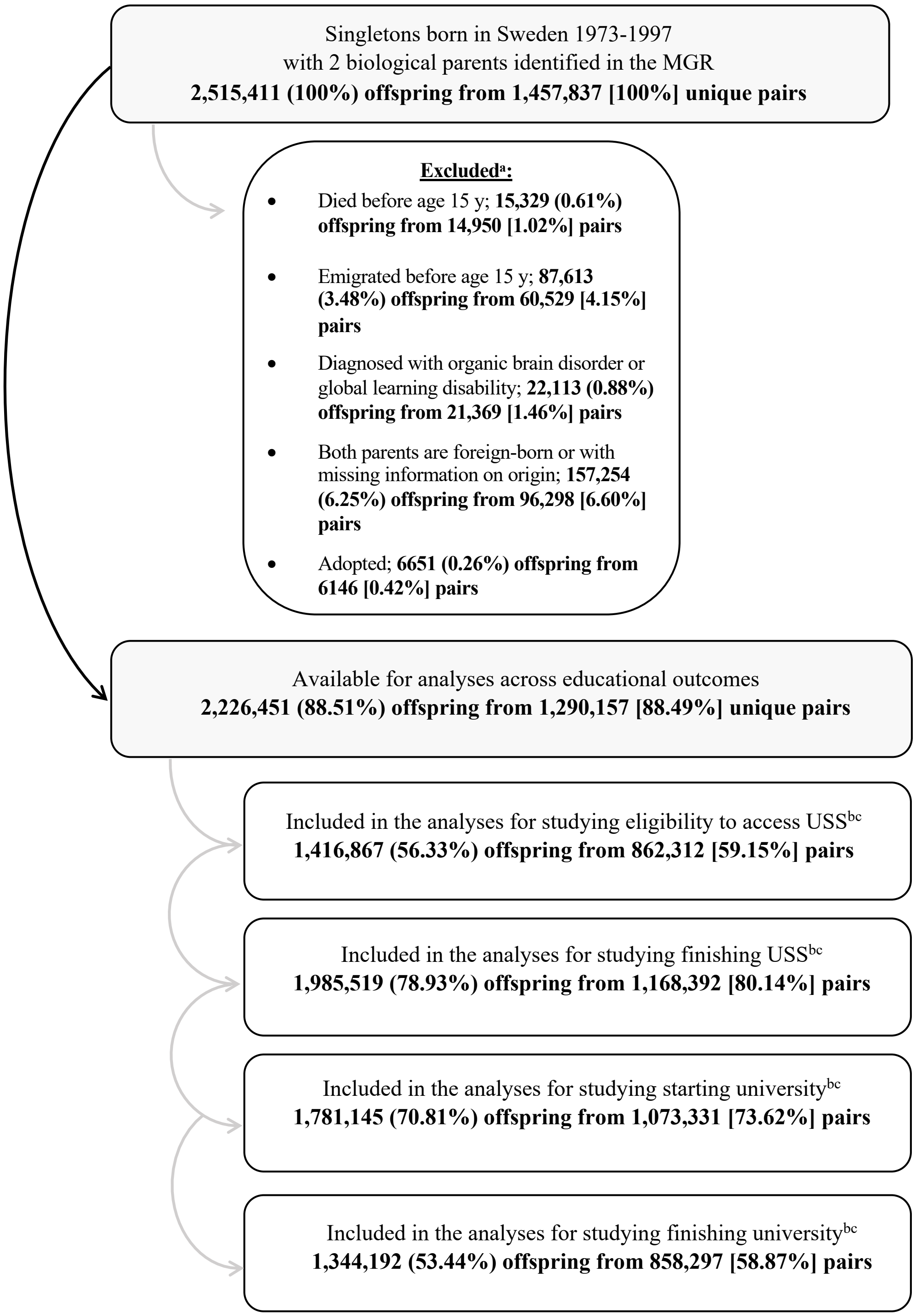

Fig. S1A. Study inclusion/exclusion flowchart

[a] The same parental pairs may appear in several exclusion categories if they have two or more offspring who fulfil different exclusion criteria
[b] The details on how the offspring (and corresponding parental pairs) were selected for the analyses of each educational outcome are reported in **Supplementary Figure S1**.
[c] The same offspring and parental pairs may be included in the analyses of several educational outcomes (see **Supplementary Figure S1** for details).

Abbreviations: MGR; Multi-Generation Register, USS; Upper secondary school

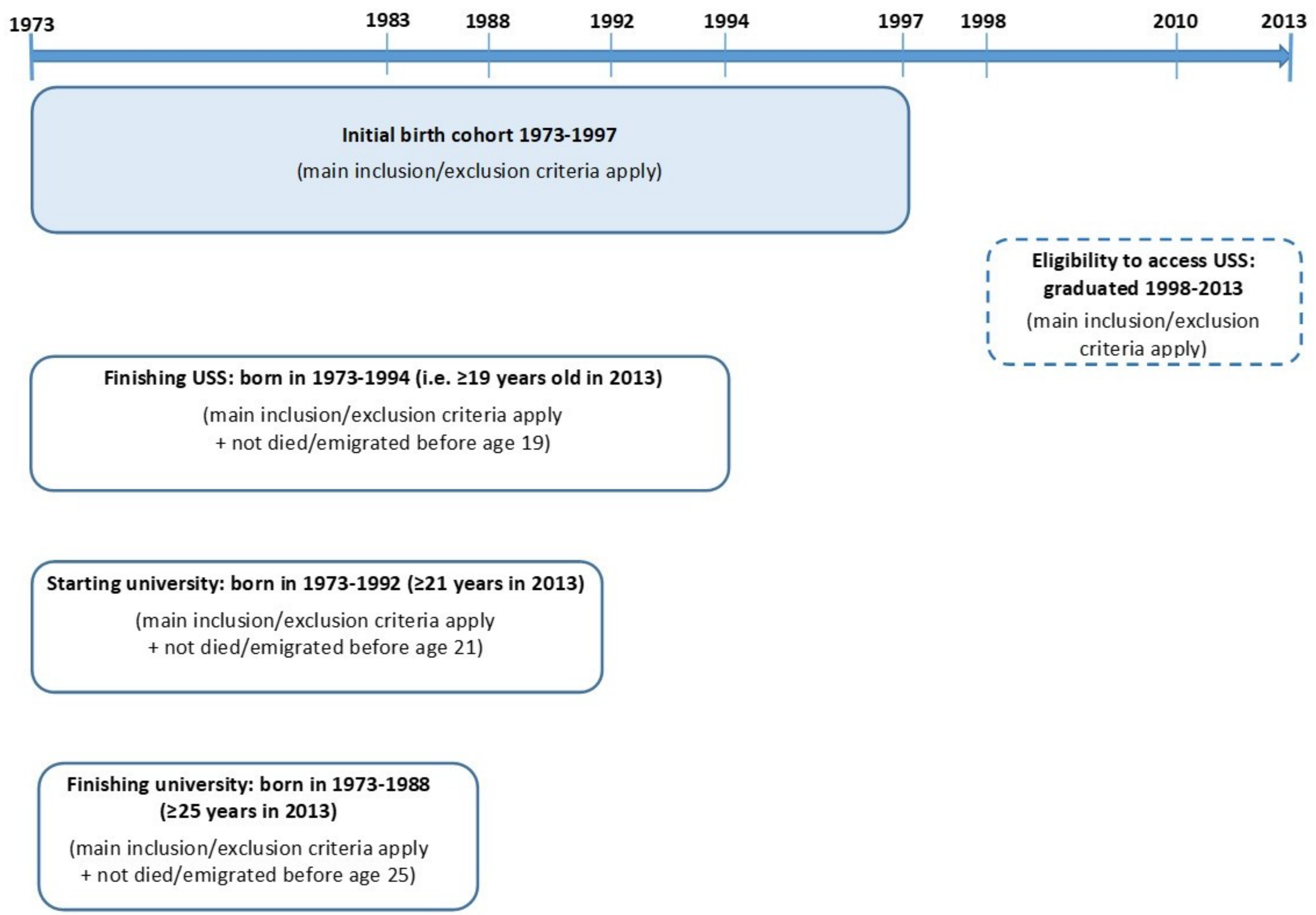

Fig. S1B. Study population and sub-cohorts (one for each educational outcome).

Note: White box with dotted margins denotes the sub-cohort for the analysis of compulsory educational outcome, the other white boxes represent the sub-cohorts for the analyses of post compulsory outcomes.

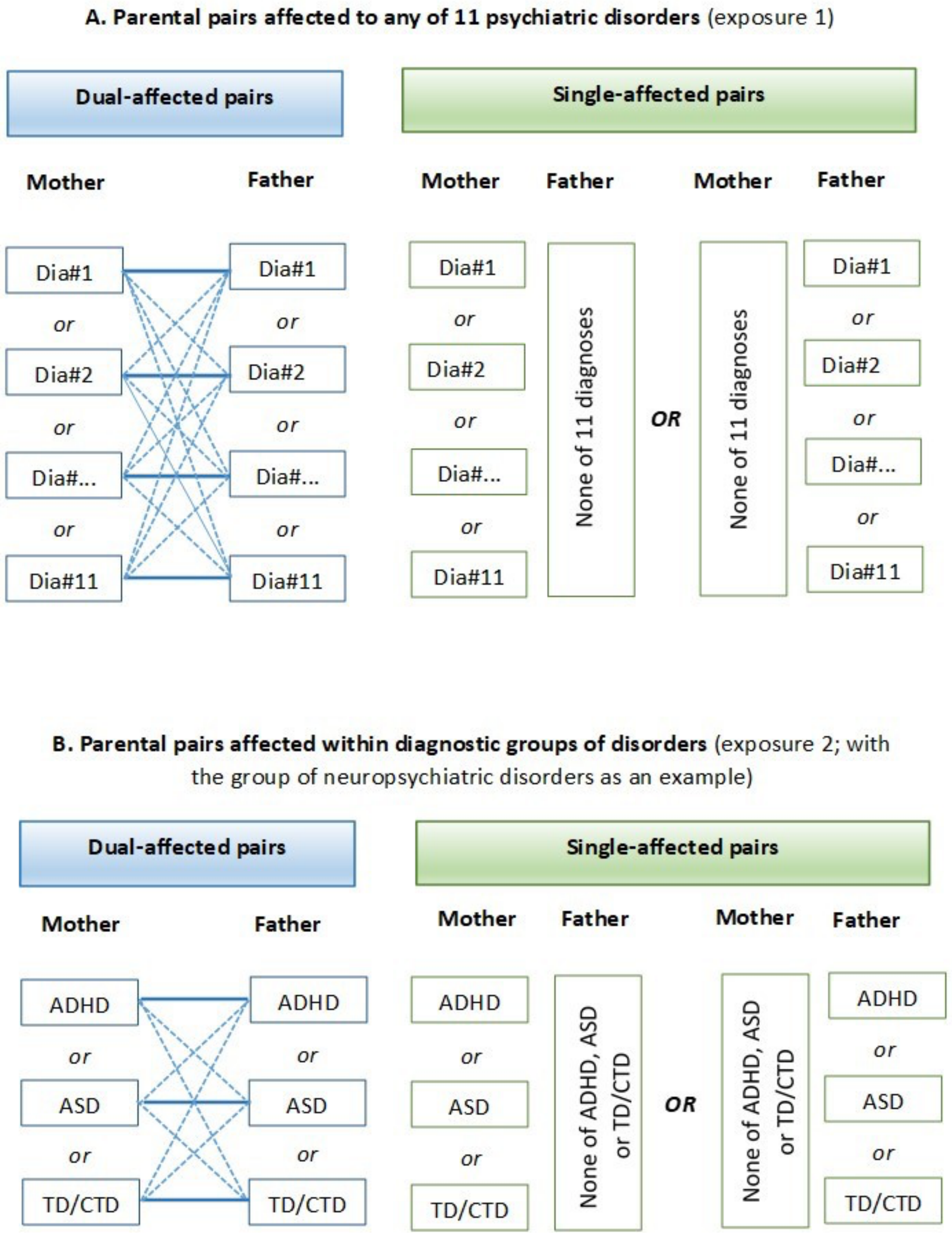

Fig. S2. Visual representation of exposure variables: (**A**) parental pairs dual-and single affected by any of 11 psychiatric disorders (exposure 1) and (**B**) parental pairs dual-and single affected within diagnostic groups of disorders (exposure 2 [with the group of neuropsychiatric disorders as an example]).

Note: In the main analysis and the supplementary analyses #2 and #3, the reference group is composed of an 'uncleaned population', while in the supplementary analysis #1 the reference group is composed by a 'cleaned population'. '*Uncleaned population*' imposes no restriction on parental diagnoses in the comparison group and represents, in each relevant analysis, the rest of study population. This approach reflects the high rates of psychiatric comorbidity and ensures obtaining real-world, conservative measures of associations. '*Cleaned population*' implies that the reference group is composed of the offspring with both parents being free from any 11 psychiatric disorders.

Abbreviations: ADHD, attention-deficit hyperactivity disorder; ASD, autism spectrum disorder; TD/CTD Tourette's disorder and chronic tic disorders.

Parental pairs affected by specific psychiatric disorder (one disorder at the time) or combination of disorders (exposure 3)

Dual-affected pairs

Paternal disorders

Maternal disorders	fADHD	fASD	fSCZ	fBIP	fMDD	fGAD	fAGO	fSOC	fOCD	fSUD	fTD/CTD
mADHD	Within	Cross	Cross	Cross	Cross	Cross	Cross	Cross	Cross	Cross	Cross
mASD	Cross	Within	Cross	Cross	Cross	Cross	Cross	Cross	Cross	Cross	Cross
mSCZ	Cross	Cross	Within	Cross	Cross	Cross	Cross	Cross	Cross	Cross	Cross
mBIP	Cross	Cross	Cross	Within	Cross	Cross	Cross	Cross	Cross	Cross	Cross
mMDD	Cross	Cross	Cross	Cross	Within	Cross	Cross	Cross	Cross	Cross	Cross
mGAD	Cross	Cross	Cross	Cross	Cross	Within	Cross	Cross	Cross	Cross	Cross
mAGO	Cross	Cross	Cross	Cross	Cross	Cross	Within	Cross	Cross	Cross	Cross
mSOC	Cross	Cross	Cross	Cross	Cross	Cross	Cross	Within	Cross	Cross	Cross
mOCD	Cross	Cross	Cross	Cross	Cross	Cross	Cross	Cross	Within	Cross	Cross
mSUD	Cross	Cross	Cross	Cross	Cross	Cross	Cross	Cross	Cross	Within	Cross
mTD/CTD	Cross	Cross	Cross	Cross	Cross	Cross	Cross	Cross	Cross	Cross	Within

Single-affected pairs

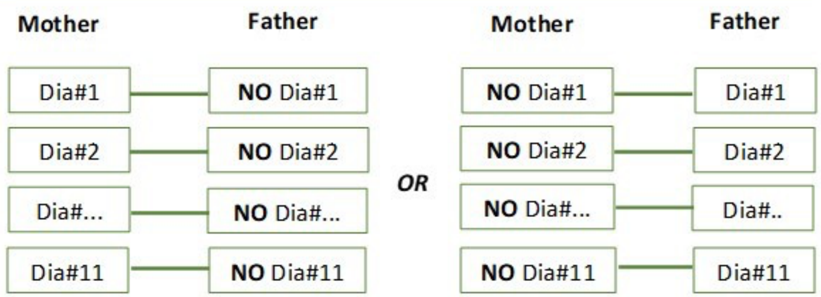

Fig. S3. Visual representation of exposure variables: parental pairs affected by specific psychiatric disorder (one disorder at the time) or combination of disorders, including within- and cross-disorder dual-affected pairs, and pairs single affected by a specific disorder (exposure 3)

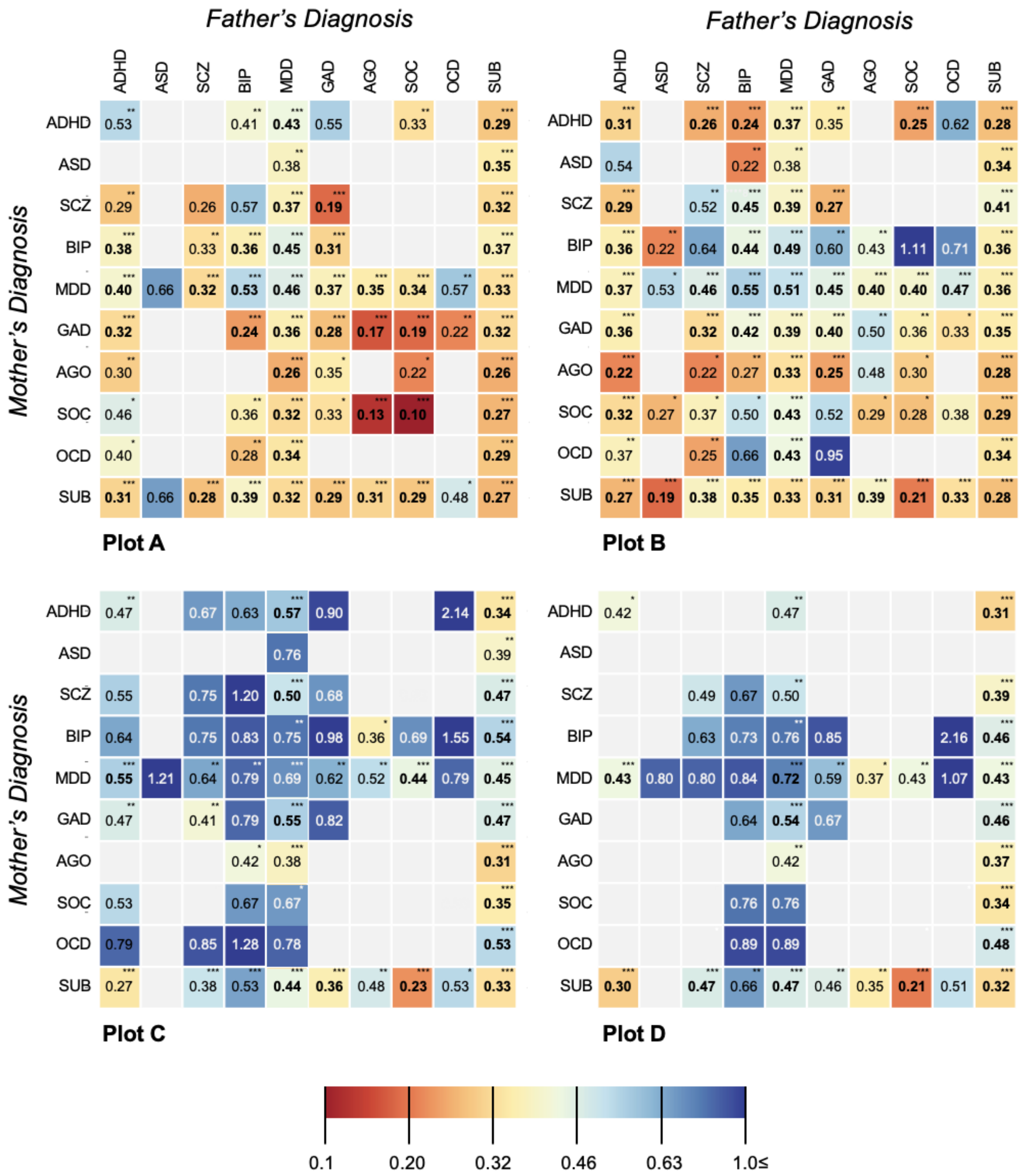

Fig. S4. Educational outcomes in offspring free from any of 11 psychiatric disorders of parents affected within-disorder and cross-disorder. Adjusted ORs and p-values (# p-value <0.05; § p-value <0.01; * p-value <0.001) for achieving (**A**) compulsory educational outcome, (**B**) finishing USS, (**C**) starting university, and (**D**) finishing university, compared with individuals with parents from the general population (*uncleaned population*[a]).

Note: All models are adjusted for offspring year of birth (5-year categories starting from 1973), offspring sex, and maternal and paternal year of birth in decades (<1940, 1950s, 1960s, 1970s, ≥1970) and clustered by family identification number with robust standard error estimation (sandwich estimator). '*Within-disorder affected parental pairs'* imply that both parents have records of the same disorder. '*Cross-disorder affected parental pairs'* imply that

parents have records of different disorders out of the eleven psychiatric disorders in question. For each educational outcome, separate sub-cohorts of index offspring were created, comprising the individuals who had the time necessary to achieve the corresponding outcome. *mXXX* denotes maternal diagnoses and *fXXX* denotes paternal diagnoses. Empty cells indicate the results of analyses with no exposed individuals or if ≤5 exposed cases or exposed non-cases were available (as a result, Tourette's disorder and chronic tic disorders are not at all reported as maternal or paternal diagnoses due to small numbers of exposed cases or non-cases). Figures in bold denote the statistically significant associations and figures in Italics denote non-significant associations.

[a] 'Uncleaned population' imposes no restriction on parental diagnoses in the comparison group and represents, in each relevant analysis, the rest of study population. This approach reflects the high rates of psychiatric comorbidity and ensures obtaining real-world, conservative measures of associations.

Abbreviation: ADHD, attention-deficit hyperactivity disorder; AGO, agoraphobia; ASD, autism spectrum disorder; BIP, bipolar disorder; GAD, generalized anxiety disorder; MDD, major depressive disorder; OCD, obsessive-compulsive disorder; OR, odds ratio; SCZ, schizophrenia; SOC, social phobia; SUD, substance use disorders.

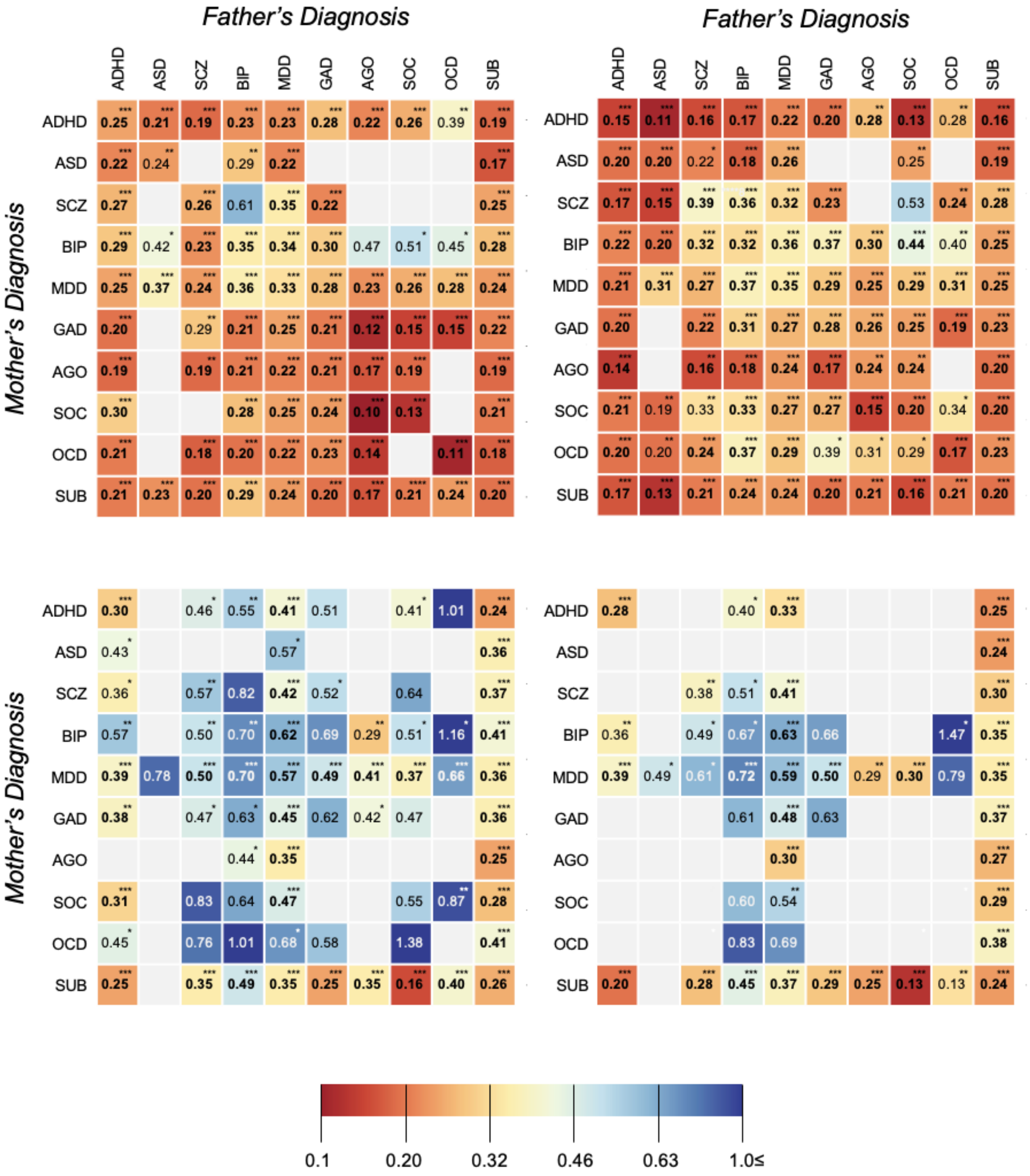

Fig. S5. <u>Sensitivity analysis #1 (comparison with cleaned population)</u>: educational outcomes in offspring of parents affected within-disorder and cross-disorder. Adjusted ORs and p-values (* p-value <0.05; ** p-value <0.01; *** p-value <0.001) for achieving (**A**) compulsory educational outcome, (**B**) finishing USS, (**C**) starting university, and (**D**) finishing university, compared with individuals with parents from the general population (*cleaned population*[a]).

Note: All models are adjusted for offspring year of birth (5-year categories starting from 1973), offspring sex, and maternal and paternal year of birth in decades (<1940, 1950s, 1960s, 1970s, ≥1970) and clustered by family identification number with robust standard error estimation (sandwich estimator). *mXXX* denotes maternal diagnoses and *fXXX* denotes paternal diagnoses. '*Within-disorder affected parental pairs'* imply that both parents have records of the same disorder. '*Cross-disorder affected parental pairs'* imply that parents have records of different disorders out of 11 psychiatric disorders in question. For each educational outcome, separate sub-cohorts of index offspring were created, comprising the individuals who had the time necessary to achieve the corresponding outcome. Empty

cells indicate the results of analyses with no exposed individuals or if ≤5 exposed cases or exposed non-cases were available (as a result, Tourette's disorder and chronic tic disorders are not at all reported as maternal or paternal diagnoses due to small numbers of exposed cases or non-cases). Figures in bold denote the statistically significant associations and figures in Italics denote non-significant associations. A 'Cleaned population' implies that the reference group is composed of the offspring with both parents being free from any 11 psychiatric disorders.

Abbreviation: ADHD, attention-deficit hyperactivity disorder; AGO, agoraphobia; ASD, autism spectrum disorder; BIP, bipolar disorder; GAD, generalized anxiety disorder; MDD, major depressive disorder; OCD, obsessive-compulsive disorder; OR, odds ratio; SCZ, schizophrenia; SOC, social phobia; SUD, substance use disorders.

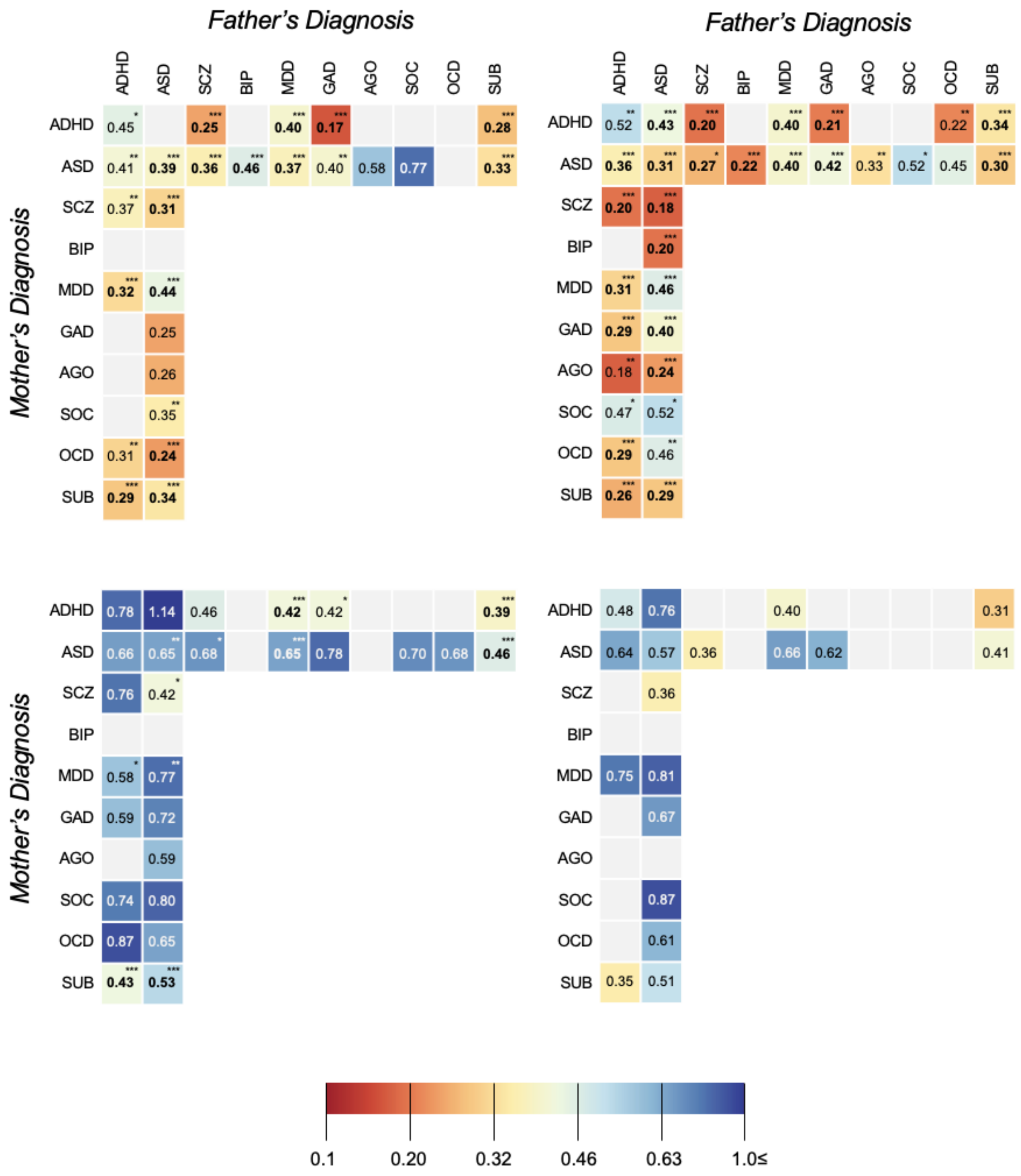

Fig. S6. <u>Sensitivity analysis #2:</u> educational outcomes in offspring with parents with repeated schizophrenia and bipolar disorders affected within-disorder and cross-disorder. Adjusted ORs and p-values ([#] p-value <0.05; [§] p-value <0.01; [*] p-value <0.001) for achieving (**A**) compulsory educational outcome, (**B**) finishing USS, (**C**) starting university, and (**D**) finishing university, compared with individuals with parents from the general population (*uncleaned population*[a]).

Note: All models are adjusted for offspring year of birth (5-year categories starting from 1973), offspring sex, and maternal and paternal year of birth in decades (<1940, 1950s, 1960s, 1970s, ≥1970) and clustered by family identification number with robust standard error estimation (sandwich estimator). *mXXX* denotes maternal diagnoses and *fXXX* denotes paternal diagnoses. '*Within-disorder affected parental pairs'* imply that both parents have records of the same disorder. '*Cross-disorder affected parental pairs'* imply that parents have records of different disorders out of 11 psychiatric disorders in question. For each educational outcome, separate sub-cohorts of index offspring were created, comprising the individuals who had the time necessary to achieve the corresponding outcome. Empty

cells indicate the results of analyses with no exposed individuals or if ≤5 exposed cases or exposed non-cases were available (as a result, Tourette's disorder and chronic tic disorders are not at all reported as maternal or paternal diagnoses due to small numbers of exposed cases or non-cases). Figures in bold denote the statistically significant associations and figures in Italics denote non-significant associations.

a 'Uncleaned population' imposes no restriction on parental diagnoses in the comparison group and represents, in each relevant analysis, the rest of study population. This approach reflects the high rates of psychiatric comorbidity and ensures obtaining real-world, conservative measures of associations.

Abbreviation: ADHD, attention-deficit hyperactivity disorder; AGO, agoraphobia; ASD, autism spectrum disorder; BIP_r, repeated diagnoses of bipolar disorder; GAD, generalized anxiety disorder; MDD, major depressive disorder; OCD, obsessive-compulsive disorder; OR, odds ratio; SCZ_r, repeated diagnoses of schizophrenia; SOC, social phobia; SUD, substance use disorder

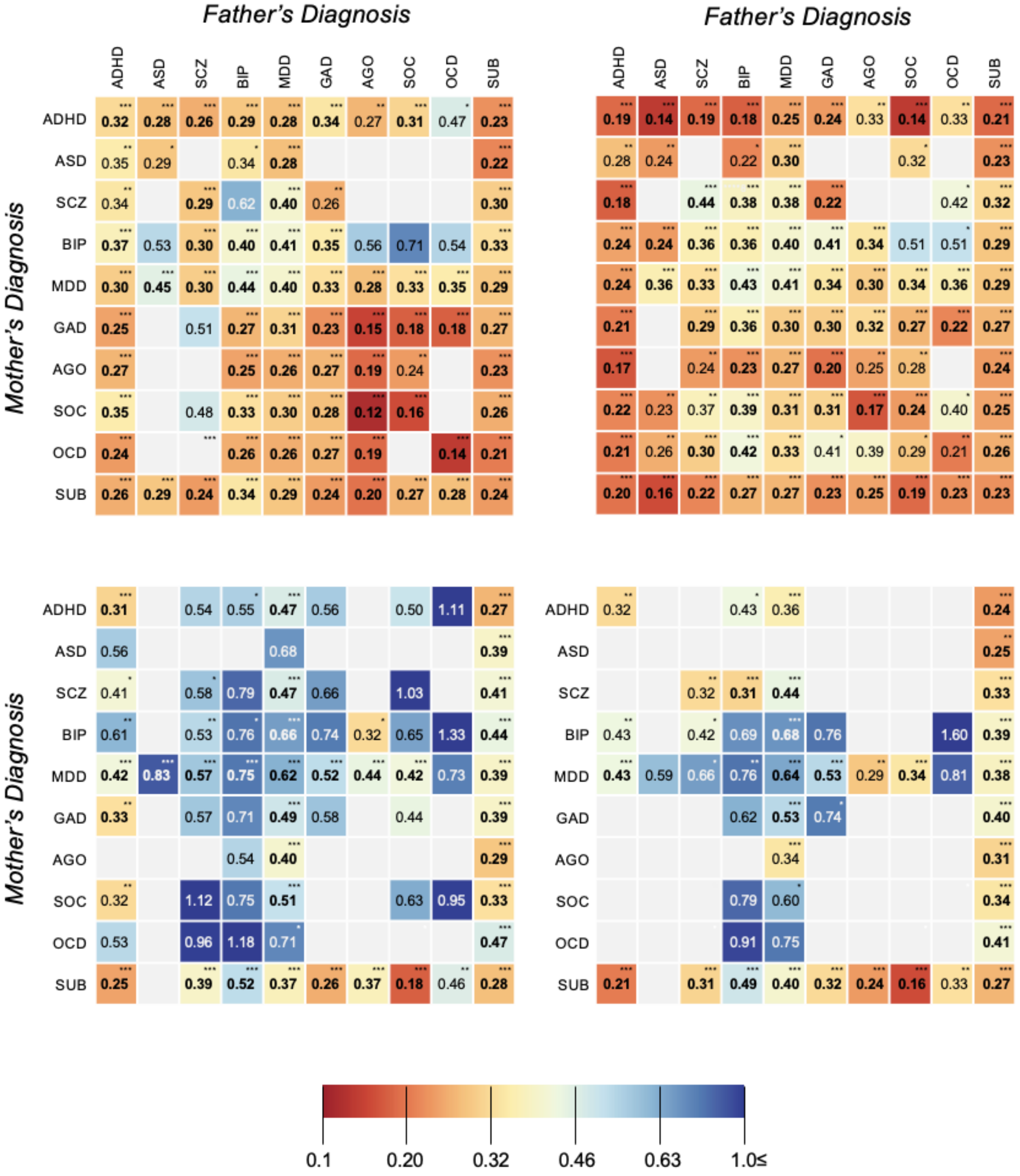

Fig. S7. <u>Sensitivity analysis #3 (if both parents neither died before 1997 nor emigrated prior to 1997and never return to Sweden)</u>: educational outcomes in offspring of parents affected within-disorder and cross-disorder. Adjusted ORs and p-values (# p-value <0.05; § p-value <0.01; * p-value <0.001) for achieving (**A**) compulsory educational outcome, (**B**) finishing USS, (**C**) starting university, and (**D**) finishing university, compared with individuals with parents from the general population (*uncleaned population*[a]).

Note: All models are adjusted for offspring year of birth (5-year categories starting from 1973), offspring sex, and maternal and paternal year of birth in decades (<1940, 1950s, 1960s, 1970s, ≥1970) and clustered by family identification number with robust standard error estimation (sandwich estimator). *mXXX* denotes maternal diagnoses and *fXXX* denotes paternal diagnoses. '*Within-disorder affected parental pairs'* imply that both parents have records of the same disorder. '*Cross-disorder affected parental pairs'* imply that parents have records of different disorders

out of 11 psychiatric disorders in question. For each educational outcome, separate sub-cohorts of index offspring were created, comprising the individuals who had the time necessary to achieve the corresponding outcome. Empty cells indicate the results of analyses with no exposed individuals or if ≤5 exposed cases or exposed non-cases were available (as a result, Tourette's disorder and chronic tic disorders are not at all reported as maternal or paternal diagnoses due to small numbers of exposed cases or non-cases). Figures in bold denote the statistically significant associations and figures in Italics denote non-significant associations.

[a] 'Uncleaned population' imposes no restriction on parental diagnoses in the comparison group and represents, in each relevant analysis, the rest of study population. This approach reflects the high rates of psychiatric comorbidity and ensures obtaining real-world, conservative measures of associations.

Abbreviation: ADHD, attention-deficit hyperactivity disorder; AGO, agoraphobia; ASD, autism spectrum disorder; BIP, bipolar disorder; GAD, generalized anxiety disorder; MDD, major depressive disorder; OCD, obsessive-compulsive disorder; SCZ, schizophrenia; SOC, social phobia; SUD, substance use disorder

Table S1A. Study Design: List of Swedish International Classification of Diseases (ICD) codes to collect records of parental and offspring psychiatric disorders in the National Patient Register.

Diagnoses	ICD-8 codes[a]	ICD-9 codes[a]	ICD-10 codes[a]	Minimal age
Attention-deficit/hyperactivity disorder[c]		314W, 314X	F90<br>Prescribed Drug Register codes[b]: N06BA01, N06BA02, N06BA04, N06BA09	If recorded at age 3 years or above
Autism spectrum disorders		299A	F84.0, F84.1, F84.5, F84.8, F84.9	If recorded at age ≥1 year
Obsessive-compulsive disorder	300.3	300D	F42	If recorded at age ≥6 years
Tourette's and chronic tic disorders[d]	306.2	307C	F95	If recorded at age ≥3 years
Schizophrenia[e]	295 (excluding 295.5)	295 (excluding 295F)	F20, F23.1, F23.2, F25	If recorded at age ≥10 years
Bipolar disorder[e]	296 (excluding 296.2)	296 (excluding 296B)	F30, F31	If recorded at age ≥10 years
Major depression disorder	296.0, 296.2	296B	F32, F33	If recorded at age ≥10 years
Generalized anxiety disorder			F41.1	If recorded at age ≥6 years
Agoraphobia			F40.0	If recorded at age ≥6 years
Social phobia			F40.1	If recorded at age ≥6 years
Substance use disorders	303, 304	303, 304, 305A, 305X	F10-F19 (except subsection .5)	If recorded at age ≥10 years

[a] In the National Patient Register, the diagnoses are coded according to the Swedish version of ICD-codes in its 8th, 9th, or 10th revision.
[b] In the Prescribed Drug Register, drug classes are defined following the Anatomical Therapeutic Chemical (ATC) classification system codes.
[c] Diagnosis defined through both ICD-code and prescription of drugs for attention-deficit/hyperactivity disorder as recorded in the Prescribed Drug Register by the corresponding ATC-codes. The validity of this definition has been previously described *(Larsson et al., 2013)*.
[d] The Swedish codes for Tourette's disorder and chronic tic disorders have been previously validated *(Ruck et al., 2015)* and a previously described algorithm has been used to minimize the inclusion of individuals with only transient tics *(Brander et al., 2018; Fernandez de la Cruz et al., 2017; Mataix-Cols et al., 2015; Perez-Vigil et al., 2018)*.
[e] For sensitivity analysis #2, stricter definition of parental schizophrenia and bipolar disorder have been used that required at least two discharge diagnoses of a corresponding disorder to be recorded (offspring diagnoses were left unchanged).

Table S1B. Study Design: The RECORD Statement – Checklist of Items to be reported in observational studies using routinely collected health data *(Benchimol et al., 2015)*

	Item No.	STROBE items	Location in manuscript where items are reported	RECORD items	Location in manuscript where items are reported
**Title and abstract**					
	1	(a) Indicate the study's design with a commonly used term in the title or the abstract (b) Provide in the abstract an informative and balanced summary of what was done and what was found	Abstract	RECORD 1.1: The type of data used should be specified in the title or abstract. When possible, the name of the databases used should be included.<br><br>RECORD 1.2: If applicable, the geographic region and timeframe within which the study took place should be reported in the title or abstract.<br><br>RECORD 1.3: If linkage between databases was conducted for the study, this should be clearly stated in the title or abstract.	1.1-1.3: Abstract
**Introduction**					
Background rationale	2	Explain the scientific background and rationale for the investigation being reported	Pages 1-2		Pages 1-2
Objectives	3	State specific objectives, including any prespecified hypotheses	Page 2, Final Paragraph of Intro		Page 2, Final Paragraph of Intro
**Methods**					
Study Design	4	Present key elements of study design early in the paper	Page 2 (Final Paragraph), Page 3 Methods		Page 2 (Final Paragraph), Page 3 Methods
Setting	5	Describe the setting, locations, and relevant dates, including periods of recruitment, exposure, follow-up, and data collection	Page 3, Study Population		Page 3, Study Population

Participants	6	*(a) Cohort study* - Give the eligibility criteria, and the sources and methods of selection of participants. Describe methods of follow-up<br>*Case-control study* - Give the eligibility criteria, and the sources and methods of case ascertainment and control selection. Give the rationale for the choice of cases and controls<br>*Cross-sectional study* - Give the eligibility criteria, and the sources and methods of selection of participants<br><br>*(b) Cohort study* - For matched studies, give matching criteria and number of exposed and unexposed<br>*Case-control study* - For matched studies, give matching criteria and the number of controls per case	A. Page 3, Study Cohorts	RECORD 6.1: The methods of study population selection (such as codes or algorithms used to identify subjects) should be listed in detail. If this is not possible, an explanation should be provided.<br><br>RECORD 6.2: Any validation studies of the codes or algorithms used to select the population should be referenced. If validation was conducted for this study and not published elsewhere, detailed methods and results should be provided.<br><br>RECORD 6.3: If the study involved linkage of databases, consider use of a flow diagram or other graphical display to demonstrate the data linkage process, including the number of individuals with linked data at each stage.	6.1 Pages 3-4<br><br>6.2 Page 3, Exposures<br><br>6.3 A flow diagram has been introduced in the Supplemental Materials
Variables	7	Clearly define all outcomes, exposures, predictors, potential confounders, and effect modifiers. Give diagnostic criteria, if applicable.	Pages 3&4, Exposure/Outcome definitions; Also Supplemental Table S1, Figures S2, S3	RECORD 7.1: A complete list of codes and algorithms used to classify exposures, outcomes, confounders, and effect modifiers should be provided. If these cannot be reported, an explanation should be provided.	Pages 3&4, Exposure/Outcome definitions; Also Supplemental Table S1, Figures S2, S3
Data sources/ measurement	8	For each variable of interest, give sources of data and details of methods of assessment (measurement).<br>Describe comparability of assessment methods if there is more than one group	Pages 3-5; Supplemental Table S1, Figures S2, S3		Pages 3-5; Supplemental Table S1, Figures S2, S3

Bias	9	Describe any efforts to address potential sources of bias	Page 3, Study Population; Page 10, Study Limitations		Page 3, Study Population; Page 10, Study Limitations
Study size	10	Explain how the study size was arrived at	Page 3 Study Population		Page 3, Study Population
Quantitative variables	11	Explain how quantitative variables were handled in the analyses. If applicable, describe which groupings were chosen, and why	Pages 3-6, covering definition of all exposure/outcome variables and statistical approach to their analyses.		Pages 3-6, covering definition of all exposure/outcome variables and statistical approach to their analyses.
Statistical methods	12	(a) Describe all statistical methods, including those used to control for confounding<br>(b) Describe any methods used to examine subgroups and interactions<br>(c) Explain how missing data were addressed<br>(d) *Cohort study* - If applicable, explain how loss to follow-up was addressed<br>*Case-control study* - If applicable, explain how matching of cases and controls was addressed<br>*Cross-sectional study* - If applicable, describe analytical methods taking account of sampling strategy<br>(e) Describe any sensitivity analyses	All content contained on pages 5 and 6, under Statistical analysis and Sensitivity analysis sub-sections		All content contained on pages 5 and 6, under Statistical analysis and Sensitivity analysis sub-sections

Data access and cleaning methods		..		RECORD 12.1: Authors should describe the extent to which the investigators had access to the database population used to create the study population.	Page 3; Swedish National Registers sub-section
				RECORD 12.2: Authors should provide information on the data cleaning methods used in the study.	Page 3; Study Population and Sub-cohorts; Supplemental Figure S1A&B
Linkage		..		RECORD 12.3: State whether the study included person-level, institutional-level, or other data linkage across two or more databases. The methods of linkage and methods of linkage quality evaluation should be provided.	Page 3; Swedish National Registers sub-section
**Results**					
Participants	13	(a) Report the numbers of individuals at each stage of the study (*e.g.*, numbers potentially eligible, examined for eligibility, confirmed eligible, included in the study, completing follow-up, and analysed)<br>(b) Give reasons for non-participation at each stage.<br>(c) Consider use of a flow diagram	We have introduced a flow diagram, alongside a table outlining the data structure (see Supplemental Figures 1A& B); selection is also covered, in text, on Page 3 Study Population and Sub-Cohorts sub-section	RECORD 13.1: Describe in detail the selection of the persons included in the study (*i.e.,* study population selection) including filtering based on data quality, data availability and linkage. The selection of included persons can be described in the text and/or by means of the study flow diagram.	We have introduced a flow diagram, alongside a table outlining the data structure (see Supplemental Figures 1A& B); selection is also covered, in text, on Page 3 Study Population and Sub-Cohorts sub-section

Descriptive data	14	(a) Give characteristics of study participants (*e.g.*, demographic, clinical, social) and information on exposures and potential confounders<br>(b) Indicate the number of participants with missing data for each variable of interest<br>(c) *Cohort study* - summarise follow-up time (*e.g.*, average and total amount)	Page 3		Page 3
Outcome data	15	*Cohort study* - Report numbers of outcome events or summary measures over time<br>*Case-control study* - Report numbers in each exposure category, or summary measures of exposure<br>*Cross-sectional study* - Report numbers of outcome events or summary measures	All cohort members have data on the universal outcome under study (educational attainment); thus no numbers are presented outside of the context of main results.		All cohort members have data on the universal outcome under study (educational attainment); thus no numbers are presented outside of the context of main results.

Main results	16	(a) Give unadjusted estimates and, if applicable, confounder-adjusted estimates and their precision (e.g., 95% confidence interval). Make clear which confounders were adjusted for and why they were included<br>(b) Report category boundaries when continuous variables were categorized<br>(c) If relevant, consider translating estimates of relative risk into absolute risk for a meaningful time period	Pages 6 & 7, all Tables/Figures referenced therein.		Pages 6 & 7, all Tables/Figures referenced therein.
Other analyses	17	Report other analyses done—e.g., analyses of subgroups and interactions, and sensitivity analyses	Page 7, Sensitivity Analysis Sub-Section (and all Figures/Tables referenced therein)		Page 7, Sensitivity Analysis Sub-Section (and all Figures/Tables referenced therein)
**Discussion**					
Key results	18	Summarise key results with reference to study objectives			
Limitations	19	Discuss limitations of the study, taking into account sources of potential bias or imprecision. Discuss both direction and magnitude of any potential bias	Page 9/10 Strengths and Limitations	RECORD 19.1: Discuss the implications of using data that were not created or collected to answer the specific research question(s). Include discussion of misclassification bias, unmeasured confounding, missing data, and changing eligibility over time, as they pertain to the study being reported.	Page 9/10 Strengths & Limitations
Interpretation	20	Give a cautious overall interpretation of results considering objectives,	Page 9 Discussion; Page 10, Conclusions		Page 9 Discussion, Page 10, Conclusions

		limitations, multiplicity of analyses, results from similar studies, and other relevant evidence			
Generalisability	21	Discuss the generalisability (external validity) of the study results			
**Other Information**					
Funding	22	Give the source of funding and the role of the funders for the present study and, if applicable, for the original study on which the present article is based	Page 10, Funding		Page 10, Funding
Accessibility of protocol, raw data, and programming code		..	Page 10, Additional Information	RECORD 22.1: Authors should provide information on how to access any supplemental information such as the study protocol, raw data, or programming code.	Page 10, Additional Information

Table S2. Educational outcomes in offspring with parents single-affected by each specific disorder (one disorder at the time). Adjusted OR (95% CI) for achieving each educational outcome in all index offspring and offspring free from any 11 psychiatric disorders with parents single-affected by each specific disorder (one disorder at the time), compared to individuals with parents from the general population (*uncleaned population*[a]).

	All offspring			Offspring free from any 11 psychiatric disorders		
	**Offspring of affected pairs**	**Offspring of unaffected pairs**	**Fully-adjusted**[b]	**Offspring of affected pairs**	**Offspring of unaffected pairs**	**Fully-adjusted**[b]
	**n (%)**	**n (%)**	**OR (95% CI)**	**n (%)**	**n (%)**	**OR (95% CI)**
**ELIGIBILITY TO ACCESS USS**[c]						
ADHD	15,824 (78.30)	1,277,561 (91.47)	0.38 (0.37-0.40)*	10,770 (85.12)	1,152,263 (93.07)	0.48 (0.45-0.50)*
ASD	2,139 (80.14)	1,291,246 (91.31)	0.43 (0.39-0.48)*	1,427 (87.82)	1,161,606 (92.99)	0.60 (0.51-0.70)*
SCZ	5,133 (83.23)	1,288,252 (91.32)	0.48 (0.44-0.51)*	4,093 (86.46)	1,158,940 (93.01)	0.49 (0.45-0.53)*
BIP	24,394 (86.82)	1,268,991 (91.38)	0.64 (0.61-0.66)*	19,421 (90.18)	1,143,612 (93.04)	0.70 (0.67-0.74)*
MDD	111,446 (85.90)	1,181,939 (91.83)	0.56 (0.55-0.57)*	91,223 (89.22)	1,071,810 (93.32)	0.61 (0.60-0.63)*
GAD	14,250 (83.63)	1,279,135 (91.38)	0.51 (0.48-0.53)*	11,447 (87.86)	1,151,586 (93.04)	0.57 (0.54-0.60)*
AGO	5,129 (80.02)	1,288,256 (91.34)	0.42 (0.39-0.45)*	4,099 (84.88)	1,158,934 (93.02)	0.46 (0.42-0.50)*
SOC	7,408 (80.50)	1,285,977 (91.36)	0.43 (0.40-0.45)*	5,882 (84.79)	1,157,151 (93.03)	0.45 (0.42-0.49)*
OCD	5,728 (85.07)	1,287,657 (91.31)	0.58 (0.54-0.63)*	4,599 (89.60)	1,158,434 (93.00)	0.69 (0.63-0.76)*
SUD	99,839 (82.33)	1,193,546 (92.12)	0.41 (0.40-0.42)*	82,293 (85.78)	1,080,740 (93.59)	0.43 (0.42-0.44)*
TD/CTD	450 (82.27)	1,292,935 (91.29)	0.49 (0.38-0.63)*	358 (89.95)	1,162,675 (92.99)	0.73 (0.53-1.02)(*)
**FINISHING USS**[d]						
ADHD	10,784 (55.29)	1,589,126 (80.83)	0.36 (0.34-0.37)*	8,388 (67.61)	1,467,326 (84.00)	0.47 (0.45-0.49)*
ASD	1,513 (58.08)	1,598,397 (80.61)	0.40 (0.37-0.44)*	1,139 (70.48)	1,474,575 (83.90)	0.54 (0.48-0.61)*
SCZ	7,135 (67.78)	1,592,775 (80.65)	0.49 (0.47-0.52)*	6.042 (74.68)	1,469,672 (83.93)	0.56 (0.53-0.59)*
BIP	27,947 (71.56)	1,571,963 (80.76)	0.61 (0.59-0.63)*	23,748 (78.22)	1,451,966 (83.99)	0.69 (0.67-0.72)*
MDD	123,853 (71.21)	1,476,057 (81.48)	0.58 (0.57-0.59)*	107,613 (77.43)	1,368,101 (84.44)	0.65 (0.64-0.66)*
GAD	14,475 (67.45)	1,585,435 (80.72)	0.53 (0.51-0.55)*	12,391 (74.91)	1,463,323 (83.97)	0.61 (0.58-0.63)*

AGO	4,406 (61.02)	1,595,504 (80.65)	0.43 (0.41-0.46)[*]	3,758 (68.88)	1,471,956 (83.93)	0.48 (0.45-0.51)[*]
SOC	6,305 (61.73)	1,593,605 (80.68)	0.45 (0.43-0.47)[*]	5,352 (69.62)	1,470,362 (83.95)	0.50 (0.47-0.52)[*]
OCD	5,495 (69.56)	1,594,415 (80.62)	0.60 (0.57-0.63)[*]	4,646 (76.73)	1,471,068 (83.91)	0.68 (0.64-0.73)[*]
SUD	124,865 (66.21)	1,475,045 (82.09)	0.42 (0.42-0.43)[*]	109,430 (72.29)	1,366,284 (84.98)	0.46 (0.45-0.47)[*]
TD/CTD	393 (64.85)	1,599,517 (80.58)	0.49 (0.41-0.60)[*]	341 (76.29)	1,475,373 (83.89)	0.67 (0.53-0.85)[*]
**STARING UNIVERSITY[e]**						
ADHD	3,339 (21.08)	680,951 (38.57)	0.57 (0.54-0.59)[*]	2,654 (25.95)	630,755 (40.23)	0.68 (0.64-0.71)[*]
ASD	507 (23.83)	683,783 (38.44)	0.65 (0.58-0.73)[*]	405 (30.22)	633,004 (40.14)	0.83 (0.73-0.95)[§]
SCZ	3,063 (31.33)	681,227 (38.46)	0.71 (0.68-0.75)[*]	2,605 (34.67)	630,804 (40.16)	0.77 (0.73-0.82)[*]
BIP	12,323 (35.22)	671,967 (38.48)	0.88 (0.86-0.91)[*]	10,488 (38.52)	622,921 (40.16)	0.94 (0.92-0.97)[*]
MDD	49,323 (31.87)	634,967 (39.04)	0.76 (0.75-0.77)[*]	43,109 (34.78)	590,300 (40.59)	0.81 (0.80-0.82)[*]
GAD	5,395 (28.54)	678,895 (38.52)	0.70 (0.67-0.72)[*]	4,665 (32.03)	628,744 (40.21)	0.76 (0.73-0.79)[*]
AGO	1,380 (22.18)	682,910 (38.48)	0.54 (0.51-0.59)[*]	1,183 (25.06)	632,226 (40.18)	0.59 (0.55-0.64)[*]
SOC	2,037 (23,36)	682,253 (38.49)	0.59 (0.56-0.63)[*]	1,744 (26.52)	631,665 (40.19)	0.64 (0.61-0.68)[*]
OCD	2,160 (31.52)	682,130 (38.45)	0.82 (0.77-0.87)[*]	1,838 (34.97)	631,571 (40.15)	0.89 (0.83-0.95)[*]
SUD	43,800 (25.38)	640,490 (39.82)	0.51 (0.50-0.51)[*]	38,668 (27.88)	594,741 (41.32)	0.54 (0.53-0.55)[*]
TD/CTD	157 (29.96)	684,133 (38.42)	0.76 (0.61-0.95)[#]	139 (35.82)	633,270 (40.14)	0.91 (0.71-1.16)[(*)]
**FINISHING UNIVERSITY[f]**						
ADHD	1,199 (13.06)	347,804 (26.05)	0.58 (0.54-0.62)[*]	1,005 (16.45)	326,935 (27.50)	0.69 (0.64-0.74)[*]
ASD	172 (13.36)	348,831 (25.98)	0.59 (0.50-0.70)[*]	145 (17.22)	327,795 (27.45)	0.73 (0.60-0.88)[§]
SCZ	1,489 (19.07)	347,514 (26.00)	0.67 (0.63-0.71)[*]	1,316 (21.91)	326,624 (27.47)	0.74 (0.69-0.79)[*]
BIP	6,205 (23.79)	342,798 (26.01)	0.90 (0.87-0.93)[*]	5,441 (26.38)	322,499 (27.46)	0.96 (0.93-0.99)[#]
MDD	24,066 (21.21)	324,937 (26.40)	0.78 (0.77-0.79)[*]	21,655 (23.56)	306,285 (27.77)	0.83 (0.82-0.85)[*]
GAD	2,482 (18.36)	346,521 (26.04)	0.70 (0.67-0.74)[*]	2,205 (20.99)	325,735 (27.50)	0.77 (0.73-0.81)[*]
AGO	557 (13.57)	348,446 (26.00)	0.54 (0.49-0.60)[*]	498 (15.94)	327,442 (27.48)	0.60 (0.54-0.66)[*]
SOC	843 (14.71)	348,160 (26.01)	0.60 (0.56-0.66)[*]	746 (17.18)	327,194 (27.48)	0.67 (0.61-0.73)[*]

OCD	1,007 (21.35)	347,996 (25.98)	0.84 (0.78-0.91)*	888 (24.37)	327,052 (27.45)	0.92 (0.85-1.00)(*)
SUD	21,324 (15.85)	327,679 (27.09)	0.51 (0.50-0.52)*	19,366 (17.77)	308,574 (28.42)	0.54 (0.53-0.55)*
TD/CTD	62 (17.61)	348,941 (25.97)	0.67 (0.50-0.90)§	57 (21.59)	327,883 (27.45)	0.78 (0.57-1.06)(*)

Note: All models are clustered by family identification number with robust standard error estimation (sandwich estimator). For each disorder, *'single-affected parental pairs'* imply that one parent (either mother or father) has a disorder in question and the other parent is free from such disorder. For each educational outcome, separate sub-cohorts of index offspring were created, comprising the individuals who had the time necessary to achieve the corresponding outcome.

[a] 'Uncleaned population' imposes no restriction on parental diagnoses in the comparison group and represents, in each relevant analysis, the rest of study population. This approach reflects the high rates of psychiatric comorbidity and ensures obtaining real-world, conservative measures of associations.
[b] Adjusted for offspring year of birth (5-year categories starting from 1973), offspring sex, and maternal and paternal year of birth in decades (<1940, 1950s, 1960s, 1970s, ≥1970)
[c] Offspring who graduated from compulsory school in 1998-2013 (all offspring: n=1,416,867; offspring free from any 11 psychiatric disorders: n=1,250,741). The graduation years were chosen due to the different eligibility criteria used prior to 1998.
[d] Offspring born in 1973-1994 (i.e., aged ≥19 by the end of follow-up) and who was alive and living in Sweden at age 19 years (all offspring: n=1,985,519; offspring free from any 11 psychiatric disorders: n=1,759,185).
[e] Offspring born in 1973-1992 (i.e., aged ≥21 by the end of follow-up) and who was alive and living in Sweden at age 21 years (all offspring: n=1,781,145; offspring free from any 11 psychiatric disorders: n=1,578,177).
[f] Offspring born in 1973-1988 (i.e., aged ≥25 by the end of follow-up) and who was alive and living in Sweden at age 25 years (all offspring: n=1,344,192; offspring free from any 11 psychiatric disorders: n=1,194,891).

(*) p-value ≥0.05; # p-value <0.05; § p-value <0.01; * p-value <0.001

Abbreviation: ADHD, attention-deficit hyperactivity disorder; AGO, agoraphobia; ASD, autism spectrum disorder; BIP, bipolar disorder; CI, confidence interval; GAD, generalized anxiety disorder; MDD, major depressive disorder; OCD, obsessive-compulsive disorder; OR, odds ratio; SCZ, schizophrenia; SOC, social phobia; SUD, substance use disorders; TD/CTD, Tourette's disorder and chronic tic disorders; USS, upper secondary school.

Table S3. Sensitivity Analysis #4A (adjustment and mediation for parental education level): Completion of compulsory (primary) school/eligibility to access USS among offspring who graduated 1998-2013

	Parental Affected Status	Main Fully-Adjusted Model	Additional Adjustment for Highest Parent Education Level	Mediation: % Main Effect Represented by Indirect Effect
		OR (95% CI), p-value	OR (95% CI), p-value	%
**Parents affected by any of 11 psychiatric disorders**				
Main analysis: comparison with 'uncleaned population'	Dual	0.29 (0.28-0.30), p<0.001	0.39 (0.38-0.40), p<0.001	24.2
Main analysis: comparison with 'uncleaned population'	Single	0.50 (0.49-0.51), p<0.001	0.57 (0.56-0.58), p<0.001	19.4
Main analysis: comparison with 'uncleaned population' *in offspring free from any 11 diagnoses*	Dual	0.33 (0.31-0.34), p<0.001	0.44 (0.42-0.46), p<0.001	26.5
Main analysis: comparison with 'uncleaned population' *in offspring free from any 11 diagnoses*	Single	0.53 (0.52-0.54), p<0.001	0.61 (0.60-0.62), p<0.001	25.4
Sensitivity analysis #1: comparison with 'cleaned population'	Dual	0.25 (0.24-0.26), p<0.001	0.34 (0.33-0.35), p<0.001	22.5
Sensitivity analysis #1: comparison with 'cleaned population'	Single	0.48 (0.47-0.48), p<0.001	0.54 (0.53-0.55), p<0.001	18.7
Sensitivity analysis #2: with repeated SCZ and BIP	Dual	0.29 (0.28-0.30), p<0.001	0.39 (0.37-0.40), p<0.001	24.2
Sensitivity analysis #2: with repeated SCZ and BIP	Single	0.50 (0.49-0.51), p<0.001	0.57 (0.56-0.58), p<0.001	19.5
Sensitivity analysis #3: if both parents neither died before 1997 nor emigrated prior to 1997and never return to Sweden	Dual	0.30 (0.29-0.31), p<0.001	0.39 (0.38-0.40), p<0.001	23.7
Sensitivity analysis #3: if both parents neither died before 1997 nor emigrated prior to 1997and never return to Sweden	Single	0.50 (0.49-0.51), p<0.001	0.57 (0.56-0.58), p<0.001	19.1
**Parents affected by five groups of psychiatric disorders (one group at the time)**				
***Main analysis* in the whole cohort**				
Neuropsychiatric disorders	Dual	0.30 (0.25-0.37), p<0.001	0.40 (0.32-0.49), p<0.001	24.3
SUD	Dual	0.24 (0.23-0.25), p<0.001	0.36 (0.34-0.38), p<0.001	27.9
GAD and MDD	Dual	0.38 (0.36-0.41), p<0.001	0.45 (0.42-0.48), p<0.001	19.2
AGO, SOC, and OCD	Dual	0.24 (0.17-0.34), p<0.001	0.36 (0.25-0.53), p<0.001	31.3
SCZ and BIP	Dual	0.40 (0.31-0.52), p<0.001	0.48 (0.37-0.62), p<0.001	19.5
Neuropsychiatric disorders	Single	0.39 (0.37-0.40), p<0.001	0.45 (0.43-0.47), p<0.001	18.7

SUD	Single	0.41 (0.40-0.42), p<0.001	0.50 (0.49-0.51), p<0.001	23.8
GAD and MDD	Single	0.56 (0.55-0.57), p<0.001	0.61 (0.60-0.62), p<0.001	16.7
AGO, SOC, and OCD	Single	0.48 (0.46-0.50), p<0.001	0.57 (0.55-0.59), p<0.001	25.8
SCZ and BIP	Single	0.61 (0.59-0.63), p<0.001	0.65 (0.63-0.68), p<0.001	14.7
***Main analysis* in offspring free from any 11 diagnoses**				
Neuropsychiatric disorders	Dual	0.52 (0.36-0.75), p<0.001	0.71 (0.49-1.02), p=0.069	44.1
SUD	Dual	0.27 (0.25-0.29), p<0.001	0.41 (0.38-0.43), p<0.001	30.4
GAD and MDD	Dual	0.44 (0.40-0.48), p<0.001	0.52 (0.48-0.57), p<0.001	21.6
AGO, SOC, and OCD	Dual	0.27 (0.16-0.44), p<0.001	0.42 (0.25-0.71), p=0.001	35.8
SCZ and BIP	Dual	0.37 (0.27-0.52), p<0.001	0.45 (0.32-0.62), p<0.001	16.3
Neuropsychiatric disorders	Single	0.49 (0.46-0.51), p<0.001	0.57 (0.54-0.60), p<0.001	23.7
SUD	Single	0.43 (0.42-0.44), p<0.001	0.53 (0.51-0.54), p<0.001	25.2
GAD and MDD	Single	0.61 (0.59-0.62), p<0.001	0.67 (0.65-0.68), p<0.001	18.9
AGO, SOC, and OCD	Single	0.53 (0.50-0.55), p<0.001	0.63 (0.59-0.66), p<0.001	28.5
SCZ and BIP	Single	0.66 (0.63-0.69), p<0.001	0.70 (0.67-0.74), p<0.001	15.5
***Sensitivity analysis #1 (C*omparison with 'cleaned population')**				
Neuropsychiatric disorders	Dual	0.25 (0.20-0.30), p<0.001	0.33 (0.27-0.41), p<0.001	22.2
SUD	Dual	0.20 (0.19-0.21), p<0.001	0.31 (0.29-0.32), p<0.001	25.8
GAD and MDD	Dual	0.32 (0.30-0.34), p<0.001	0.38 (0.36-0.41), p<0.001	18.0
AGO, SOC, and OCD	Dual	0.20 (0.14-0.28), p<0.001	0.30 (0.21-0.44), p<0.001	28.3
SCZ and BIP	Dual	0.33 (0.26-0.43), p<0.001	0.41 (0.32-0.52), p<0.001	18.2
Neuropsychiatric disorders	Single	0.33 (0.31-0.34), p<0.001	0.39 (0.38-0.41), p<0.001	17.7
SUD	Single	0.38 (0.37-0.38), p<0.001	0.47 (0.46-0.48), p<0.001	22.5
GAD and MDD	Single	0.49 (0.48-0.50), p<0.001	0.55 (0.54-0.56), p<0.001	16.2
AGO, SOC, and OCD	Single	0.40 (0.38-0.42), p<0.001	0.49 (0.47-0.51), p<0.001	23.0
SCZ and BIP	Single	0.51 (0.49-0.53), p<0.001	0.56 (0.54-0.58), p<0.001	14.5
***Sensitivity analysis #2 (R*epeated SCZ and BIP Diagnosis)**				
Repeated SCZ and BIP	Dual	0.49 (0.36-0.66), p<0.001	0.58 (0.42-0.80), p=0.001	23.7
Repeated SCZ and BIP	Single	0.62 (0.59-0.64), p<0.001	0.66 (0.63-0.68), p<0.001	13.8
***Sensitivity analysis #3*: if both parents neither died before 1997 nor emigrated prior to 1997and never return to Sweden**				
Neuropsychiatric disorders	Dual	0.31 (0.26-0.39), p<0.001	0.42 (0.34-0.52), p<0.001	26.3
SUD	Dual	0.24 (0.23-0.25), p<0.001	0.36 (0.34-0.38), p<0.001	27.7

GAD and MDD	Dual	0.38 (0.36-0.41), p<0.001	0.42 (0.42-0.48), p<0.001	19.0
AGO, SOC, and OCD	Dual	0.24 (0.17-0.35), p<0.001	0.37 (0.25-0.54), p<0.001	31.9
SCZ and BIP	Dual	0.39 (0.30-0.51), p<0.001	0.47 (0.36-0.61), p<0.001	18.4
Neuropsychiatric disorders	Single	0.39 (0.37-0.40), p<0.001	0.45 (0.43-0.47), p<0.001	18.5
SUD	Single	0.41 (0.40-0.42), p<0.001	0.50 (0.49-0.51), p<0.001	23.5
GAD and MDD	Single	0.56 (0.55-0.57), p<0.001	0.61 (0.60-0.62), p<0.001	16.5
AGO, SOC, and OCD	Single	0.48 (0.46-0.51), p<0.001	0.58 (0.55-0.60), p<0.001	25.7
SCZ and BIP	Single	0.61 (0.59-0.63), p<0.001	0.65 (0.63-0.68), p<0.001	14.1

Note: Columns highlighted in orange indicate main effect moved from significant to non-significant on adjustment for parental education level

Abbreviations: ADHD, attention-deficit hyperactivity disorder; AGO, agoraphobia; ASD, autism spectrum disorder; BIP, bipolar disorder; CI, confidence intervals; GAD, generalized anxiety disorder; MDD, major depressive disorder; OCD, obsessive-compulsive disorder; OR, odds ratio; SCZ, schizophrenia; SOC, social phobia; SUD, substance use disorders; TD/CTD, Tourette's disorder and chronic tic disorders; USS, upper secondary school.

Table S4 **Sensitivity Analysis #4B (adjustment and mediation for parental education level):** Completion of USS among Swedish resident offspring born in 1973-1994 (i.e., aged ≥19 by the end of follow-up)

	Parental Affected Status	Main Fully-Adjusted Model	Additional Adjustment for Highest Parent Education Level	Mediation: % Main Effect Represented by Indirect Effect
		OR (95% CI)	OR (95% CI), p-value	%
**Parents affected by any of 11 psychiatric disorders**				
Main analysis: comparison with 'uncleaned population'	Dual	0.30 (0.28-0.31), p<0.001	0.35 (0.34-0.36), p<0.001	13.6
Main analysis: comparison with 'uncleaned population'	Single	0.52 (0.51-0.52), p<0.001	0.55 (0.54-0.56), p<0.001	10.9
Main analysis: comparison with 'uncleaned population' *in offspring free from any 11 diagnoses*	Dual	0.36 (0.35-0.37), p<0.001	0.42 (0.41-0.43), p<0.001	15.7
Main analysis: comparison with 'uncleaned population' *in offspring free from any 11 diagnoses*	Single	0.56 (0.55-0.57), p<0.001	0.60 (0.59-0.61), p<0.001	12.3
Sensitivity analysis #1: comparison with 'cleaned population'	Dual	0.26 (0.25-0.27), p<0.001	0.30 (0.29-0.31), p<0.001	12.3
Sensitivity analysis #1: comparison with 'cleaned population'	Single	0.49 (0.49-0.50), p<0.001	0.53 (0.52-0.53), p<0.001	10.4
Sensitivity analysis #2: with repeated SCZ and BIP	Dual	0.30 (0.29-0.30), p<0.001	0.34 (0.33-0.35), p<0.001	13.7
Sensitivity analysis #2: with repeated SCZ and BIP	Single	0.51 (0.51-0.52), p<0.001	0.55 (0.54-0.56), p<0.001	11.0
Sensitivity analysis #3: if both parents neither died before 1997 nor emigrated prior to 1997and never return to Sweden	Dual	0.30 (0.29-0.31), p<0.001	0.34 (0.33-0.35), p<0.001	13.0
Sensitivity analysis #3: if both parents neither died before 1997 nor emigrated prior to 1997and never return to Sweden	Single	0.51 (0.51-0.52), p<0.001	0.55 (0.54-0.56), p<0.001	10.5
**Parents affected by five groups of psychiatric disorders (one group at the time)**				
***Main analysis* in the whole cohort**				
Neuropsychiatric disorders	Dual	0.19 (0.16-0.24), p<0.001	0.22 (0.18-0.27), p<0.001	9.1
SUD	Dual	0.24 (0.23-0.25), p<0.001	0.30 (0.28-0.31), p<0.001	16.0
GAD and MDD	Dual	0.41 (0.39-0.43), p<0.001	0.44 (0.41-0.46), p<0.001	9.0
AGO, SOC, and OCD	Dual	0.29 (0.21-0.39), p<0.001	0.38 (0.27-0.52), p<0.001	20.8
SCZ and BIP	Dual	0.40 (0.34-0.47), p<0.001	0.43 (0.37-0.51), p<0.001	8.3
Neuropsychiatric disorders	Single	0.36 (0.35-0.37), p<0.001	0.39 (0.37-0.40), p<0.001	7.0
SUD	Single	0.42 (0.42-0.43), p<0.001	0.48 (0.47-0.48), p<0.001	14.0
GAD and MDD	Single	0.58 (0.57-0.59), p<0.001	0.60 (0.59-0.61), p<0.001	7.4
AGO, SOC, and OCD	Single	0.49 (0.48-0.51), p<0.001	0.54 (0.53-0.56), p<0.001	14.3
SCZ and BIP	Single	0.58 (0.57-0.60), p<0.001	0.60 (0.58-0.61), p<0.001	4.6

***Main analysis* in offspring free from any 11 diagnoses**				
Neuropsychiatric disorders	Dual	0.33 (0.25-0.44), p<0.001	0.38 (0.28-0.51), p<0.001	13.7
SUD	Dual	0.28 (0.27-0.30), p<0.001	0.36 (0.34-0.37), p<0.001	18.0
GAD and MDD	Dual	0.50 (0.47-0.53), p<0.001	0.54 (0.50-0.57), p<0.001	10.8
AGO, SOC, and OCD	Dual	0.41 (0.27-0.63), p<0.001	0.55 (0.35-0.85), p=0.008	28.8
SCZ and BIP	Dual	0.47 (0.38-0.59), p<0.001	0.51 (0.41-0.64), p<0.001	7.9
Neuropsychiatric disorders	Single	0.48 (0.46-0.50), p<0.001	0.51 (0.49-0.53), p<0.001	8.0
SUD	Single	0.46 (0.45-0.46), p<0.001	0.51 (0.50-0.52), p<0.001	15.2
GAD and MDD	Single	0.65 (0.64-0.66), p<0.001	0.67 (0.66-0.68), p<0.001	8.6
AGO, SOC, and OCD	Single	0.55 (0.53-0.57), p<0.001	0.60 (0.58-0.63), p<0.001	15.9
SCZ and BIP	Single	0.66 (0.65-0.68), p<0.001	0.68 (0.66-0.70), p<0.001	4.8
***Sensitivity analysis #1*: comparison with 'cleaned population'**				
Neuropsychiatric disorders	Dual	0.16 (0.13-0.20), p<0.001	0.18 (0.15-0.22), p<0.001	8.2
SUD	Dual	0.20 (0.19-0.21), p<0.001	0.25 (0.24-0.26), p<0.001	14.3
GAD and MDD	Dual	0.34 (0.33-0.36), p<0.001	0.37 (0.35-0.39), p<0.001	8.3
AGO, SOC, and OCD	Dual	0.24 (0.17-0.33), p<0.001	0.31 (0.22-0.43), p<0.001	17.6
SCZ and BIP	Dual	0.34 (0.29-0.40), p<0.001	0.37 (0.31-0.44), p<0.001	7.8
Neuropsychiatric disorders	Single	0.31 (0.30-0.32), p<0.001	0.33 (0.32-0.34), p<0.001	6.7
SUD	Single	0.40 (0.39-0.40), p<0.001	0.44 (0.43-0.45), p<0.001	13.0
GAD and MDD	Single	0.52 (0.51-0.53), p<0.001	0.54 (0.53-0.55), p<0.001	7.6
AGO, SOC, and OCD	Single	0.42 (0.41-0.43), p<0.001	0.46 (0.45-0.48), p<0.001	12.3
SCZ and BIP	Single	0.50 (0.49-0.51), p<0.001	0.52 (0.51-0.53), p<0.001	5.4
***Sensitivity analysis #2*: with repeated SCZ and BIP**				
Repeated SCZ and BIP	Dual	0.39 (0.32-0.48), p<0.001	0.42 (0.54-0.52), p<0.001	8.0
Repeated SCZ and BIP	Single	0.60 (0.58-0.61), p<0.001	0.61 (0.59-0.63), p<0.001	4.2
***Sensitivity analysis #3*: if both parents neither died before 1997 nor emigrated prior to 1997and never return to Sweden**				
Neuropsychiatric disorders	Dual	0.20 (0.16-0.24), p<0.001	0.23 (0.19-0.28), p<0.001	10.2
SUD	Dual	0.23 (0.22-0.24), p<0.001	0.29 (0.28-0.30), p<0.001	15.4
GAD and MDD	Dual	0.40 (0.38-0.42), p<0.001	0.43 (0.40-0.45), p<0.001	9.1
AGO, SOC, and OCD	Dual	0.27 (0.20-0.38), p<0.001	0.36 (0.25-0.40), p<0.001	20.4
SCZ and BIP	Dual	0.38 (0.32-0.45), p<0.001	0.41 (0.34-0.49), p<0.001	7.7
Neuropsychiatric disorders	Single	0.36 (0.35-0.37), p<0.001	0.38 (0.37-0.40), p<0.001	7.0
SUD	Single	0.42 (0.41-0.43), p<0.001	0.47 (0.46-0.48), p<0.001	13.6
GAD and MDD	Single	0.57 (0.56-0.58), p<0.001	0.60 (0.59-0.60), p<0.001	7.4
AGO, SOC, and OCD	Single	0.49 (0.48-0.51), p<0.001	0.54 (0.52-0.56), p<0.001	14.1
SCZ and BIP	Single	0.58 (0.56-0.59), p<0.001	0.59 (0.58-0.60), p<0.001	4.1

Abbreviations: ADHD, attention-deficit hyperactivity disorder; AGO, agoraphobia; ASD, autism spectrum disorder; BIP, bipolar disorder; CI, confidence intervals; GAD, generalized anxiety disorder; MDD, major depressive disorder; OCD, obsessive-compulsive disorder; OR, odds ratio; SCZ, schizophrenia; SOC, social phobia; SUD, substance use disorders; TD/CTD, Tourette's disorder and chronic tic disorders; USS, upper secondary school.

Table S5. <u>Sensitivity Analysis #4C (adjustment and mediation for parental education level):</u> Starting University among Swedish resident offspring born in 1973-1992 (i.e., aged ≥21 by the end of follow-up)

	Parental Affected Status	Main Fully-Adjusted Model	Additional Adjustment for Highest Parent Education Level	Mediation: % Main Effect Represented by Indirect Effect
		OR (95% CI)	OR (95% CI), p-value	%
**Parents affected by any of 11 psychiatric disorders**				
Main analysis: comparison with 'uncleaned population'	Dual	0.40 (0.39-0.42), p<0.001	0.51 (0.49-0.53), p<0.001	33.0
Main analysis: comparison with 'uncleaned population'	Single	0.65 (0.64-0.66), p<0.001	0.71 (0.70-0.72), p<0.001	29.7
Main analysis: comparison with 'uncleaned population' *in offspring free from any 11 diagnoses*	Dual	0.46 (0.44-0.47), p<0.001	0.58 (0.56-0.60), p<0.001	36.9
Main analysis: comparison with 'uncleaned population' *in offspring free from any 11 diagnoses*	Single	0.69 (0.68-0.70), p<0.001	0.75 (0.74-0.76), p<0.001	32.4
Sensitivity analysis #1: comparison with 'cleaned population'	Dual	0.38 (0.36-0.39), p<0.001	0.48 (0.47-0.50), p<0.001	32.3
Sensitivity analysis #1: comparison with 'cleaned population'	Single	0.64 (0.64-0.65), p<0.001	0.71 (0.70-0.71), p<0.001	29.7
Sensitivity analysis #2: with repeated SCZ and BIP	Dual	0.40 (0.39-0.41), p<0.001	0.51 (0.49-0.52), p<0.001	33.0
Sensitivity analysis #2: with repeated SCZ and BIP	Single	0.65 (0.64-0.66), p<0.001	0.71 (0.70-0.72), p<0.001	29.7
Sensitivity analysis #3: if both parents neither died before 1997 nor emigrated prior to 1997and never return to Sweden	Dual	0.41 (0.40-0.42), p<0.001	0.51 (0.50-0.53), p<0.001	32.3
Sensitivity analysis #3: if both parents neither died before 1997 nor emigrated prior to 1997and never return to Sweden	Single	0.66 (0.65-0.66), p<0.001	0.72 (0.71-0.73), p<0.001	29.2
**Parents affected by five groups of psychiatric disorders (one group at the time)**				
***Main analysis* in the whole cohort**				
Neuropsychiatric disorders	Dual	0.34 (0.24-0.49), p<0.001	0.38 (0.27-0.55), p<0.001	22.1
SUD	Dual	0.28 (0.26-0.29), p<0.001	0.40 (0.38-0.42), p<0.001	34.0
GAD and MDD	Dual	0.62 (0.58-0.66), p<0.001	0.67 (0.63-0.71), p<0.001	27.4
AGO, SOC, and OCD	Dual	0.58 (0.37-0.91), p<0.05	0.99 (0.63-1.55), p=0.960	87.7
SCZ and BIP	Dual	0.73 (0.61-0.88), p<0.01	0.84 (0.61-1.02), p=0.085	43.6
Neuropsychiatric disorders	Single	0.58 (0.56-0.61), p<0.001	0.60 (0.58-0.63), p<0.001	19.7
SUD	Single	0.51 (0.50-0.52), p<0.001	0.60 (0.59-0.61), p<0.001	32.4
GAD and MDD	Single	0.76 (0.75-0.77), p<0.001	0.78 (0.77-0.79), p<0.001	23.4
AGO, SOC, and OCD	Single	0.66 (0.64-0.69), p<0.001	0.77 (0.74-0.80), p<0.001	43.6
SCZ and BIP	Single	0.85 (0.83-0.87), p<0.001	0.86 (0.84-0.88), p<0.001	20.2
***Main analysis* in offspring free from any 11 diagnoses**				

Neuropsychiatric disorders	Dual	0.49 (0.30-0.80), p<0.001	0.58 (0.33-0.96), p=0.035	35.8
SUD	Dual	0.33 (0.31-0.35), p<0.001	0.47 (0.45-0.50), p<0.001	37.6
GAD and MDD	Dual	0.68 (0.63-0.73), p<0.001	0.74 (0.69-0.80), p<0.001	32.5
AGO, SOC, and OCD	Dual	0.65 (0.39-1.08), p=0.097	1.16 (0.72-1.89), p=0.538	112.5
SCZ and BIP	Dual	0.85 (0.69-1.05), p=0.134	0.95 (0.74-1.21), p=0.672	59.8
Neuropsychiatric disorders	Single	0.70 (0.66-0.73), p<0.001	0.72 (0.69-0.76), p<0.001	23.0
SUD	Single	0.54 (0.53-0.55), p<0.001	0.64 (0.63-0.66), p<0.001	34.2
GAD and MDD	Single	0.80 (0.79-0.81), p<0.001	0.83 (0.82-0.85), p<0.001	26.9
AGO, SOC, and OCD	Single	0.71 (0.69-0.74), p<0.001	0.83 (0.80-0.86), p<0.001	49.8
SCZ and BIP	Single	0.91 (0.88-0.93), p<0.001	0.92 (0.89-0.94), p<0.001	24.4
***Sensitivity analysis #1*: comparison with 'cleaned population'**				
Neuropsychiatric disorders	Dual	0.31 (0.22-0.45), p<0.001	0.35 (0.25-0.51), p<0.001	22.1
SUD	Dual	0.26 (0.25-0.27), p<0.001	0.38 (0.36-0.39), p<0.001	33.2
GAD and MDD	Dual	0.57 (0.53-0.60), p<0.001	0.63 (0.59-0.67), p<0.001	27.4
AGO, SOC, and OCD	Dual	0.53 (0.34-0.83), p<0.01	0.91 (0.58-1.62), p=0.669	77.7
SCZ and BIP	Dual	0.67 (0.56-0.81), p<0.001	0.78 (0.64-0.96), p=0.018	40.2
Neuropsychiatric disorders	Single	0.54 (0.51-0.56), p<0.001	0.57 (0.54-0.59), p<0.001	20.8
SUD	Single	0.50 (0.49-0.50), p<0.001	0.59 (0.58-0.60), p<0.001	32.0
GAD and MDD	Single	0.71 (0.70-0.72), p<0.001	0.75 (0.74-0.76), p<0.001	25.0
AGO, SOC, and OCD	Single	0.61 (0.59-0.63), p<0.001	0.72 (0.69-0.75), p<0.001	40.6
SCZ and BIP	Single	0.78 (0.77-0.80), p<0.001	0.81 (0.79-0.83), p<0.001	23.7
***Sensitivity analysis #2*: with repeated SCZ and BIP**				
Repeated SCZ and BIP	Dual	0.76 (0.61-0.96), p<0.01	0.87 (0.68-1.12), p=0.285	48.6
Repeated SCZ and BIP	Single	0.86 (0.84-0.89), p<0.001	0.87 (0.85-0.89), p<0.001	19.2
***Sensitivity analysis #3*: if both parents neither died before 1997 nor emigrated prior to 1997and never return to Sweden**				
Neuropsychiatric disorders	Dual	0.32 (0.22-0.47), p<0.001	0.36 (0.25-0.53), p<0.001	23.1
SUD	Dual	0.28 (0.27-0.30), p<0.001	0.40 (0.38-0.42), p<0.001	33.3
GAD and MDD	Dual	0.61 (0.58-0.65), p<0.001	0.67 (0.63-0.71), p<0.001	28.1
AGO, SOC, and OCD	Dual	0.55 (0.35-0.88), p<0.05	0.93 (0.58-1.49), p=0.765	81.0
SCZ and BIP	Dual	0.71 (0.58-0.86), p<0.01	0.80 (0.64-1.00), p=0.052	37.9
Neuropsychiatric disorders	Single	0.58 (0.55-0.60), p<0.001	0.60 (0.57-0.63), p<0.001	19.6
SUD	Single	0.51 (0.50-0.52), p<0.001	0.60 (0.59-0.61), p<0.001	32.1
GAD and MDD	Single	0.75 (0.74-0.76), p<0.001	0.78 (0.77-0.79), p<0.001	23.2
AGO, SOC, and OCD	Single	0.67 (0.64-0.70), p<0.001	0.78 (0.75-0.81), p<0.001	44.5
SCZ and BIP	Single	0.84 (0.82-0.86), p<0.001	0.85 (0.83-0.87), p<0.001	17.3

Note: Columns highlighted in orange indicate main effect moved from significant to non-significant on adjustment for parental education level

Abbreviations: ADHD, attention-deficit hyperactivity disorder; AGO, agoraphobia; ASD, autism spectrum disorder; BIP, bipolar disorder; CI, confidence intervals; GAD, generalized anxiety disorder; MDD, major depressive disorder; OCD, obsessive-compulsive disorder; OR, odds ratio; SCZ, schizophrenia; SOC, social phobia; SUD, substance use disorders; TD/CTD, Tourette's disorder and chronic tic disorders; USS, upper secondary school.

Table S6. <u>Sensitivity Analysis #4C (adjustment and mediation for parental education level):</u> Finishing University among Swedish resident offspring born in 1973-1988 (i.e., aged ≥25 by the end of follow-up)

	Parental Affected Status	Main Fully-Adjusted Model	Additional Adjustment for Highest Parent Education Level	Mediation: % Main Effect Represented by Indirect Effect
		OR (95% CI)	OR (95% CI), p-value	%
**Parents affected by any of 11 psychiatric disorders**				
Main analysis: comparison with 'uncleaned population'	Dual	0.39 (0.38-0.41), p<0.001	0.49 (0.47-0.51), p<0.001	27.2
Main analysis: comparison with 'uncleaned population'	Single	0.65 (0.64-0.66), p<0.001	0.71 (0.70-0.72), p<0.001	24.7
Main analysis: comparison with 'uncleaned population' *in offspring free from any 11 diagnoses*	Dual	0.46 (0.44-0.48), p<0.001	0.56 (0.53-0.59), p<0.001	30.9
Main analysis: comparison with 'uncleaned population' *in offspring free from any 11 diagnoses*	Single	0.69 (0.68-0.70), p<0.001	0.75 (0.74-0.76), p<0.001	27.1
Sensitivity analysis #1: comparison with 'cleaned population'	Dual	0.37 (0.35-0.38), p<0.001	0.46 (0.44-0.48), p<0.001	26.5
Sensitivity analysis #1: comparison with 'cleaned population'	Single	0.64 (0.64-0.65), p<0.001	0.70 (0.69-0.71), p<0.001	24.7
Sensitivity analysis #2: with repeated SCZ and BIP	Dual	0.39 (0.38-0.41), p<0.001	0.48 (0.46-0.51), p<0.001	27.3
Sensitivity analysis #2: with repeated SCZ and BIP	Single	0.65 (0.64-0.66), p<0.001	0.71 (0.70-0.72), p<0.001	24.8
Sensitivity analysis #3: if both parents neither died before 1997 nor emigrated prior to 1997and never return to Sweden	Dual	0.41 (0.39-0.43), p<0.001	0.49 (0.47-0.52), p<0.001	26.7
Sensitivity analysis #3: if both parents neither died before 1997 nor emigrated prior to 1997and never return to Sweden	Single	0.66 (0.65-0.67), p<0.001	0.71 (0.70-0.72), p<0.001	24.1
**Parents affected by five groups of psychiatric disorders (one group at the time)**				
***Main analysis* in the whole cohort**				
Neuropsychiatric disorders	Dual	0.32 (0.17-0.62), p<0.01	0.33 (0.17-0.67), p=0.002	15.3
SUD	Dual	0.26 (0.24-0.28), p<0.001	0.36 (0.33-0.38), p<0.001	27.3
GAD and MDD	Dual	0.64 (0.59-0.70), p<0.001	0.68 (0.62-0.74), p<0.001	22.8
AGO, SOC, and OCD	Dual	0.46 (0.23-0.90), p<0.05	0.81 (0.42-1.46), p=0.526	63.8
SCZ and BIP	Dual	0.63 (0.48-0.81), p<0.001	0.69 (0.52-0.92), p=0.010	23.3
Neuropsychiatric disorders	Single	0.59 (0.55-0.63), p<0.001	0.58 (0.54-0.61), p<0.001	10.9
SUD	Single	0.51 (0.50-0.52), p<0.001	0.59 (0.58-0.60), p<0.001	27.1
GAD and MDD	Single	0.78 (0.76-0.79), p<0.001	0.79 (0.78-0.81), p<0.001	18.0
AGO, SOC, and OCD	Single	0.68 (0.64-0.71), p<0.001	0.77 (0.73-0.81), p<0.001	38.6
SCZ and BIP	Single	0.85 (0.83-0.88), p<0.001	0.86 (0.83-0.88), p<0.001	11.1
***Main analysis* in offspring free from any 11 diagnoses**				

Neuropsychiatric disorders	Dual	0.49 (0.22-1.10), p=0.083	0.52 (0.22-1.16), p=0.110	23.1
SUD	Dual	0.32 (0.29-0.34), p<0.001	0.43 (0.40-0.47), p<0.001	30.8
GAD and MDD	Dual	0.72 (0.65-0.79), p<0.001	0.76 (0.69-0.84), p<0.001	29.3
AGO, SOC, and OCD	Dual	0.52 (0.23-1.19), p=0.123	0.93 (0.43-2.03), p=0.864	75.0
SCZ and BIP	Dual	0.66 (0.49-0.90), p<0.05	0.70 (0.50-0.98), p=0.036	17.0
Neuropsychiatric disorders	Single	0.70 (0.65-0.75), p<0.001	0.67 (0.63-0.72), p<0.001	9.3
SUD	Single	0.54 (0.53-0.55), p<0.001	0.62 (0.61-0.63), p<0.001	28.8
GAD and MDD	Single	0.82 (0.81-0.84), p<0.001	0.84 (0.83-0.86), p<0.001	21.1
AGO, SOC, and OCD	Single	0.74 (0.70-0.78), p<0.001	0.83 (0.79-0.88), p<0.001	45.2
SCZ and BIP	Single	0.92 (0.89-0.95), p<0.001	0.92 (0.89-0.95), p<0.001	12.4
***Sensitivity analysis #1*: comparison with 'cleaned population'**				
Neuropsychiatric disorders	Dual	0.29 (0.15-0.56), p<0.001	0.31 (0.16-0.61), p=0.001	15.4
SUD	Dual	0.24 (0.22-0.26), p<0.001	0.33 (0.31-0.36), p<0.001	26.5
GAD and MDD	Dual	0.59 (0.54-0.64), p<0.001	0.64 (0.58-0.69), p<0.001	22.8
AGO, SOC, and OCD	Dual	0.42 (0.21-0.81), p<0.05	0.73 (0.38-1.41), p=0.354	57.5
SCZ and BIP	Dual	0.58 (0.45-0.75), p<0.001	0.65 (0.49-0.86), p=0.002	23.3
Neuropsychiatric disorders	Single	0.54 (0.51-0.57), p<0.001	0.54 (0.51-0.57), p<0.001	12.5
SUD	Single	0.49 (0.48-0.50), p<0.001	0.58 (0.57-0.59), p<0.001	26.7
GAD and MDD	Single	0.73 (0.72-0.74), p<0.001	0.76 (0.75-0.77), p<0.001	19.9
AGO, SOC, and OCD	Single	0.62 (0.59-0.66), p<0.001	0.72 (0.68-0.75), p<0.001	35.2
SCZ and BIP	Single	0.79 (0.77-0.82), p<0.001	0.81 (0.78-0.83), p<0.001	16.2
***Sensitivity analysis #2*: with repeated SCZ and BIP**				
Repeated SCZ and BIP	Dual	0.62 (0.45-0.86), p<0.01	0.69 (0.49-0.97), p=0.035	20.8
Repeated SCZ and BIP	Single	0.87 (0.84-0.90), p<0.001	0.87 (0.84-0.90), p<0.001	10.3
***Sensitivity analysis #3*: if both parents neither died before 1997 nor emigrated prior to 1997and never return to Sweden**				
Neuropsychiatric disorders	Dual	0.33 (0.17-0.64), p<0.01	0.35 (0.17-0.69), p=0.003	16.6
SUD	Dual	0.27 (0.25-0.29), p<0.001	0.37 (0.34-0.39), p<0.001	27.0
GAD and MDD	Dual	0.64 (0.59-0.70), p<0.001	0.68 (0.63-0.75), p<0.001	24.1
AGO, SOC, and OCD	Dual	0.49 (0.25-0.97), p<0.05	0.85 (0.44-1.65), p=0.631	67.2
SCZ and BIP	Dual	0.55 (0.41-0.73), p<0.001	0.60 (0.44-0.82), p=0.001	18.7
Neuropsychiatric disorders	Single	0.59 (0.55-0.63), p<0.001	0.58 (0.54-0.62), p<0.001	11.2
SUD	Single	0.51 (0.50-0.52), p<0.001	0.59 (0.58-0.60), p<0.001	26.7
GAD and MDD	Single	0.77 (0.76-0.78), p<0.001	0.79 (0.78-0.80), p<0.001	18.0
AGO, SOC, and OCD	Single	0.68 (0.65-0.72), p<0.001	0.77 (0.73-0.81), p<0.001	38.4
SCZ and BIP	Single	0.85 (0.83-0.88), p<0.001	0.85 (0.82-0.88), p<0.001	8.7

Note: Columns highlighted in orange indicate main effect moved from significant to non-significant on adjustment for parental education level

Abbreviations: ADHD, attention-deficit hyperactivity disorder; AGO, agoraphobia; ASD, autism spectrum disorder; BIP, bipolar disorder; CI, confidence intervals; GAD, generalized anxiety disorder; MDD, major depressive disorder; OCD, obsessive-compulsive disorder; OR, odds ratio; SCZ, schizophrenia; SOC, social phobia; SUD, substance use disorders; TD/CTD, Tourette's disorder and chronic tic disorders; USS, upper secondary school.

Table S7. Sensitivity analysis #1 (comparison with cleaned population): educational outcomes in offspring of parents dual- and single-affected by five groups of psychiatric disorders (one group at the time). OR (95% CI) for achieving compulsory and post compulsory educational outcomes, compared to individuals with parents from the general population (*cleaned population*[a]).

	Dual-affected parental pairs				Single-affected parental pairs			
	**Offspring of affected parents**	**Offspring of non-affected parents**	**Crude model**	**Fully-adjusted** [b]	**Offspring of affected parents**	**Offspring of non-affected parents**	**Crude model**	**Fully-adjusted**[b]
	**n (%)**	**n (%)**	**OR (95% CI)**	**OR (95% CI)**	**n (%)**	**n (%)**	**OR (95% CI)**	**OR (95% CI)**
**ELIGIBILITY TO ACCESS USS**[c]								
**Neuropsychiatric disorders**[d]	**n=607**	**n=1,169,427**			**n=21,727**	**n=1,169,427**		
achieved	439 (72.32)	1,084,368 (92.73)	0.20 (0.17-0.25)*	0.25 (0.20-0.30)*	17,086 (78.64)	1,084,368 (92.73)	0.29 (0.28-0.30)*	0.33 (0.31-0.34)*
**Substance use disorders**	**n=10,518**	**n=1,169,427**			**n=121,273**	**n=1,169,427**		
achieved	7,523 (71.53)	1,084,368 (92.73)	0.20 (0.19-0.21)*	0.20 (0.19-0.21)*	99,839 (82.33)	1,084,368 (92.73)	0.36 (0.36-0.37)*	0.38 (0.37-0.38)*
**GAD and MDD**	**n=6,291**	**n=1,169,427**			**n=135,587**	**n=1,169,427**		
achieved	4,973 (79.05)	1,084,368 (92.73)	0.30 (0.28-0.32)*	0.32 (0.30-0.34)*	116,416 (85.86)	1,084,368 (92.73)	0.48 (0.47-0.48)*	0.49 (0.48-0.50)*
**AGO, SOC, and OCD**	**n=189**	**n=1,169,427**			**n=19,446**	**n=1,169,427**		
achieved	128 (67.72)	1,084,368 (92.73)	0.16 (0.12-0.23)*	0.20 (0.14-0.28)*	16,029 (82.43)	1,084,368 (92.73)	0.37 (0.35-0.38)*	0.40 (0.38-0.42)*
**SCZ and BIP**	**n=475**	**n=1,169,427**			**n=32,416**	**n=1,169,427**		
achieved	384 (80.84)	1,084,368 (92.73)	0.33 (0.26-0.42)*	0.33 (0.26-0.43)*	27,978 (86.31)	1,084,368 (92.73)	0.49 (0.48-0.51)*	0.51 (0.49-0.53)*
**FINISHING USS**[e]								
**Neuropsychiatric disorders**[d]	**n=498**	**n=1,627,976**			**n=21,146**	**n=1,627,976**		
achieved	185 (37.15)	1,352,772 (83.10)	0.12 (0.10-0.15)*	0.16 (0.13-0.20)*	11,828 (55.93)	1,352,772 (83.10)	0.26 (0.25-0.27)*	0.31 (0.30-0.32)*
**Substance use disorders**	**n=16,344**	**n=1,627,976**			**n=188,577**	**n=1,627,976**		
achieved	8,171 (49.99)	1,352,772 (83.10)	0.20 (0.19-0.21)*	0.20 (0.19-0.21)*	124,865 (66.21)	1,352,772 (83.10)	0.40 (0.39-0.40)*	0.40 (0.39-0.40)*
**GAD and MDD**	**n=7,723**	**n=1,627,976**			**n=181,376**	**n=1,627,976**		
achieved	4,713 (61.03)	1,352,772 (83.10)	0.32 (0.30-0.33)*	0.34 (0.33-0.36)*	129,082 (71.17)	1,352,772 (83.10)	0.50 (0.49-0.51)*	0.52 (0.51-0.53)*
**AGO, SOC, and OCD**	**n=194**	**n=1,627,976**			**n=22,334**	**n=1,627,976**		
achieved	92 (47.42)	1,352,772 (83.10)	0.18 (0.13-0.25)*	0.24 (0.17-0.33)*	14,487 (64.87)	1,352,772 (83.10)	0.37 (0.36-0.39)*	0.42 (0.41-0.43)*
**SCZ and BIP**	**n=674**	**n=1,627,976**			**n=46,253**	**n=1,627,976**		
achieved	417 (61.87)	1,352,772 (83.10)	0.33 (0.28-0.39)*	0.34 (0.29-0.40)*	32,766 (70.84)	1,352,772 (83.10)	0.49 (0.48-0.50)*	0.50 (0.49-0.51)*
**STARTING UNIVERSITY**[f]								
**Neuropsychiatric disorders**[d]	**n=370**	**n=1,458,407**			**n=17,235**	**n=1,458,407**		
achieved	42 (11.35)	590,047 (40.46)	0.19 (0.13-0.26)*	0.31 (0.22-0.45)*	3,747 (21.74)	590,047 (40.46)	0.41 (0.39-0.43)*	0.54 (0.51-0.56)*
**Substance use disorders**	**n=14,998**	**n=1,458,407**			**n=172,571**	**n=1,458,407**		
achieved	2,254 (15.03)	590,047 (40.46)	0.26 (0.25-0.27)*	0.26 (0.25-0.27)*	43,800 (25.38)	590,047 (40.46)	0.50 (0.49-0.51)*	0.50 (0.49-0.50)*
**GAD and MDD**	**n=6,720**	**n=1,458,407**			**n=161,369**	**n=1,458,407**		
achieved	1,763 (26.24)	590,047 (40.46)	0.52 (0.49-0.56)*	0.57 (0.53-0.60)*	51,234 (31.75)	590,047 (40.46)	0.68 (0.67-0.69)*	0.71 (0.70-0.72)*
**AGO, SOC, and OCD**	**n=161**	**n=1,458,407**			**n=19,305**	**n=1,458,407**		
achieved	32 (19.88)	590,047 (40.46)	0.36 (0.24-0.56)*	0.53 (0.34-0.83)§	5,077 (26.30)	590,047 (40.46)	0.52 (0.51-0.54)*	0.61 (0.59-0.63)*
**SCZ and BIP**	**n=604**	**n=1,458,407**			**n=41,657**	**n=1,458,407**		
achieved	188 (31.13)	590,047 (40.46)	0.66 (0.55-0.80)*	0.67 (0.56-0.81)*	14,353 (34.46)	590,047 (40.46)	0.77 (0.76-0.79)*	0.78 (0.77-0.80)*
**FINISHING UNIVERSITY**[g]								

**Neuropsychiatric disorders[d]**	**n=165**	**n=1,099,309**			**n=10,103**	**n=1,099,309**		
achieved	10 (6.06)	303,014 (27.56)	0.17 (0.09-0.32)*	0.29 (0.15-0.56)*	1,353 (13.39)	303,014 (27.56)	0.41 (0.38-0.43)*	0.54 (0.51-0.57)*
**Substance use disorders**	**n=11,704**	**n=1,099,309**			**n=134,529**	**n=1,099,309**		
achieved	969 (8.28)	303,014 (27.56)	0.24 (0.22-0.25)*	0.24 (0.22-0.26)*	21,324 (15.85)	303,014 (27.56)	0.49 (0.48-0.50)*	0.49 (0.48-0.50)*
**GAD and MDD**	**n=4,709**	**n=1,099,309**			**n=118,284**	**n=1,099,309**		
achieved	808 (17.16)	303,014 (27.56)	0.54 (0.50-0.59)*	0.59 (0.54-0.64)*	24,927 (21.07)	303,014 (27.56)	0.70 (0.69-0.71)*	0.73 (0.72-0.74)*
**AGO, SOC, and OCD**	**n=98**	**n=1,099,309**			**n=12,992**	**n=1,099,309**		
achieved	10 (10.20)	303,014 (27.56)	0.30 (0.16-0.56)*	0.42 (0.21-0.81)#	2,216 (17.06)	303,014 (27.56)	0.54 (0.51-0.57)*	0.62 (0.59-0.66)*
**SCZ and BIP**	**n=440**	**n=1,099,309**			**n=31,360**	**n=1,099,309**		
achieved	77 (17.50)	303,014 (27.56)	0.56 (0.43-0.72)*	0.58 (0.45-0.75)*	7,177 (22.89)	303,014 (27.56)	0.78 (0.76-0.80)*	0.79 (0.77-0.82)*

Note: All models are clustered by family identification number with robust standard error estimation (sandwich estimator). Within each group, *'dual-affected parental pairs'* imply that both parents have records of the disorders from the group in question (both parents diagnosed with either the same or different disorders within the group; for example, attention-deficit hyperactivity disorder, or autism spectrum disorder, or Tourette's disorder and chronic tic disorders in the analysis of neuropsychiatric disorders). Within each group, *'single-affected parental pairs'* imply that one parent has at least one disorder from the group in question and the other parent is free from any disorders from the corresponding group. For each educational outcome, separate sub-cohorts of index offspring were created, comprising the individuals who had the time necessary to achieve the corresponding outcome.

[a] 'Cleaned population' implies that the reference group is composed of the offspring with both parents being free from any 11 psychiatric disorders.
[b] Adjusted for offspring year of birth (5-year categories starting from 1973), offspring sex, and maternal and paternal year of birth in decades (<1940, 1950s, 1960s, 1970s, ≥1970)
[c] Offspring who graduated from compulsory school in 1998-2013. The graduation years were chosen due to the different eligibility criteria used prior to 1998.
[d] Neuropsychiatric disorder group includes attention-deficit hyperactivity disorder, autism spectrum disorder, and Tourette's disorder and chronic tic disorders.
[e] Offspring born in 1973-1994 (i.e., aged ≥19 by the end of follow-up) and who was alive and living in Sweden at age 19 years.
[f] Offspring born in 1973-1992 (i.e., aged ≥21 by the end of follow-up) and who was alive and living in Sweden at age 21 years.
[g] Offspring born in 1973-1988 (i.e., aged ≥25 by the end of follow-up) and who was alive and living in Sweden at age 25 years.

# p-value <0.05, § p-value <0.01, * p-value <0.001

Abbreviation: ADHD, attention-deficit hyperactivity disorder; AGO, agoraphobia; ASD, autism spectrum disorder; BIP, bipolar disorder; CI, confidence intervals; GAD, generalized anxiety disorder; MDD, major depressive disorder; OCD, obsessive-compulsive disorder; OR, odds ratio; SCZ, schizophrenia; SOC, social phobia; SUD, substance use disorders; TD/CTD, Tourette's disorder and chronic tic disorders; USS, upper secondary school.

Table S8. <u>Sensitivity analysis #1 (comparison with cleaned population)</u>: educational outcomes in offspring of parents dual- and single-affected by any of 11 psychiatric disorders. OR (95% CI) for achieving compulsory and post compulsory educational outcomes, compared to individuals with parents from the general population (*cleaned population*[a]).

	**Dual-affected parental pairs**				**Single-affected parental pairs**			
	**Offspring of affected pairs**	**Offspring of unaffected pairs**	**Crude model**	**Fully-adjusted[b]**	**Offspring of affected pairs**	**Offspring of unaffected pairs**	**Crude model**	**Fully-adjusted[b]**
	**n (%)**	**n (%)**	**OR (95% CI)**	**OR (95% CI)**	**n (%)**	**n (%)**	**OR (95% CI)**	**OR (95% CI)**
**Eligibility to access USS[c]**	**n=24,939**	**n=1,169,427**			**n=222,501**	**n=1,169,427**		
achieved	18,777 (75.29)	1,084,368 (92.73)	0.24 (0.23-0.25)*	0.25 (0.24-0.26)*	190,240 (85.50)	1,084,368 (92.73)	0.46 (0.45-0.47)*	0.48 (0.47-0.48)*
**Finishing USS[d]**	**n=35,486**	**n=1,627,976**			**n=322,057**	**n=1,627,976**		
achieved	19,628 (55.31)	1,352,772 (83.10)	0.25 (0.24-0.26)*	0.26 (0.25-0.27)*	227,510 (70.64)	1,352,772 (83.10)	0.49 (0.48-0.49)*	0.49 (0.49-0.50)*
**Staring university[e]**	**n=31,982**	**n=1,458,407**			**n=290,756**	**n=1,458,407**		
achieved	6,381 (19.95)	590,047 (40.46)	0.37 (0.36-0.38)*	0.38 (0.36-0.39)*	87,862 (30.22)	590,047 (40.46)	0.64 (0.63-0.64)*	0.64 (0.64-0.65)*
**Finishing university[f]**	**n=24,096**	**n=1,099,309**			**n=220,787**	**1,099,309**		
achieved	2,871 (11.91)	303,014 (27.56)	0.35 (0.34-0.37)*	0.37 (0.35-0.38)*	43,118 (19.53)	303,014 (27.56)	0.64 (0.63-0.65)*	0.64 (0.64-0.65)*

ote: All models are clustered by family identification number with robust standard error estimation (sandwich estimator). '*Dual-affected pairs*' imply that both parents have a record of at east one of 11 disorders (being diagnosed with either the same disorder or with different ones), and '*single-affected pairs*' imply that one parent has a record of any such disorders, the other s free from any 11 disorders. For each educational outcome, separate sub-cohorts of index offspring were created, comprising the individuals who had the time necessary to achieve the orresponding outcome.

'Cleaned population' implies that the reference group is composed of the offspring with both parents being free from any 11 psychiatric disorders.
Adjusted for offspring year of birth (5-year categories starting from 1973), offspring sex, and maternal and paternal year of birth in decades (<1940, 1950s, 1960s, 1970s, ≥1970)
Offspring who graduated from compulsory school in 1998-2013. The graduation years were chosen due to the different eligibility criteria used prior to 1998.
Offspring born in 1973-1994 (i.e., aged ≥19 by the end of follow-up) and who was alive and living in Sweden at age 19 years.
Offspring born in 1973-1992 (i.e., aged ≥21 by the end of follow-up) and who was alive and living in Sweden at age 21 years.
Offspring born in 1973-1988 (i.e., aged ≥25 by the end of follow-up) and who was alive and living in Sweden at age 25 years.

p-value <0.001

bbreviation: CI, confidence intervals; OR, odds ratio; USS, upper secondary school.

Table S9. Sensitivity analysis #1 (comparison with cleaned population): educational outcomes in offspring of parents single-affected by each specific disorder (one disorder at the time). OR (95% CI) for achieving compulsory and post compulsory educational outcomes, compared to individuals with parents from the general population (*cleaned population*[a]).

Parental diagnoses	Offspring of affected pairs	Offspring of unaffected pairs	Fully-adjusted[b]
	n (%)	n (%)	OR (95% CI)
**Eligibility to access USS[c]**			
ADHD	15,824 (78.30)	1,084,368 (92.73)	0.32 (0.31-0.33)*
ASD	2,139 (80.14)	1,084,368 (92.73)	0.35 (0.32-0.39)*
SCZ	5,133 (83.23)	1,084,368 (92.73)	0.40 (0.37-0.43)*
BIP	24,394 (86.82)	1,084,368 (92.73)	0.53 (0.51-0.55)*
MDD	111,446 (85.90)	1,084,368 (92.73)	0.50 (0.49-0.51)*
GAD	14,250 (83.63)	1,084,368 (92.73)	0.42 (0.40-0.44)*
AGO	5,129 (80.02)	1,084,368 (92.73)	0.35 (0.32-0.37)*
SOC	7,408 (80.50)	1,084,368 (92.73)	0.35 (0.33-0.38)*
OCD	5,728 (85.07)	1,084,368 (92.73)	0.48 (0.45-0.52)*
SUD	99,839 (82.33)	1,084,368 (92.73)	0.38 (0.37-0.38)*
TD/CTD	450 (82.27)	1,084,368 (92.73)	0.40 (0.32-0.52)*
**Finishing USS[d]**			
ADHD	10,784 (55.29)	1,352,772 (83.10)	0.30 (0.29-0.31)*
ASD	1,513 (58.08)	1,352,772 (83.10)	0.34 (0.31-0.37)*
SCZ	7,135 (67.78)	1,352,772 (83.10)	0.42 (0.40-0.44)*
BIP	27,947 (71.56)	1,352,772 (83.10)	0.52 (0.51-0.53)*
MDD	123,853 (71.21)	1,352,772 (83.10)	0.52 (0.51-0.53)*
GAD	14,475 (67.45)	1,352,772 (83.10)	0.45 (0.44-0.46)*
AGO	4,406 (61.02)	1,352,772 (83.10)	0.36 (0.35-0.38)*
SOC	6,305 (61.73)	1,352,772 (83.10)	0.37 (0.36-0.39)*
OCD	5,495 (69.56)	1,352,772 (83.10)	0.50 (0.48-0.53)*
SUD	124,865 (66.21)	1,352,772 (83.10)	0.40 (0.39-0.40)*
TD/CTD	393 (64.85)	1,352,772 (83.10)	0.41 (0.34-0.50)*
**Staring university[e]**			
ADHD	3,339 (21.08)	590,047 (40.46)	0.52 (0.50-0.54)*
ASD	507 (23.83)	590,047 (40.46)	0.60 (0.53-0.67)*
SCZ	3,063 (31.33)	590,047 (40.46)	0.66 (0.63-0.69)*
BIP	12,323 (35.22)	590,047 (40.46)	0.81 (0.79-0.84)*
MDD	49,323 (31.87)	590,047 (40.46)	0.71 (0.70-0.72)*
GAD	5,395 (28.54)	590,047 (40.46)	0.64 (0.62-0.67)*

AGO	1,380 (22.18)	590,047 (40.46)	0.50 (0.47-0.54)*
SOC	2,037 (23.36)	590,047 (40.46)	0.54 (0.51-0.57)*
OCD	2,160 (31.52)	590,047 (40.46)	0.75 (0.71-0.80)*
SUD	43,800 (25.38)	590,047 (40.46)	0.50 (0.49-0.50)*
TD/CTD	157 (29.96)	590,047 (40.46)	0.70 (0.56-0.87)*
**Finishing university[f]**			
ADHD	1,199 (13.06)	303,014 (27.56)	0.53 (0.50-0.57)*
ASD	172 (13.36)	303,014 (27.56)	0.54 (0.45-0.64)*
SCZ	1,489 (19.07)	303,014 (27.56)	0.62 (0.58-0.66)*
BIP	6,205 (23.79)	303,014 (27.56)	0.84 (0.81-0.86)*
MDD	24,066 (21.21)	303,014 (27.56)	0.74 (0.72-0.75)*
GAD	2,482 (18.36)	303,014 (27.56)	0.65 (0.62-0.68)*
AGO	557 (13.57)	303,014 (27.56)	0.50 (0.45-0.55)*
SOC	843 (14.71)	303,014 (27.56)	0.55 (0.51-0.60)*
OCD	1,007 (21.35)	303,014 (27.56)	0.77 (0.72-0.84)*
SUD	21,324 (15.85)	303.014 (27.56)	0.49 (0.49-0.50)*
TD/CTD	62 (17.61)	303,014 (27.56)	0.62 (0.46-0.83)*

Note: All models are clustered by family identification number with robust standard error estimation (sandwich estimator). For each disorder, *'single-affected parental pairs'* imply that one parent (either mother or father) has a disorder in question and the other parent is free from such disorder. For each educational outcome, separate subcohorts of index offspring were created, comprising the individuals who had the time necessary to achieve the corresponding outcome. Within each sub-cohort, a total number of offspring of affected pairs varies between the disorders, while total number of offspring of unaffected pairs is the same, that is: for eligibility to access USS - 1,169,427 individuals, for finishing USS – 1,627,976 individuals, for starting university – 1,458,407 individuals, for finishing university – 1,099,309 individuals. Each disorder is analyzed as a separate entity, therefore, offspring of parents affected by disorder(s) other than the disorder in question are excluded from the corresponding analysis.

[a] 'Cleaned population' implies that the reference group is composed of the offspring with both parents being free from any 11 psychiatric disorders.
[b] Adjusted for offspring year of birth (5-year categories starting from 1973), offspring sex, and maternal and paternal year of birth in decades (<1940, 1950s, 1960s, 1970s, ≥1970)
[c] Offspring who graduated from compulsory school in 1998-2013. The graduation years were chosen due to the different eligibility criteria used prior to 1998.
[d] Offspring born in 1973-1994 (i.e., aged ≥19 by the end of follow-up) and who was alive and living in Sweden at age 19.
[e] Offspring born in 1973-1992 (i.e., aged ≥21 by the end of follow-up) and who was alive and living in Sweden at age 21.
[f] Offspring born in 1973-1988 (i.e., aged ≥25 by the end of follow-up) and who was alive and living in Sweden at age 25.

* p-value <0.001

Abbreviation: ADHD, attention-deficit hyperactivity disorder; AGO, agoraphobia; ASD, autism spectrum disorder; BIP, bipolar disorder; CI, confidence intervals; GAD, generalized anxiety disorder; MDD, major depressive disorder; OCD, obsessive-compulsive disorder; OR, odds ratio; SCZ, schizophrenia; SOC, social phobia; SUD, substance use disorders; TD/CTD, Tourette's disorder and chronic tic disorders; USS, upper secondary school.

Table S10. Sensitivity analysis #2 (with repeated schizophrenia and bipolar disorders): educational outcomes in offspring of parents dual- and single-affected by any 11 psychiatric disorders. OR (95% CI) for achieving compulsory and post compulsory educational outcomes, compared to individuals with parents from the general population (*uncleaned population*[a]).

	Dual-affected parental pairs				Single-affected parental pairs			
	**Offspring of affected pairs**	**Offspring of unaffected pairs**	**Crude model**	**Fully-adjusted**[b]	**Offspring of affected pairs**	**Offspring of unaffected pairs**	**Crude model**	**Fully-adjusted**[b]
	**n (%)**	**n (%)**	**OR (95% CI)**	**OR (95% CI)**	**n (%)**	**n (%)**	**OR (95% CI)**	**OR (95% CI)**
**Eligibility to access USS**[c]	**n=24,544**	**n=1,392,323**			**n=220,283**	**n=1,196,184**		
achieved	18,446 (75.15)	1,274,939 (91.57)	0.28 (0.27-0.29)*	0.29 (0.28-0.30)*	188,586 (85.46)	1,104,799 (92.36)	0.49 (0.48-0.49)*	0.50 (0.49-0.51)*
**Finishing USS**[d]	**n=34,888**	**n=1,950,631**			**n=319,058**	**n=1,666,461**		
achieved	19,236 (55.14)	1,580,674 (81.03)	0.29 (0.28-0.29)*	0.30 (0.29-0.30)*	225,142 (70.56)	1,374,768 (82.50)	0.51 (0.50-0.51)*	0.51 (0.51-0.52)*
**Staring university**[e]	**n=31,425**	**n=1,749,720**			**n=288,019**	**n=1,493,126**		
achieved	6,221 (19.80)	678,069 (38.75)	0.39 (0.38-0.40)*	0.40 (0.39-0.41)*	86,697 (30.10)	597,593 (40.02)	0.64 (0.64-0.65)*	0.65 (0.64-0.66)*
**Finishing university**[f]	**n=23,680**	**n=1,320,512**			**n=218,565**	**n=1,125,627**		
achieved	2,802 (11.83)	346,201 (26.22)	0.38 (0.36-0.39)*	0.39 (0.38-0.41)*	42,515 (19.45)	306,488 (27.23)	0.64 (0.64-0.65)*	0.65 (0.64-0.66)*

ote: All models are clustered by family identification number with robust standard error estimation (sandwich estimator). '*Dual-affected parental pairs*' imply that both parents have a record f at least one of 11 disorders (being diagnosed with either the same disorder or with different ones), and '*single-affected parental pairs*' imply that one parent has a record of any such disorders, he other is free from any 11 disorders. For each educational outcome, separate sub-cohorts of index offspring were created, comprising the individuals who had the time necessary to achieve he corresponding outcome.

'Uncleaned population' imposes no restriction on parental diagnoses in the comparison group and represents, in each relevant analysis, the rest of study population. This approach reflects the igh rates of psychiatric comorbidity and ensures obtaining real-world, conservative measures of associations.
Adjusted for offspring year of birth (5-year categories starting from 1973), offspring sex, and maternal and paternal year of birth in decades (<1940, 1950s, 1960s, 1970s, ≥1970)
Offspring who graduated from compulsory school in 1998-2013 (n=1,416,867). The graduation years were chosen due to the different eligibility criteria used prior to 1998.
Offspring born in 1973-1994 (i.e., aged ≥19 by the end of follow-up) and who was alive and living in Sweden at age 19 years (n=1,985,519).
Offspring born in 1973-1992 (i.e., aged ≥21 by the end of follow-up) and who was alive and living in Sweden at age 21 years (n=1,781,145).
Offspring born in 1973-1988 (i.e., aged ≥25 by the end of follow-up) and who was alive and living in Sweden at age 25 years (n=1,344,192).

p-value <0.001

bbreviation: CI, confidence intervals; OR, odds ratio; USS, upper secondary school.

Table S11. <u>Sensitivity analysis #2 (with repeated schizophrenia and bipolar disorders):</u> educational outcomes in offspring of parents dual- or single affected by the 'SCZ-R and/or BIP-R' group. OR (95% CI) for achieving compulsory and post compulsory educational outcomes, compared to individuals with parents from the general population (*uncleaned population*[a]).

	Dual-affected parental pairs				Single-affected parental pairs			
	**Offspring of affected pairs**	**Offspring of unaffected pairs**	**Crude model**	**Fully-adjusted[b]**	**Offspring of affected pairs**	**Offspring of unaffected pairs**	**Crude model**	**Fully-adjusted[b]**
	**n (%)**	**n (%)**	**OR (95% CI)**	**OR (95% CI)**	**n (%)**	**n (%)**	**OR (95% CI)**	**OR (95% CI)**
**Eligibility to access USS[c]**	**n=331**	**n=1,416,556**			**n=25,012**	**n=1,391,855**		
achieved	261 (83.92)	1,293,124 (91.29)	0.50 (0.37-0.68)*	0.49 (0.36-0.66)*	21,609 (86.39)	1,271,776 (91.37)	0.60 (0.58-0.62)*	0.62 (0.59-0.64)*
**Finishing USS[d]**	**n=441**	**n=1,985,078**			**n=35,395**	**n=1,950,124**		
achieved	272 (61.68)	1,599,638 (80.58)	0.39 (0.32-0.47)*	0.39 (0.32-0.48)*	25,186 (71.16)	1,574,724 (80.75)	0.59 (0.57-0.60)*	0.60 (0.58-0.61)*
**Starting university[e]**	**n=389**	**n=1,780,756**			**n=31,824**	**n=1,749,321**		
achieved	126 (32.39)	684,164 (38.42)	0.77 (0.62-0.96)#	0.76 (0.61-0.96)#	11,016 (34.62)	673,274 (38.49)	0.85 (0.82-0.87)*	0.86 (0.84-0.89)*
**Finishing university[f]**	**n=286**	**n=1,343,906**			**n=23,837**	**n=1,320,355**		
achieved	50 (17.48)	348,953 (25.97)	0.60 (0.44-0.82)§	0.62 (0.45-0.86)§	5,515 (23.14)	343,488 (26.01)	0.86 (0.83-0.88)*	0.87 (0.84-0.90)*

ote: All models are clustered by family identification number with robust standard error estimation (sandwich estimator). *'Dual-affected parental pairs'* imply that both parents have repeated ecords of either schizophrenia (SCZ-R) or bipolar disorder (BIP-R) (e.g., SCZ-R/SCZ-r or BIP-R/BIP-R or SCZ-R/BIP-R). *'Single-affected parental pairs'* imply that one parent has either CZ-R or BIP-R and the other parent is free from any of such disorders. For each educational outcome, separate sub-cohorts of index offspring were created, comprising the individuals who ad the time necessary to achieve the corresponding outcome.

'Uncleaned population' imposes no restriction on parental diagnoses in the comparison group and represents, in each relevant analysis, the rest of study population. This approach reflects he high rates of psychiatric comorbidity and ensures obtaining real-world, conservative measures of associations.
Adjusted for offspring year of birth (5-year categories starting from 1973), offspring sex, and maternal and paternal year of birth in decades (<1940, 1950s, 1960s, 1970s, ≥1970)
Offspring who graduated from compulsory school in 1998-2013 (n=1,416,867). The graduation years were chosen due to the different eligibility criteria used prior to 1998.
Offspring born in 1973-1994 (i.e., aged ≥19 by the end of follow-up) and who was alive and living in Sweden at age 19 years (n=1,985,519).
Offspring born in 1973-1992 (i.e., aged ≥21 by the end of follow-up) and who was alive and living in Sweden at age 21 years (n=1,781,145).
Offspring born in 1973-1988 (i.e., aged ≥25 by the end of follow-up) and who was alive and living in Sweden at age 25 years (n=1,344,192).

p-value <0.05; § p-value <0.01; * p-value <0.001

bbreviation: BIP-R, repeated diagnoses of bipolar disorder; CI, confidence intervals; OR, odds ration; SCZ-R, repeated diagnoses of schizophrenia; USS, upper secondary school.

Table S12. Sensitivity analysis #2 (with repeated schizophrenia and bipolar disorders): educational outcomes in offspring with parents single-affected by SCZ-r or BIP-r (one disorder at the time). OR (95% CI) for achieving compulsory and post compulsory educational outcomes, compared to individuals with parents from the general population (*uncleaned population*[a]).

	Offspring of affected pairs	Offspring of unaffected pairs	Fully-adjusted[b]
	n (%)	n (%)	OR (95% CI)
**ELIGIBILITY TO ACCESS USS**[c]			
SCZ_r	3,516 (82.90)	1,289,869 (91.31)	0.47 (0.43-0.51)*
BIP_r	18,839 (86.98)	1,274,546 (91.35)	0.65 (0.62-0.68)*
**FINISHING USS**[d]			
SCZ_r	4,939 (67.17)	1.594,971 (80.63)	0.48 (0.46-0.51)*
BIP_r	21,454 (72.07)	1,578,456 (80.71)	0.63 (0.61-0.65)*
**STARING UNIVERSITY**[e]			
SCZ_r	2,123 (31.04)	682,167 (38.45)	0.71 (0.67-0.75)*
BIP_r	9,447 (35.52)	674,843 (38.46)	0.90 (0.87-0.93)*
**FINISHING UNIVERSITY**[f]			
SCZ_r	1,024 (18.70)	347,979 (25.99)	0.66 (0.61-0.71)*
BIP_r	4,763 (24.20)	344,240 (25.99)	0.93 (0.90-0.96)*

Note: All models are clustered by family identification number with robust standard error estimation (sandwich estimator). For each disorder, *'single-affected parental pairs'* imply that one parent (either mother or father) has a disorder in question and the other parent is free from such disorder. For each educational outcome, separate sub-cohorts of index offspring were created, comprising the individuals who had the time necessary to achieve the corresponding outcome.

[a] 'Uncleaned population' imposes no restriction on parental diagnoses in the comparison group and represents, in each relevant analysis, the rest of study population. This approach reflects the high rates of psychiatric comorbidity and ensures obtaining real-world, conservative measures of associations.
[b] Adjusted for offspring year of birth (5-year categories starting from 1973), offspring sex, and maternal and paternal year of birth in decades (<1940, 1950s, 1960s, 1970s, ≥1970)
[c] Individuals who graduated from compulsory school in 1998-2013 (n=1,416,867). The graduation years were chosen due to the different eligibility criteria used prior to 1998.
[d] Offspring born in 1973-1994 (i.e., aged ≥19 by the end of follow-up) and who was alive and living in Sweden at age 19 years (n=1,985,519).
[e] Offspring born in 1973-1992 (i.e., aged ≥21 by the end of follow-up) and who was alive and living in Sweden at age 21 years (n=1,781,145).
[f] Offspring born in 1973-1988 (i.e., aged ≥25 by the end of follow-up) and who was alive and living in Sweden at age 25 years (n=1,344,192).

* p-value <0.001

Abbreviation: BIP_r, repeated diagnoses of bipolar disorder; CI, confidence intervals; OR, odds ration; SCZ_r, repeated diagnoses of schizophrenia; USS, upper secondary school.

[able S13. <u>Sensitivity Analysis #3 (if both parents neither died before 1997 nor emigrated prior to 1997and never return to Sweden)</u>: educational outcomes in offspring with parents dual- and single-affected by any of 11 psychiatric disorders. OR (95% CI) for achieving compulsory and post compulsory educational outcomes, compared to individuals with parents from the general population (*uncleaned population*[a]).

	Dual-affected parental pairs				Single-affected parental pairs			
	**Offspring of affected pairs**	**Offspring of unaffected pairs**	**Crude model**	**Fully-adjusted[b]**	**Offspring of affected pairs**	**Offspring of unaffected pairs**	**Crude model**	**Fully-adjusted[b]**
	**n (%)**	**n (%)**	**OR (95% CI)**	**OR (95% CI)**	**n (%)**	**n (%)**	**OR (95% CI)**	**OR (95% CI)**
**Eligibility to access USS[c]**	**n=22,909**	**n=1,331,530**			**n=209,757**	**n=1,144,682**		
achieved	17,300 (75.52)	1,220,229 (91.64)	0.28 (0.27-0.29)*	0.30 (0.29-0.31)*	179,539 (85.59)	1,058,007 (92.43)	0.49 (0.48-0.49)*	0.50 (0.49-0.51)*
**Finishing USS[d]**	**n=31,590**	**n=1,845,030**			**n=298,405**	**n=1,578,215**		
achieved	17,579 (55.65)	1,500,807 (81.34)	0.29 (0.28-0.30)*	0.30 (0.29-0.31)*	211,777 (70.97)	1,306,609 (82.79)	0.51 (0.50-0.51)*	0.51 (0.51-0.52)*
**Staring university[e]**	**n=28,323**	**n=1,652,881**			**n=268,890**	**n=1,412,314**		
achieved	5,734 (20.25)	642,458 (38.87)	0.40 (0.39-0.41)*	0.41 (0.40-0.42)*	81,616 (30.35)	566,576 (40.12)	0.65 (0.64-0.66)*	0.66 (0.65-0.66)*
**Finishing university[f]**	**n=21,042**	**n=1,242,854**			**n=202,951**	**n=1,060,945**		
achieved	2,583 (12.28)	328,098 (26.40)	0.39 (0.37-0.41)*	0.41 (0.39-0.43)*	40,004 (19.71)	290,677 (27.40)	0.65 (0.64-0.66)*	0.66 (0.65-0.67)*

ote: All models are clustered by family identification number with robust standard error estimation (sandwich estimator). '*Dual-affected parental pairs*' imply that both parents have a record f at least one of 11 disorders (being diagnosed with either the same disorder or with different ones), and '*single-affected parental pairs*' imply that one parent has a record of any such disorders, he other is free from any 11 disorders. For each educational outcome, separate sub-cohorts of index offspring were created, comprising the individuals who had the time necessary to achieve he corresponding outcome.

'Uncleaned population' imposes no restriction on parental diagnoses in the comparison group and represents, in each relevant analysis, the rest of study population. This approach reflects the igh rates of psychiatric comorbidity and ensures obtaining real-world, conservative measures of associations.
Adjusted for offspring year of birth (5-year categories starting from 1973), offspring sex, and maternal and paternal year of birth in decades (<1940, 1950s, 1960s, 1970s, ≥1970)
Offspring who graduated from compulsory school in 1998-2013 (n=1,354,439). The graduation years were chosen due to the different eligibility criteria used prior to 1998.
Offspring born in 1973-1994 (i.e., aged ≥19 by the end of follow-up) and who was alive and living in Sweden at age 19 years (n=1,876,620).
Offspring born in 1973-1992 (i.e., aged ≥21 by the end of follow-up) and who was alive and living in Sweden at age 21 years (n=1,681,204).
Offspring born in 1973-1988 (i.e., aged ≥25 by the end of follow-up) and who was alive and living in Sweden at age 25 years (n=1,263,896).

p-value <0.001

bbreviation: CI, confidence intervals; OR, odds ratio; USS, upper secondary school.

Table S14. Sensitivity Analysis #3 (if both parents neither died before 1997 nor emigrated prior to 1997and never return to Sweden): educational outcomes in offspring with parents dual- or single affected by five groups of psychiatric disorders (one group at the time). OR (95% CI) for achieving compulsory and post compulsory educational outcomes, compared to individuals with parents from the general population (*uncleaned population*[a]).

	Dual-affected parental pairs				Single-affected parental pairs			
	Offspring of affected pairs	Offspring of unaffected pairs	Crude model	Fully-adjusted[b]	Offspring of affected pairs	Offspring of unaffected pairs	Crude model	Fully-adjusted[b]
	n (%)	n (%)	OR (95% CI)	OR (95% CI)	n (%)	n (%)	OR (95% CI)	OR (95% CI)
**ELIGIBILITY TO ACCESS USS[c]**								
**Neuropsychiatric disorders[d]**	**n=579**	**n=1,353,860**			**n=20,443**	**n=1,333,996**		
achieved	423 (73.06)	1,237,106 (91.38)	0.26 (0.21-0.31)*	0.31 (0.26-0.39)*	16,105 (78.78)	1,221,424 (91.56)	0.34 (0.33-0.35)*	0.39 (0.37-0.40)*
**Substance use disorders**	**n=9,451**	**n=1,344,988**			**n=113,369**	**n=1,241,070**		
achieved	6,773 (71.66)	1,230,756 (91.51)	0.23 (0.22-0.25)*	0.24 (0.23-0.25)*	93,447 (82.43)	1,144,082 (92.19)	0.40 (0.39-0.41)*	0.41 (0.40-0.42)*
**GAD and MDD**	**n=5,963**	**n=1,348,476**			**n=128,471**	**n=1,225,968**		
achieved	4,721 (79.17)	1,232,808 (91.42)	0.36 (0.33-0.38)*	0.38 (0.36-0.41)*	110,432 (85,96)	1,127,097 (91.94)	0.54 (0.53-0.55)*	0.56 (0.55-0.57)*
**AGO, SOC, and OCD**	**n=179**	**n=1,354,260**			**n=18,420**	**n=1,336,019**		
achieved	122 (68.16)	1,237,407 (91.37)	0.20 (0.14-0.29)*	0.24 (0.17-0.35)*	15,241 (82.74)	1,222,288 (91.49)	0.45 (0.43-0.47)*	0.48 (0.46-0.51)*
**SCZ and BIP**	**n=423**	**n=1,354,016**			**n=30,416**	**n=1,324,023**		
achieved	341 (80.61)	1,237,188 (91.37)	0.39 (0.30-0.51)*	0.39 (0.30-0.51)*	26,275 (86.39)	1,211,254 (91.48)	0.59 (0.57-0.61)*	0.61 (0.59-0.63)*
**FINISHING USS[e]**								
**Neuropsychiatric disorders[d]**	**n=471**	**n=1,876,149**			**n=19,797**	**n=1,856,823**		
achieved	179 (38.00)	1,518,207 (80.92)	0.14 (0.12-0.18)*	0.20 (0.16-0.24)*	11,120 (56.17)	1,507,266 (81.17)	0.30 (0.29-0.31)*	0.36 (0.35-0.37)*
**Substance use disorders**	**n=13,980**	**n=1,862,640**			**n=171,911**	**n=1,704,709**		
achieved	7,005 (50.11)	1,511,381 (81.14)	0.23 (0.22-0.24)*	0.23 (0.22-0.24)*	114,351 (66.52)	1,404,035 (82.36)	0.42 (0.42-0.43)*	0.42 (0.41-0.43)*
**GAD and MDD**	**n=7,347**	**n=1,869,273**			**n=170,877**	**n=1,705,743**		
achieved	4,486 (61.02)	1,513,903 (80.99)	0.37 (0.35-0.39)*	0.40 (0.38-0.42)*	122,127 (71.47)	1,396,259 (81.86)	0.55 (0.54-0.56)*	0.57 (0.56-0.58)*
**AGO, SOC, and OCD**	**n=183**	**n=1,876,437**			**n=21,014**	**n=1,855,606**		
achieved	85 (46.45)	1,518,301 (80.91)	0.20 (0.15-0.28)*	0.27 (0.20-0.38)*	13,708 (65.23)	1,504,678 (81.09)	0.44 (0.42-0.45)*	0.49 (0.48-0.51)*
**SCZ and BIP**	**n=595**	**n=1,876,025**			**n=42,565**	**n=1,834,055**		
achieved	364 (61.18)	1,518,022 (80.92)	0.37 (0.31-0.44)*	0.38 (0.32-0.45)*	30,233 (71.03)	1,488,153 (81.14)	0.57 (0.56-0.58)*	0.58 (0.56-0.59)*
**STARTING UNIVERSITY[f]**								
**Neuropsychiatric disorders[d]**	**n=351**	**n=1,680,853**			**n=16,122**	**n=1,665,082**		
achieved	38 (10.83)	648,154 (38.56)	0.19 (0.13-0.28)*	0.32 (0.22-0.47)*	3,489 (21.64)	644,703 (38.72)	0.44 (0.42-0.46)*	0.58 (0.55-0.60)*
**Substance use disorders**	**n=12,730**	**n=1,668,474**			**n=156,937**	**n=1,524,267**		
achieved	1,926 (15.13)	646,266 (38.73)	0.28 (0.27-0.30)*	0.28 (0.27-0.30)*	40,022 (25.20)	608,170 (39.90)	0.51 (0.51-0.52)*	0.51 (0.50-0.52)*
**GAD and MDD**	**n=6,399**	**n=1,674,805**			**n=151,939**	**n=1,529,265**		
achieved	1,684 (26.32)	646,508 (38.60)	0.57 (0.53-0.60)*	0.61 (0.58-0.65)*	48,380 (31.84)	599,812 (39.22)	0.72 (0.71-0.73)*	0.75 (0.74-0.76)*
**AGO, SOC, and OCD**	**n=152**	**n=1,681,052**			**n=18,134**	**n=1,663,070**		
achieved	29 (19.08)	648,163 (38.56)	0.38 (0.24-0.58)*	0.55 (0.35-0.88)#	4,827 (26.62)	643,365 (38.69)	0.57 (0.55-0.60)*	0.67 (0.64-0.70)*
**SCZ and BIP**	**n=531**	**n=1,680,673**			**n=38,235**	**n=1,642,969**		
achieved	161 (30.32)	648,031 (38.56)	0.69 (0.57-0.84)*	0.71 (0.58-0.86)§	13,138 (34.36)	635,054 (38.65)	0.83 (0.81-0.85)*	0.84 (0.82-0.86)*
**FINISHING UNIVERSITY[g]**								

**Neuropsychiatric disorders[d]**	**n=158**	**n=1,263,738**			**n=9,406**	**n=1,254,490**		
achieved	10 (6.33)	330,671 (26.17)	0.19 (0.10-0.36)[*]	0.33 (0.17-0.64)[§]	1,275 (13.56)	329,406 (26.26)	0.44 (0.41-0.47)[*]	0.59 (0.55-0.63)[*]
**Substance use disorders**	**n=9,744**	**n=1,254,152**			**n=121,392**	**n=1,142,504**		
achieved	832 (8.54)	329,849 (26.30)	0.26 (0.24-0.28)[*]	0.27 (0.25-0.29)[*]	19,405 (15.99)	311,276 (27.25)	0.51 (0.50-0.52)[*]	0.51 (0.50-0.52)[*]
**GAD and MDD**	**n=4,489**	**n=1,259,407**			**n=111,121**	**n=1,152,775**		
achieved	780 (17.38)	329,901 (26.19)	0.59 (0.54-0.64)[*]	0.64 (0.59-0.70)[*]	23,549 (21.19)	307,132 (26.64)	0.74 (0.73-0.75)[*]	0.77 (0.76-0.78)[*]
**AGO, SOC, and OCD**	**n=93**	**n=1,263,803**			**n=12,150**	**n=1,251,746**		
achieved	10 (10.75)	330,671 (26.16)	0.34 (0.18-0.64)[§]	0.49 (0.25-0.97)[#]	2,101 (17.29)	328,580 (26.25)	0.59 (0.56-0.62)[*]	0.68 (0.65-0.72)[*]
**SCZ and BIP**	**n=379**	**n=1,263,517**			**n=28,552**	**n=1,235,344**		
achieved	60 (15.83)	330,621 (26.17)	0.53 (0.40-0.70)[*]	0.55 (0.41-0.73)[*]	6,564 (22.99)	324,117 (26.24)	0.84 (0.81-0.87)[*]	0.85 (0.83-0.88)[*]

Note: All models are clustered by family identification number with robust standard error estimation (sandwich estimator). Within each group, *'dual-affected parental pairs'* imply that both parents have records of the disorders from the group in question (both parents diagnosed with either the same or different disorders within the group; for example, attention-deficit hyperactivity disorder, or autism spectrum disorder, or Tourette's disorder and chronic tic disorders in the analysis of neuropsychiatric disorders). Within each group, *'single-affected parental pairs'* imply that one parent has at least one disorder from the group in question and the other parent is free from any disorders from the corresponding group. For each educational outcome, separate sub-cohorts of index offspring were created, comprising the individuals who had the time necessary to achieve the corresponding outcome.

[a] 'Uncleaned population' imposes no restriction on parental diagnoses in the comparison group and represents, in each relevant analysis, the rest of study population. This approach reflects the high rates of psychiatric comorbidity and ensures obtaining real-world, conservative measures of associations.
[b] Adjusted for offspring year of birth (5-year categories starting from 1973), offspring sex, and maternal and paternal year of birth in decades (<1940, 1950s, 1960s, 1970s, ≥1970)
[c] Offspring who graduated from compulsory school in 1998-2013 (n=1,354,439). The graduation years were chosen due to the different eligibility criteria used prior to 1998.
[d] Neuropsychiatric disorder group includes attention-deficit hyperactivity disorder, autism spectrum disorder, and Tourette's disorder and chronic tic disorders.
[e] Offspring born in 1973-1994 (i.e., aged ≥19 by the end of follow-up) and who was alive and living in Sweden at age 19 years (n=1,876,620).
[f] Offspring born in 1973-1992 (i.e., aged ≥21 by the end of follow-up) and who was alive and living in Sweden at age 21 years (n=1,681,204).
[g] Offspring born in 1973-1988 (i.e., aged ≥25 by the end of follow-up) and who was alive and living in Sweden at age 25 years (n=1,263,896).

[#] p-value <0.05; [§] p-value <0.01; [*] p-value <0.001

Abbreviation: ADHD, attention-deficit hyperactivity disorder; AGO, agoraphobia; ASD, autism spectrum disorder; BIP, bipolar disorder; CI, confidence intervals; GAD, generalized anxiety disorder; MDD, major depressive disorder; OCD, obsessive-compulsive disorder; OR, odds ratio; SCZ, schizophrenia; SOC, social phobia; SUD, substance use disorders; TD/CTD, Tourette's disorder and chronic tic disorders; USS, upper secondary school

Table S15. Sensitivity Analysis #3 (if both parents neither died before 1997 nor emigrated prior to 1997and never return to Sweden): educational outcomes in offspring with parents single-affected by each specific disorder (one disorder at the time). OR (95% CI) for achieving compulsory and post compulsory educational outcomes, compared to individuals with parents from the general population (*uncleaned population*[a]).

	Offspring of affected pairs	Offspring of unaffected pairs	Fully-adjusted[b]
	n (%)	n (%)	OR (95% CI)
**Eligibility to access USS[c]**			
ADHD	14,929 (78.46)	1,222,600 (91.55)	0.38 (0.37-0.40)*
ASD	2,010 (80.24)	1,235,519 (91.39)	0.43 (0.38-0.48)*
SCZ	4,726 (83.20)	1,232,803 (91.40)	0.47 (0.44-0.51)*
BIP	22,946 (86.93)	1,214,583 (91.46)	0.64 (0.61-0.67)*
MDD	105,674 (86.00)	1,131,855 (91.90)	0.56 (0.55-0.57)*
GAD	13,562 (83.81)	1,223,967 (91.46)	0.51 (0.49-0.53)*
AGO	4,838 (80.17)	1,232,691 (91.42)	0.42 (0.39-0.45)*
SOC	7,031 (80.90)	1,230,498 (91.44)	0.43 (0.41-0.46)*
OCD	5,489 (85.30)	1,232,040 (91.40)	0.59 (0.55-0.64)*
SUD	93,447 (82.43)	1,144,082 (92.19)	0.41 (0.40-0.42)*
TD/CTD	418 (81.80)	1,237,111 (91.37)	0.47 (0.37-0.61)*
**Finishing USS[d]**			
ADHD	10,148 (55.56)	1,508,238 (81.16)	0.35 (0.34-0.37)*
ASD	1,405 (57.99)	1,516,981 (80.94)	0.39 (0.36-0.43)*
SCZ	6,335 (67.78)	1,512,051 (80.98)	0.49 (0.46-0.51)*
BIP	25,954 (71.77)	1,492,432 (81.09)	0.60 (0.59-0.62)*
MDD	117,137 (71.51)	1,401,249 (81.81)	0.58 (0.57-0.59)*
GAD	13,761 (67.75)	1,504,625 (81.05)	0.53 (0.51-0.54)*
AGO	4,145 (61.37)	1,514,241 (80.98)	0.43 (0.41-0.46)*
SOC	5,951 (62.07)	1,512,435 (81.01)	0.44 (0.42-0.46)*
OCD	5,235 (69.93)	1,513,151 (80.95)	0.60 (0.57-0.63)*
SUD	114,351 (66.52)	1,404,035 (82.36)	0.42 (0.41-0.43)*
TD/CTD	370 (64.72)	1,518,016 (80.92)	0.50 (0.41-0.62)*
**Staring university[e]**			
ADHD	3,118 (21.04)	645,074 (38.71)	0.56 (0.54-0.59)*
ASD	460 (23.30)	647,732 (38.57)	0.63 (0.56-0.71)*
SCZ	2,696 (31.15)	645,496 (38.59)	0.71 (0.67-0.74)*
BIP	11,359(35.14)	636,833 (38.62)	0.88 (0.85-0.90)*
MDD	46,551 (31.95)	601,641 (39.18)	0.76 (0.75-0.77)*

GAD	5,133 (28.70)	643,059 (38.66)	0.70 (0.67-0.72)*
AGO	1,311 (22.54)	646,881 (38.61)	0.56 (0.52-0.60)*
SOC	1,928 (23.62)	646,264 (38.63)	0.60 (0.56-0.63)*
OCD	2,059 (31.78)	646,133 (38.58)	0.83 (0.78-0.88)*
SUD	40,022 (25.20)	608,170 (39.90)	0.51 (0.50-0.52)*
TD/CTD	145 (29.59)	648,047 (38.56)	0.75 (0.60-0.94)#
**Finishing university[f]**			
ADHD	1,129 (13.22)	329,552 (26.25)	0.58 (0.54-0.62)*
ASD	160 (13.40)	330,521 (26.18)	0.59 (0.49-0.70)*
SCZ	1,314 (19.22)	329,367 (26.20)	0.67 (0.63-0.71)*
BIP	5,704 (23.83)	324,977 (26.21)	0.90 (0.87-0.93)*
MDD	22,736 (21.33)	307,945 (26.61)	0.78 (0.76-0.79)*
GAD	2,354 (18.44)	328,327 (26.24)	0.70 (0.66-0.73)*
AGO	531 (13.88)	330,150 (26.20)	0.55 (0.50-0.61)*
SOC	808 (15.10)	329,873 (26.21)	0.62 (0.57-0.67)*
OCD	944 (21.32)	329,737 (26.18)	0.83 (0.77-0.90)*
SUD	19,405 (15.99)	311,276 (27.25)	0.51 (0.50-0.52)*
TD/CTD	59 (18.21)	330,622 (26.17)	0.69 (0.51-0.94)#

Note: All models are clustered by family identification number with robust standard error estimation (sandwich estimator). For each disorder, *'single-affected parental pairs'* imply that one parent (either mother or father) has a disorder in question and the other parent is free from such disorder. For each educational outcome, separate sub-cohorts of index offspring were created, comprising the individuals who had the time necessary to achieve the corresponding outcome.

[a] 'Uncleaned population' imposes no restriction on parental diagnoses in the comparison group and represents, in each relevant analysis, the rest of study population. This approach reflects the high rates of psychiatric comorbidity and ensures obtaining real-world, conservative measures of associations.
[b] Adjusted for offspring year of birth (5-year categories starting from 1973), offspring sex, and maternal and paternal year of birth in decades (<1940, 1950s, 1960s, 1970s, ≥1970)
[c] Offspring who graduated from compulsory school in 1998-2013 (n=1,354,439). The graduation years were chosen due to the different eligibility criteria used prior to 1998.
[d] Offspring born in 1973-1994 (i.e., aged ≥19 by the end of follow-up) and who was alive and living in Sweden at age 19 years (n=1,876,620).
[e] Offspring born in 1973-1992 (i.e., aged ≥21 by the end of follow-up) and who was alive and living in Sweden at age 21 years (n=1,681,204).
[f] Offspring born in 1973-1988 (i.e., aged ≥25 by the end of follow-up) and who was alive and living in Sweden at age 25 years (n=1,263,896).

# p-value <0.05; § p-value <0.01; * p-value <0.001

Abbreviation: ADHD, attention-deficit hyperactivity disorder; AGO, agoraphobia; ASD, autism spectrum disorder; BIP, bipolar disorder; CI, confidence intervals; GAD, generalized anxiety disorder; MDD, major depressive disorder; OCD, obsessive-compulsive disorder; OR, odds ratio; SCZ, schizophrenia; SOC, social phobia; SUD, substance use disorders; TD/CTD, Tourette's disorder and chronic tic disorders; USS, upper secondary school.

eReferences

Benchimol EI, Smeeth L, Guttmann A, Harron K, Moher D, Petersen I, Sørensen HT, von Elm E, Langan SM, the RECORD Working Committee. The REporting of studies Conducted using Observational Routinely-collected health Data (RECORD) Statement. PLoS Medicine 2015; 12(10): e1001885. https://doi.org/10.1371/journal.pmed.1001885

Brander G, Rydell M, Kuja-Halkola R, Fernandez de la Cruz, L., Lichtenstein, P., Serlachius, E., . . . Mataix-Cols, Det al. (2018). Perinatal risk factors in Tourette's and chronic tic disorders: a total population sibling comparison study. Mol Psychiatry,. 2018; 23(5),:1189- 1197.

Brooke, H. L., Talback, M., Hornblad, J., Johansson, L. A., Ludvigsson, J. F., Druid, H., . . . Ljung, R. (2017). The Swedish cause of death register. Eur J Epidemiol, 32(9), 765-773. doi: 10.1007/s10654-017-0316-1

Deen, J. (2007). Higher education in Sweden. IHEM Country report. 2007: Enschede, the Netherlands: Center for Higher Education Policy Studies (CHEPS). https://www.wissenschaftsmanagement-online.de/sites/www.wissenschaftsmanagement-online.de/files/migrated_wimoarticle/2007countryreportsweden.pdf. Accessed December 22, 2020October 17, 2022.

Ekbom, A. (2011). The Swedish Multi-generation Register. Methods Mol Biol, 675, 215-220. doi: 10.1007/978-1-59745-423-0_10

Fernandez de la Cruz, L., Rydell, M., Runeson, B. Brander, G., Ruck, C., D'Onofrio, B. M., . . . Mataix-Cols, Det al. (2017). Suicide in Tourette's and Chronic Tic Disorders. Biol Psychiatry, . 2017;82(2), 111-118.

Larsson, H., Ryden, E., Boman, M., Langstrom, N., Lichtenstein, P., & Landen, M. (2013). Risk of bipolar disorder and schizophrenia in relatives of people with attention-deficit hyperactivity disorder. Br J Psychiatry, 203(2), 103-106. doi: 10.1192/bjp.bp.112.120808Larsson H, Ryden E, Boman M, Langstrom N, Lichtenstein P, Landen M. Risk of bipolar disorder and schizophrenia in relatives of people with attention-deficit hyperactivity disorder. Br J Psychiatry. 2013;203(2):103-106.

Ludvigsson, J. F., Almqvist, C., Bonamy, A. K., Ljung, R., Michaelsson, K., Neovius, M., . . . Ye, W. (2016). Registers of the Swedish total population and their use in medical research. Eur J Epidemiol, 31(2), 125-136. doi: 10.1007/s10654-016-0117-y

Ludvigsson, J. F., Andersson, E., Ekbom, A., Feychting, M., Kim, J. L., Reuterwall, C., . . . Olausson, P. O. (2011). External review and validation of the Swedish national inpatient register. BMC Public Health, 11, 450. doi: 10.1186/1471-2458-11-450

Ludvigsson, J. F., Svedberg, P., Olen, O., Bruze, G., & Neovius, M. (2019). The longitudinal integrated database for health insurance and labour market studies (LISA) and its use in medical research. Eur J Epidemiol, 34(4), 423-437. doi: 10.1007/s10654-019-00511-8Ludvigsson JF, Svedberg P, Olen O, Bruze G, Neovius M. The longitudinal integrated database for health insurance and labour market studies (LISA) and its use in medical research. *Eur J Epidemiol*. 2019;34(4):423-437.

Mataix-Cols, D., Isomura, K., Perez-Vigil, A., Chang, Z., Ruck, C., Larsson, K. J.,et al . . . Lichtenstein, P. (2015). Familial Risks of Tourette Syndrome and Chronic Tic Disorders. A Population-Based Cohort Study. JAMA Psychiatry,. 2015; 72(8),787-793.

Perez-Vigil, A., Fernandez de la Cruz, L., Brander, G., Isomura, K., Jangmo, A., Kuja-Halkola, R., . . . Mataix-Cols, D. (2018). Association of Tourette Syndrome and Chronic Tic Disorders With

Objective Indicators of Educational Attainment: A Population-Based Sibling Comparison Study. JAMA Neurol, 75(9), 1098-1105. doi: 10.1001/jamaneurol.2018.1194Perez-Vigil A, Fernández de la Cruz L, Brander G, et al. Association of Tourette Syndrome and Chronic Tic Disorders With Objective Indicators of Educational Attainment: A Population-Based Sibling Comparison Study. JAMA Neurol. 2018;75(9):1098-1105.
Ruck, C., Larsson, K. J., Lind, K., Perez-Vigil, A., Isomura, K., Sariaslan, A., . . . Mataix-Cols, D. (2015). Validity and reliability of chronic tic disorder and obsessive-compulsive disorder diagnoses in the Swedish National Patient Register. BMJ Open, 5(6), e007520. doi: 10.1136/bmjopen-2014-007520Ruck C, Larsson KJ, Lind K, et al. Validity and reliability of chronic tic disorder and obsessive-compulsive disorder diagnoses in the Swedish National Patient Register. BMJ Open. 2015;5(6):e007520.
The Swedish National Agency for Education (2018a). National School Register. 2018. https://www.skolverket.se/. Accessed October 17, 2022.
The Swedish National Agency for Education (2018b). Curriculum for the compulsory school, preschool class and school-age educare 2011 (revised 2018). 2018.https://www.skolverket.se/publikationsserier/styrdokument/2018/. Accessed October 17, 2022.
The Swedish National Agency for Education. (2000). Education for all. Swedish education system. Lenanders Tryckeri AB: Kalmar, Sweden. 2000. https://www.skolverket.se. Accessed October 17, 2022.
The Swedish National Agency for Education. (2013). Curriculum for the upper secondary school. 2013. https://www.skolverket.se/publikationsserier/styrdokument/2013/. Accessed October 17, 2022.
The Swedish National Agency for Education. (2022). Sätta betyg i grundskolan [Grading in elementary school]. https://www.skolverket.se/undervisning/grundskolan/betyg-i-grundskolan/satta-betyg-i-grundskolan. Accessed October 17, 2022.
Upper secondary education. (2020). https://utbildningsguiden.skolverket.se/languages/english-engelska/upper-secondary-education. Accessed October 17, 2022.
Willemse, N., & P. De Beer P. (2012). Three worlds of educational welfare states? A comparative study of higher education systems across welfare states. *Journal of European Social Policy,* 22(2),105–117.
1.
